# A systematic survey of centrality measures for protein-protein interaction networks

**DOI:** 10.1186/s12918-018-0598-2

**Published:** 2018-07-31

**Authors:** Minoo Ashtiani, Ali Salehzadeh-Yazdi, Zahra Razaghi-Moghadam, Holger Hennig, Olaf Wolkenhauer, Mehdi Mirzaie, Mohieddin Jafari

**Affiliations:** 1 Drug Design and Bioinformatics Unit, Medical Biotechnology Department, Biotechnology Research Center, Pasteur Institute of Iran, P.O. Box 13164, Tehran, Iran; 2 Department of Systems Biology and Bioinformatics, University of Rostock, P.O. Box 18051, Rostock, Germany; 3 Faculty of New Sciences and Technologies, University of Tehran, P.O. Box 143995-71, Tehran, Iran; 4 Max-Planck Institute of Molecular Plant Physiology, Potsdam, Germany; 5 Department of Applied Mathematics, Faculty of Mathematical Sciences, Tarbiat Modares University, P.O. Box 14115-134, Tehran, Iran

**Keywords:** Network science, Centrality analysis, Protein-protein interaction network (PPIN), Clustering, Principal components analysis (PCA)

## Abstract

**Background:**

Numerous centrality measures have been introduced to identify “central” nodes in large networks. The availability of a wide range of measures for ranking influential nodes leaves the user to decide which measure may best suit the analysis of a given network. The choice of a suitable measure is furthermore complicated by the impact of the network topology on ranking influential nodes by centrality measures. To approach this problem systematically, we examined the centrality profile of nodes of yeast protein-protein interaction networks (PPINs) in order to detect which centrality measure is succeeding in predicting influential proteins. We studied how different topological network features are reflected in a large set of commonly used centrality measures.

**Results:**

We used yeast PPINs to compare 27 common of centrality measures. The measures characterize and assort influential nodes of the networks. We applied principal component analysis (PCA) and hierarchical clustering and found that the most informative measures depend on the network’s topology. Interestingly, some measures had a high level of contribution in comparison to others in all PPINs, namely Latora closeness, Decay, Lin, Freeman closeness, Diffusion, Residual closeness and Average distance centralities.

**Conclusions:**

The choice of a suitable set of centrality measures is crucial for inferring important functional properties of a network. We concluded that undertaking data reduction using unsupervised machine learning methods helps to choose appropriate variables (centrality measures). Hence, we proposed identifying the contribution proportions of the centrality measures with PCA as a prerequisite step of network analysis before inferring functional consequences, e.g., essentiality of a node.

**Electronic supplementary material:**

The online version of this article (10.1186/s12918-018-0598-2) contains supplementary material, which is available to authorized users.

## Background

Essential proteins play critical roles in cell processes such as development and survival. Deletion of essential proteins is more likely to be lethal than deletion of non-essential proteins [1]. Identifying essential proteins conventionally had been carried out with experimental methods which are time-consuming and expensive, and such experimental approaches are not always feasible. Analyzing high-throughput data with computational methods promises to overcome these limitations. Various computational methods have been proposed to predict and prioritize influential nodes (e.g. proteins) among biological networks. Network-based ranking (i.e. centrality analysis) of biological components has been widely used to find influential nodes in large networks, with applications in biomarker discovery, drug design and drug repurposing [2–6]. Not only in molecular biology networks but also in all types of networks, finding the influential nodes is the chief question of centrality analysis [7]. Examples include predicting the details of information controlling or disease spreading within a specific network in order to delineate how to effectively implement target marketing or preventive healthcare [8–10]. Several centralities measures (mostly in the context of social network analyses) have been described [7] in the last decades. A comprehensive list of centrality measures and software resources can be found on the CentiServer [11].

The correlation of lethality and essentiality with different centrality measures has been subject of active research in biological areas, which has led to the centrality-lethality rule [1]. Typically, some classic centrality measures such as Degree, Closeness, and Betweenness centralities have been utilized to identify influential nodes in biological networks [9]. For example, in a pioneering work, the authors found that proteins with the high Degree centrality (hubs) among a yeast PPIN is likely to be associated with essential proteins [1]. In another study, this rule was re-examined in three distinct PPINs of three species which confirmed the essentiality of highly connected proteins for survival [12]. Similar results were reported for gene co-expression networks of three different species [13] and for metabolic network of *Escherichia coli* [14, 15]. Ernesto Estrada generalized this rule to six other centrality measures. He showed that the Subgraph centrality measure scored best compared to classic measures to find influential proteins, and generally using these measures performed significantly better than a random selection [16]. However, He and Zhang showed that the relationship between hub nodes and essentiality is not related to the network architecture [17]. Furthermore, regarding the modular structure of PPINs, Joy et al. concluded that the Betweenness centrality is more likely to be essential than the Degree centrality [18]. The predictive power of Betweenness as a topological characteristic was also mentioned in mammalian transcriptional regulatory networks which was clearly correlated to Degree [19]. Recently, it has been shown that presence of hubs, i.e. high Degree centralities, do not have a direct relationship with prognostic genes across cancer types [20].

On the other hand, Tew and Li demonstrated functional centrality and showed that it correlates more strongly than pure topological centrality [21]. More recently, localization-specific centrality measures had been introduced and claimed that their results is more likely essential in different species [22–25]. In the same way, some studies emphasized on the protein complex and topological structure of a sub-network to refine PPIN and identify central nodes [26–28]. Tang et al. integrated the gene co-expression data on PPIN as edge weights to realize the reliable prediction of essential proteins [24]. Khuri and Wuchty introduced minimum dominating sets of PPIN which are enriched by essential proteins. They described that there is a positive correlation between Degree of proteins in these sets and lethality [29]. In these studies, the solution of the controversy is ascribed to utilizing biological information.

Similar in methodology but different in the underlying physical system that the network represents, some other studies attempted to quantify correlations between several classic centrality measures. In 2004, Koschützki and Schreiber compared five centrality measures in two biological networks and showed different patterns of correlations between centralities. They generally concluded that all Degree, Eccentrecity, Closeness, random walk Betweenness and Bonacich’s Eigenvector centralities should be considered to find central nodes and could be useful in various applications without explaining any preference among them [30]. Two years later, they re-expressed pervious outcomes by explaining the independence behavior of centrality measures in a PPIN using 3D parallel coordinates, orbit-based and hierarchy-based comparison [31]. Valente et al. examined the correlation between the symmetric and directed versions of four measures which are commonly used by the network analysts. By comparing 58 different social networks, they concluded that network data collection methods change the correlation between the measures and these measures show distinct trends [32]. Batool and Niazi also studied three social, ecological and biological neural networks and they concluded the correlation between Closeness-Eccentricity and Degree-Eigenvector and insignificant pattern of Betweenness. They also demonstrated that Eccentricity and Eigenvector measures are better to identify influential nodes [33]. In 2015, Cong Li et al. further investigated the question of correlation between centrality measures and introduced a modified centrality measure called *m*th-order degree mass. They observed a strong linear correlation between the Degree, Betweenness and Leverage centrality measures within both real and random networks [34].

However, there is no benchmark for network biologists that provides insight, which of the centrality measures is suited best for the analysis of the given network. The result of the centrality analysis of a network may depend on the used centrality measure which can lead to inconsistent outcomes. Previously, a detailed study showed that the predictive power and shortcomings of centrality measures are not satisfactory in various studies [35]. While these centrality measures have proven to be essential in understanding of the roles of nodes which led to outstanding contributions to the analysis of biological networks, choosing the appropriate measure for given networks is still an open question. Which measure identifies best the centers of real networks? Do all measures independently highlight the central network elements and encompass independent information or are the measures correlated? Is the computation of all these measures meaningful in all different networks or does the best measure depend on the network topology and the logic of the network reconstruction? In this study, we used unsupervised machine learning to compare how well the most common centrality measures characterize nodes in networks. We comprehensively compared 27 distinct centrality measures applied to 14 small to large biological and random networks. All biological networks were PPINs of the same set of proteins which are reconstructed using a variety of computational and experimental methods. We demonstrated how the ranking of nodes depends on the network structure (topology) and why this network concept i.e. centrality deserves renewed attention.

## Methods

**Fig. 1:**
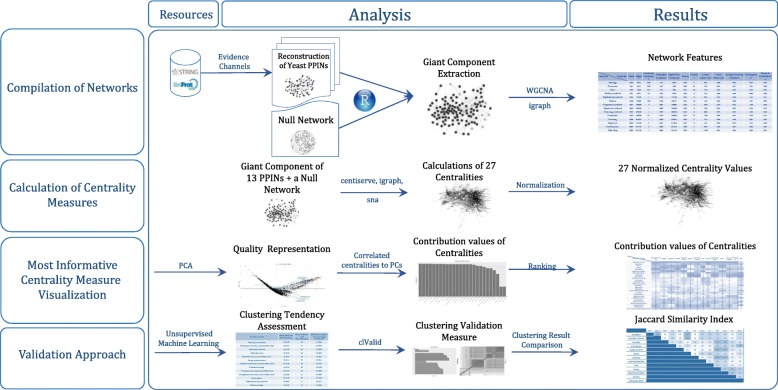
Our workflow for studying the centrality measures. This was followed the reconstruction of the yeast PPIN relying on different kinds of evidence channels as well as the generation of a null network. The workflow contained a comparison of several centrality measures using machine learning methods such as principal components analysis and clustering procedures

The workflow of this study was schematically presented in Fig. 1. Our workflow started by constructing and retrieving networks, followed by global network analysis. The centrality analysis and comparing them using machine learning methods were the next main steps. See basic definitions for more details.

### Reconstruction of the networks

In this study, a UniProtKB reviewed dataset [36] was used to retrieve proteins in *Saccharomyces cerevisiae* (6721 proteins). UniProtKB accessions were converted to STRING using the STRINGdb R package, which resulted in 6603 protein identifiers (3rd Sep 2016). Interactions among proteins were extracted based on the STRING IDs. In the 2017 edition of the STRING database the results of these interactions are structured in a way to provide maximum coverage; this is achieved by including indirect and predicted interactions on the top of the set. [37]. In this study, 13 evidence channels (related to the origin and type of evidence) indicating PPIN of yeast were presented: co-expression, co-expression-transferred, co-occurrence, database, database-transferred, experiments, experiments-transferred, fusion, homology, neighborhood-transferred, textmining, textmining-transferred and combined-score (See Additional file 1). In the following, the name of the reconstructed network is basis of the corresponding channel name which made of. For the purpose of comparison with real network behavior, a null model network was generated. The null network is the Erdős–Rényi model [38] and was generated using the igraph R package [39]. The generated null network was created with a size similar to the yeast reconstructed PPIN in order to have a more fair comparison.

### Fundamental network concepts analysis

To understand the network structure, we reviewed various network features using several R packages [40–42]. The network density, clustering coefficient, network heterogeneity, and network centralization properties of the network were calculated. The number of connected components and graph diameter for each network were also computed. Then, the power-law distribution was assessed by computing α values and r correlation coefficients. As most of centrality measures require a strongly connected component graph, the giant component of each PPINs and the null network were extracted. Moreover, for a general overview of the structure of the extracted giant components, some network features such as network density, clustering coefficient, network heterogeneity, and network centralization were calculated.

### Centrality analysis

For this research study, we were only considered undirected, loop-free connected graphs according to the PPIN topology. For centrality analysis, the following 27 centrality measures were selected: Average Distance [43], Barycenter [44], Closeness (Freeman) [9], Closeness (Latora) [45], Residual closeness [46], ClusterRank [47], Decay [48], Diffusion degree [49], Density of Maximum Neighborhood Component (DMNC) [50], Geodesic K-Path [51, 52], Katz [53, 54], Laplacian [55], Leverage [56], Lin [57], Lobby [58], Markov [59], Maximum Neighborhood Component (MNC) [50], Radiality [60], Eigenvector [61], Subgraph scores [62], Shortest-Paths betweenness [9], Eccentricity [63], Degree, Kleinberg’s authority scores [64], Kleinberg’s hub scores [64], Harary graph [63] and Information [65]. All these measures are calculated for undirected networks in a reasonable time. These measures were calculated using the centiserve [11], igraph [39] and sna [66] R packages. Some of the centrality measures had a measurable factor to be specified which we used the default values. For a better visualization, We assorted the centrality measures into five distinct classes including Distance-, Degree-, Eigen-, Neighborhood-based and miscellaneous groups depend on their logic and formulas (Table 1).

**Table 1:** Centrality measures. The centrality measures were represented in five groups depending on their logic and formulae

Distance_based	Degree-based	Eigen-based	Neighborhood-based	Miscellanous
Average Distance	Authority_score	Eigenvector centralities	ClusterRank	Geodesic K-Path Centrality
Barycenter	Degree Centrality	Katz Centrality (Katz Status Index)	Density of Maximum Neighborhood Component (DMNC)	Harary Graph Centrality
Closeness Centrality (Freeman)	Diffusion Degree	Laplacian Centrality	Maximum Neighborhood Component (MNC)	Information Centrality
Closeness centrality (Latora)	Kleinberg’s hub centrality scores		Subgraph centrality scores	Markov Centrality
Decay Centrality	Leverage Centrality			Shortest-Paths Betweenness Centrality
Eccentricity of the vertices	Lobby Index (Centrality)			
Lin Centrality				
Radiality Centrality				
Residual Closeness Centrality				

Note that the first column (i.e. distance-based centralities) was specified according to the definition of distance between vertices in graph theory. The second one (i.e. degree-based centralities) was defined based on the number of immediate neighbors of each node within a given network. Eigen-values of adjacency matrix was the main idea to classify the Eigen-based centralities. Furthermore, the concept of subgraph or community structure was proposed in the neighborhood-based centralities. Others were collected in the miscellaneous group. Remind that this grouping was just applied to have better visualizations.

### Unsupervised machine learning analysis

Standard normalization (scaling and centering of matrix-like objects) has been undertaken on computed centrality values according to methodology explained in [67]. We used PCA, a linear dimensionality reduction algorithm, [68] as a key step to understand which centrality measures better determine central nodes within a network. PCA was done on normalized computed centrality measures. To validate the PCA results in PPINs, we also examined whether the centrality measures in all networks can be clustered according to clustering tendency procedure. To do this, the Hopkins’ statistic values and visualizing VAT (Visual Assessment of cluster Tendency) plots was calculated by factoextra R package [69]. We applied the clustering validation measures to access the most appropriate clustering method among hierarchical, k-means, and PAM (Partitioning Around Medoids) methods using clValid package [70]. This provides silhouette scores according to clustering measures which would be helpful for choosing the suitable method. After selection of the clustering technique, factoextra package was used to attain optimal number of clusters [69]. In order to measure the dissimilarity among clusters, we used Ward’s minimum variance method. To compare the clustering results in aforementioned PPINs, the Jaccard similarity index was used relying on the similarity metrics of the clustering results within BiRewire package [71].

## Results

### Evaluation of network properties

**Fig. 2:**
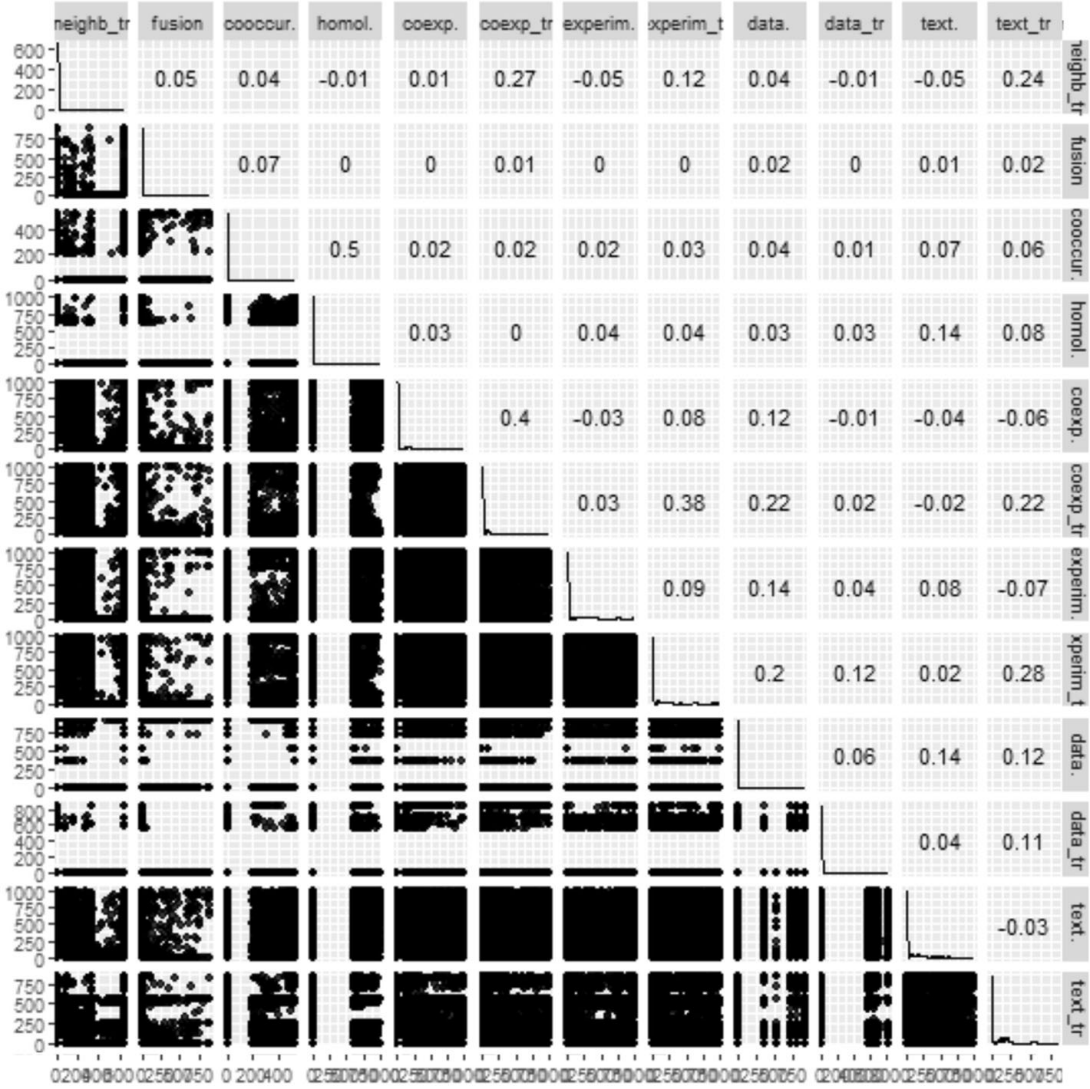
Pairwise scatterplot between the evidence channel scores. The Pearson’s r correlation coefficients between the evidence channels were shown in the upper triangle of the plot. The distributions of scores in each evidence were presented at the diameters of the figure

By importing the same set of protein names, the 13 PPINs were extracted from the STRING database using different evidence channels. (Note: the PPI scores derived from the neighborhood channel of yeast were all zero). All these channels distinctly identify an interaction for each protein pair quantitatively. The dependency between evidence channels was also shown in Fig. 2 by a pairwise scatterplot and Pearson’s r correlation coefficient. Most of the networks were not significantly correlated and correlation coefficients were around zero for all networks.

**Fig. 3:**
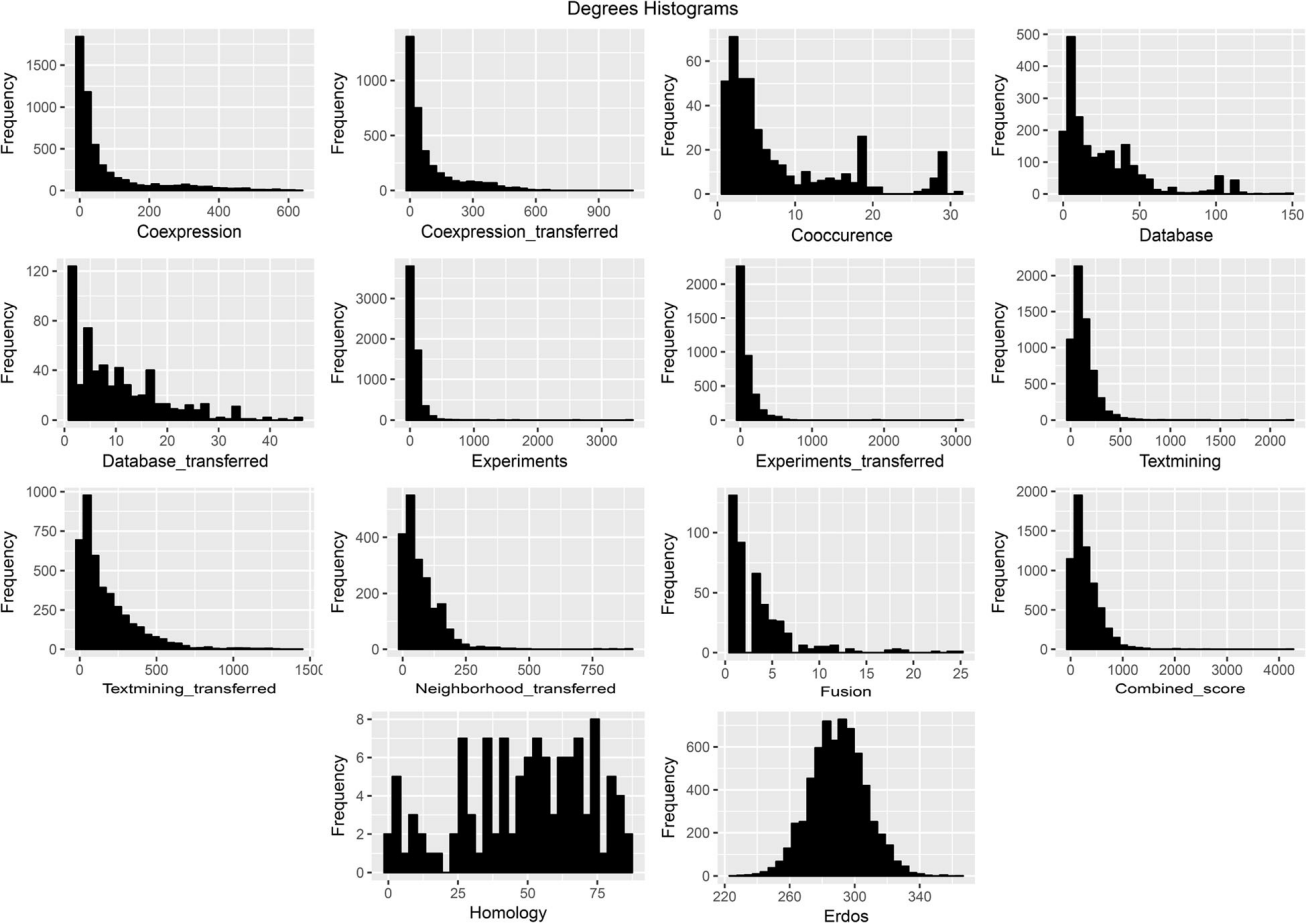
Graphical representation of the Degree distributions in each reconstructed PPIN and the generated null network

**Fig. 4:**
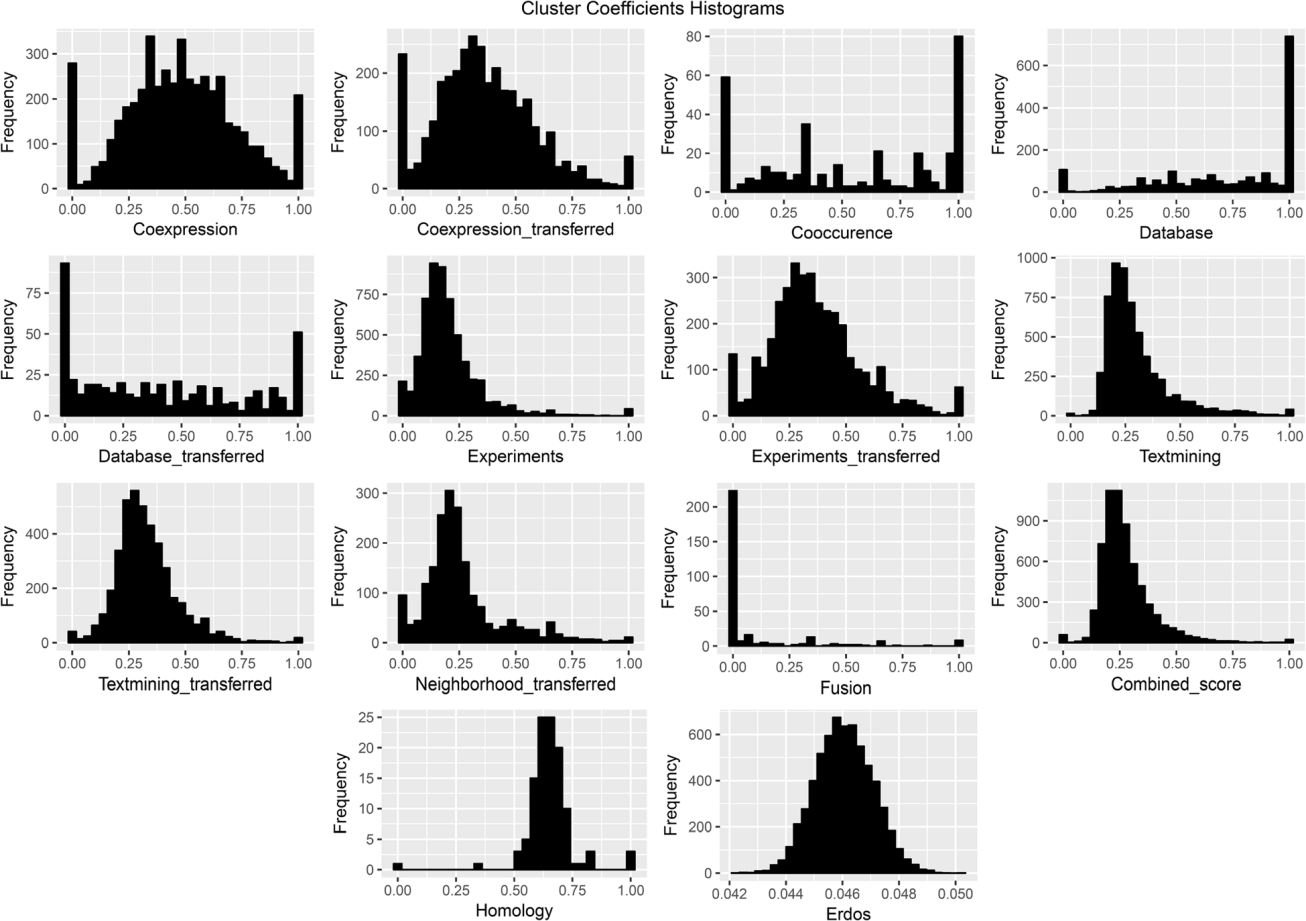
Graphical representation of the clustering coefficient distributions in each reconstructed PPIN and the generated null network

In the following, the 14 networks were utilized to undertake an examination of centrality measures. Note that the giant component of each network was accounted for computing several network properties (Table 2). The homology, fusion, co-occurrence and database networks contained high numbers of unconnected components. Except the homology network which had the smallest giant component, the densities of all networks were between 0.01–0.05, as was expected real network are typically sparse. The network diameter of the fusion, co-occurrence, database and co-expression were one order of magnitude greater than others. All of the PPINs except homology network were correlated to power-law distribution with high r correlation coefficients and diverse alpha power (see Additional file 2). The high value of the average clustering coefficients of the database and homology indicated the modular structure of these networks. Compared with the null network, most of the PPINs had a high value of heterogeneity and network centralization. The Degree distribution and clustering coefficients for the networks were also plotted in Figs. 3 and 4 respectively. Except the homology network, all the Degree distributions were left-skewed similar to scale-free networks. The dependency of PPINs was further assessed and confirmed statistically by Wilcoxon rank sum test (Table 3).

**Table 2:** Network global properties of all PPINs and the null network

Networks Properties	Nodes	Edges	Connected Components	Nodes/giant component	Edges/Giant Component	Density	Diameter	α value (Power Law)	r value (Power Law)	Average Clustering Coefficent	Heterogeneity	Network Centralization
Homology	2479	7545	648	115	2813	0.43	5	0.01	0.00	0.64	0.47	0.34
Cooccurence	1221	3275	209	425	1653	0.02	22	1.07	0.61	0.47	1.02	0.06
Fusion	1187	1408	222	437	789	0.01	26	1.76	0.91	0.07	1.00	0.05
Database_transferred	622	2975	10	583	2930	0.02	9	1.23	0.69	0.36	0.86	0.06
Neighborhood_transferred	2004	71,236	1	2004	71,236	0.04	6	0.91	0.72	0.26	1.04	0.41
Database	2496	27,766	100	2058	26,574	0.01	20	1.18	0.56	0.69	1.07	0.06
Coexpression_transferred	3614	168,368	4	3607	168,364	0.03	8	0.96	0.79	0.35	1.39	0.26
Experiments_transferred	3870	163,403	1	3870	163,403	0.02	6	1.03	0.81	0.35	1.59	0.77
Textmining_transferred	4207	364,816	1	4207	364,816	0.04	6	0.88	0.75	0.32	1.09	0.30
Coexpression	5310	195,676	27	5254	195,643	0.01	15	1.08	0.82	0.44	1.56	0.11
Textmining	5896	379,341	1	5896	379,341	0.02	5	1.01	0.66	0.30	0.92	0.35
Experiments	6026	211,613	2	6024	211,612	0.01	6	1.22	0.82	0.19	1.54	0.56
Combined_score	6294	911,414	2	6292	911,413	0.05	7	0.76	0.63	0.27	0.90	0.62
Erdős_Rényi	6292	911,413	1	6292	911,413	0.05	3	_1.095	0.01	0.05	0.06	0.01

**Table 3:** The *p*-value of Wilcoxon rank sum test. The dependency between the distributions of evidence channels evaluated by Wilcoxon test

	coexpression	coexpression_transferred	cooccurence	database	database_transferred	experiments	experiments_transferred	textmining	textmining_transferred	neighborhood_transferred	fusion	combined_score	homology
coexpression		1.02E-20	3.26E-69	2.51E-23	2.78E-61	5.01E-53	5.91E-38	< 0.1E + 300	< 0.1E + 300	6.02E-46	3.68E-130	< 0.1E + 300	3.49E-05
coexpression_transferred			2.92E-99	7.95E-73	1.188E-96	0.01	0.02	2.91E-186	3.23E-170	4.41E-07	4.23E-159	< 0.1E + 300	0.19
cooccurence				1.69E-59	1.123E-06	6.97E-147	4.023E-128	1.37E-228	4.02E-201	3.69E-143	7.28E-20	1.522E-229	1.06E-45
database					6.11E-41	8.05E-163	2.93E-118	< 0.1E + 300	< 0.1E + 300	1.84E-144	2.33E-127	< 0.1E + 300	1.09E-22
database_transferred						5.06E-167	1.48E-135	1.57E-290	4.48E-248	1.34E-159	1.01E-45	6.46E-299	1.27E-45
experiments							0.91	< 0.1E + 300	9.11E-269	6.52E-06	9.60E-200	< 0.1E + 300	0.37
experiments_transferred								7.3486E-217	9.63E-187	0.00	1.23E-184	< 0.1E + 300	0.37
textmining									1.03E-07	1.32E-134	1.18E-255	< 0.1E + 300	5.39E-21
textmining_transferred										4.78E-120	1.17E-233	2.19E-166	5.63E-16
neighborhood_transferred											1.25E-189	< 0.1E + 300	0.28
fusion												8.63E-249	4.22E-53
combined_score													4.43E-42
homology													
erdos													

### Centrality analysis

**Fig. 5:**
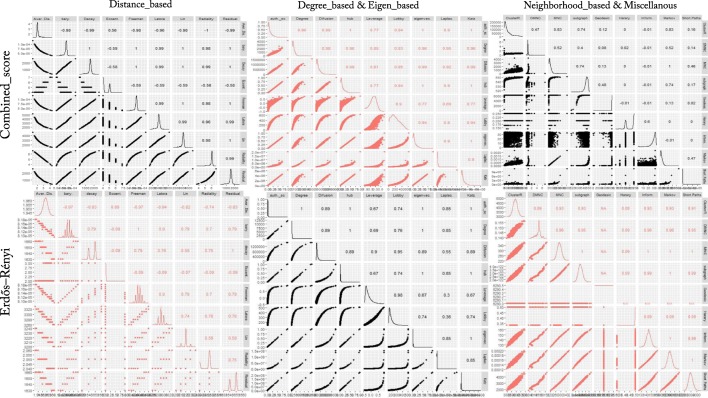
Pairwise scatterplot between the centrality measures. This figure contains combined-score PPIN and the null network. In this figure, the r Pearson correlation coefficients between centralities beside the centralities distribution were also presented in both networks. For better representation, red and black colors were used and the scatterplot was divided into three parts corresponding to Table 1 groups. For the scatterplot visualizations of all PPINs see Additional file 2

In the next step, the 27 centrality measures of nodes were computed in all 14 networks. The distribution and pairwise scatter plots of the computed measures were represented in Fig. 5 to point out pairwise relationship between them. (For the other PPINs see Additional file 3). The r correlation coefficients were also shown in this figure in which some of the centrality measures displayed a clear correlation and the others revealed a vast diversity among all five centrality classes. This diversity especially enriched in Distance-, Neighborhood-based and miscellaneous classes for combined-score PPIN compared with Erdos-Renyi network. Analogously, this special profile of centrality measures was repeated in all PPINs to some extent. Another remarkable distinction was the multimodality of distributions in the random network but not in real networks which was repeated for most of the Distance-based centrality measures. Furthermore, according to r correlation coefficients, the pairwise association of centrality measures were roughly higher in the null network than PPINs.

### Dimensionality reduction and clustering analysis

**Fig. 6:**
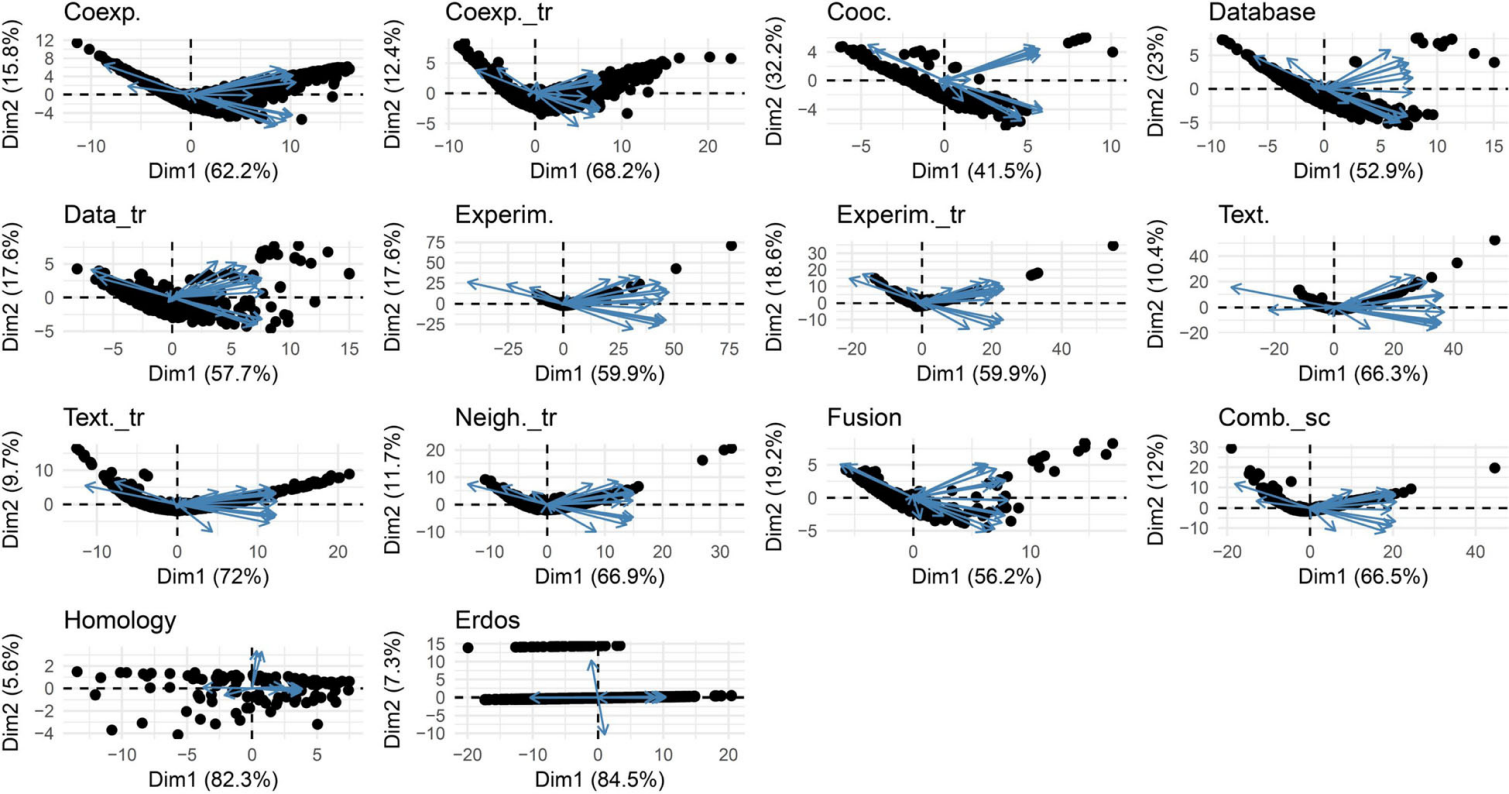
Biplot representation of the centrality measures in each network. The PCA plots were a projections of the multivariate data into the 2D space spanned by the first two principal components. In each plot, nodes were shown as points and centrality measures as vectors

**Fig. 7:**
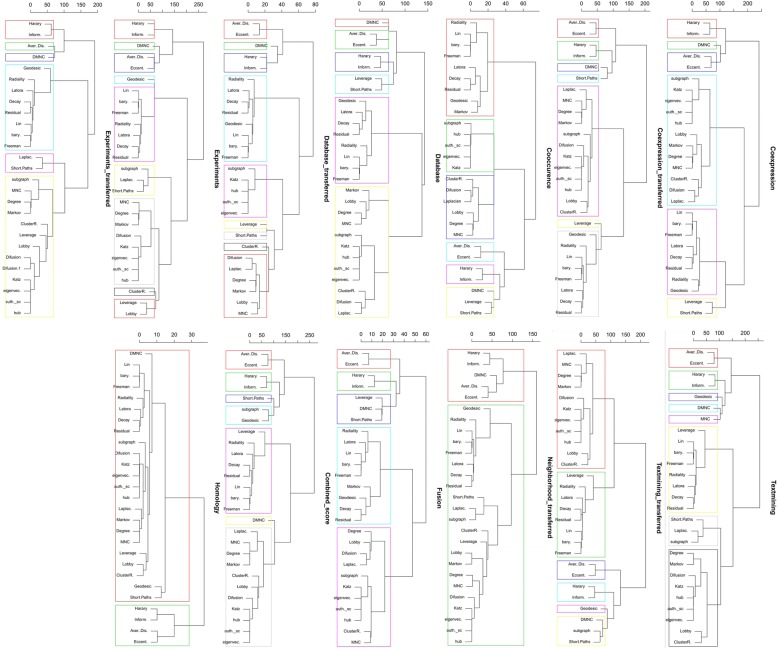
Clustering dendrograms. In each dendrogram, the colored boxes show ensued clusters of centrality measures in each PPIN based on a predefined distance threshold

**Fig. 8:**
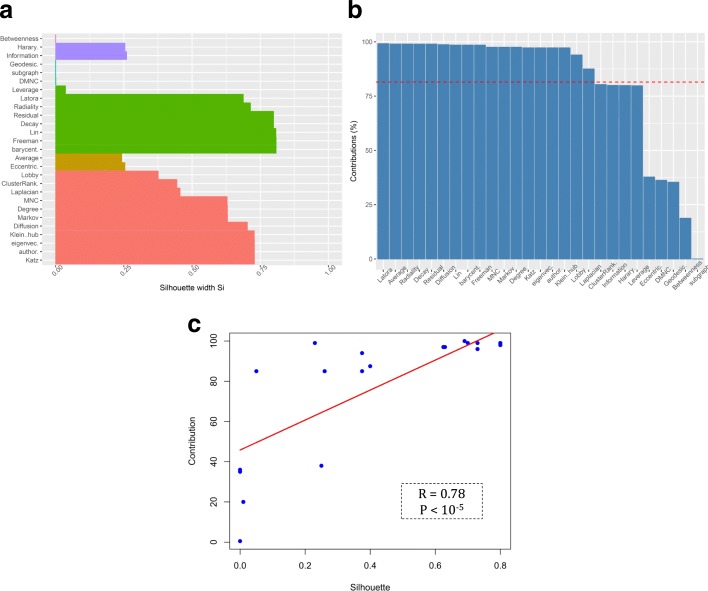
(**a**) Clustering silhouette plot of the combined-score PPIN. The colors represented the six clusters of the centrality measures in this PPIN. The average silhouette width was 0.49. (**b**) Contribution values of centrality measures according to their corresponding principal components in this PPIN. The number of principal components stand on the network architecture was equal to 3. The dashed line indicates the random threshold of contribution. (**c**) Line plot between silhouette and contribution values. The R value shown is the result of a regression coefficient analysis and the *p* value has been computed from Pearson’s correlation test

In the next step, PCA-based dimensionality reduction was used to reveal which centrality measures contain the most relevant information in order to effectively identify important or influential nodes in networks. As illustrated in Fig. 6, the profile of the distance to the center of the plot and their directions were mostly consonant except for the homology which was similar to the random network. The rank of contribution values of each centrality measure were shown in Table 4, depend on their corresponding principal components. The percentage of contribution of variables (i.e. centrality measures) in a given PC were computed as (variable.Cos2*100)/(total Cos2 of the component)). A similar profile of the contribution of centrality measures was observed among all biological networks even in homology network opposed to the random null network (See Additional file 4). On average, Latora closeness centrality was the major contributor of the principal components in PPINs. In contrast, other well-known centralities i.e. Betweenness and Eccentricity revealed a low contribution value in all PPINs. Analogous to the null network, their values were lower than random threshold depicted in Fig. 8 and Additional file 4. On the contrary, the Degree displayed moderate levels of contribution in all real networks whilst it was the fourth rank of random network contributors. Although the profile of contributions were similar, each PPIN exhibited a special fingerprint of the centrality ranking. Finally, by performing unsupervised categorization, we aimed to cluster centrality values computed in the networks. First, we performed a clustering tendency procedure. We found that the centrality values are clusterable in each network as all values in the Hopkins statistics were more than the cutoff (0.05). The results are shown in the first column of Table 5 and Additional file 5. Then, by calculating silhouette scores, three methods (i.e. hierarchical, k-means, and PAM) were evaluated in clustering the data sets (Additional files 6 and 7). The output of applying these algorithms and the corresponding number of clusters were also shown in Table 5 and Additional file 8. Using the hierarchical algorithm based on Ward’s method [72], the centrality measures were clustered in each PPINs (Fig. 7). Number of clusters, distance between centrality measures and centrality composition in all 13 PPINs indicated that each centrality ranks nodes within a given network distinctly. For a better comparison, we provided Table 6 containing pairwise Jaccard similarity indices for each network pair. The lowest values were related to the homology, neighborhood-transferred and co-occurrence PPINs while among these genome context prediction methods, fusion PPIN was more associated to the other networks. The high similarity between co-expression and co-expression-transferred was expected however the similar clusters of the database derived PPIN with both aforementioned PPINs and also combined-score with textmining-transferred are noteworthy.

**Table 4:**
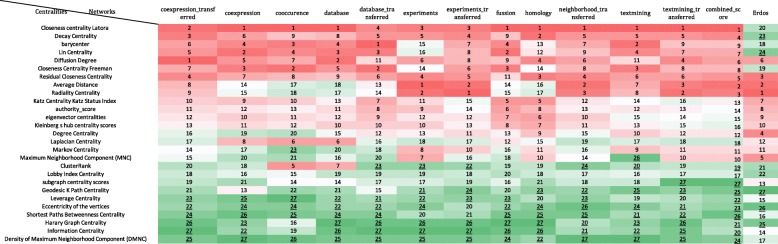
Ranking of the contribution values based on PCA for each network. The red to green highlighted cells represent the top to bottom ranked centrality measures in each network. The underlined ranking values are contribution values of the centrality measures which are below the random threshold

**Table 5:** Clustering information values for PPINs. The Hopkin’s statistics threshold for clusterability was 0.05

Network	Hopkins Statistic	Number of Clusters	Silhouette Average Value
Coexpression	0.25	6	0.36
Coexpression_transferred	0.21	7	0.33
Cooccurence	0.18	6	0.55
Database	0.24	6	0.33
Database_transferred	0.20	9	0.32
Experiments	0.21	9	0.31
Experiments_transferred	0.16	6	0.43
Textmining	0.24	8	0.28
Textmining_transferred	0.20	6	0.35
Neighborhood_transferred	0.26	2	0.39
Fussion	0.16	5	0.48
Combined_score	0.30	7	0.27
Homology	0.23	2	0.46

**Table 6:** Jaccard index coefficient values for PPINs. The values represent how similar the networks are, in terms of their clustering results. A value of 1 indicates an exact match while values equal to 0 show dissimilarity

	coexp.	coexp._tr	coocc.	comb.	dat._tr	dat.	exp.	exp._tr	fus.	hom.	nei._tr	tex.	tex._tr
coexpression		0.99	0.58	0.77	0.62	1.00	0.58	0.80	0.83	0.41	0.43	0.62	0.76
coexpression_transferred			0.57	0.78	0.63	0.99	0.58	0.81	0.82	0.40	0.43	0.62	0.77
cooccurence				0.47	0.75	0.58	0.44	0.50	0.73	0.29	0.30	0.43	0.48
combined_score					0.52	0.77	0.62	0.63	0.64	0.37	0.39	0.78	0.96
database_transferred						0.62	0.55	0.55	0.55	0.25	0.27	0.47	0.51
database							0.58	0.80	0.83	0.41	0.43	0.62	0.76
experiments								0.59	0.49	0.25	0.27	0.67	0.63
experiments_transferred									0.67	0.41	0.43	0.62	0.62
fussion										0.40	0.42	0.52	0.64
homology											0.91	0.30	0.37
neighborhood_transferred												0.32	0.39
textmining													0.78
textmining_transferred													

## Discussion

Interestingly, silhouette scores of centrality measures were closely related to corresponding contribution value of the measures (Fig. 8). Where there was a high silhouette value, a high contribution value was observed, however, a high contribution value did not always mean a high silhouette value. The relationship between the silhouette scores and contribution values of each centrality measure was also examined by regression analysis. Latora closeness, Radiality, Residual, Decay, Lin, Leverage, Freeman closeness and Barycenter centrality measures were present together in the same cluster where the corresponding silhouette scores were all at a high level except the Leverage’s score (Fig. 8a). The average silhouette score was around 0.66 in this cluster. On the other hand, the Leverage’s contribution value was below the threshold line and placed in the group with the least amount of contribution (Fig. 8b). The centrality measures namely Lobby index, ClusterRank, Laplacian, MNC, Degree, Markov, Diffusion degree, Kleinberg’s hub, Eigen vector, Authority score, Katz group together where the mean of their silhouette scores (i.e. 0.61) was higher than the overall average and in the same way, their corresponding contribution values were high, too. On the other hand, we observed that Shortest path Betweenness (which was in a separated cluster) and Geodesic k path, Subgraph and DMNC (which are all in one cluster) showed the low silhouette value mean (i.e. 0.03) much lower than the average. In all other PPINs, the same relationship between silhouette scores and contribution values was observed as shown in Additional files 4 and 7.

Our results demonstrated that a unique profile of centrality measures including Latora closeness, Barycenter, Diffusion degree, Freeman closeness, Residual, Average distance, Radiality centralities, was the most significant indicator in ranking PPIN nodes. We inferred that the rationale and logic of network reconstruction dictates which centrality measures should be chosen. Also, we demonstrated the relationship between contribution value derived from PCA and silhouette width as a cluster validity index. Regarding to the robustness issue, we first reasserted that the architecture and global properties of a network impact on the centrality analysis results [73–75]. Therefore, the center of a network would be different, depending on the network’s inherent topology. In other words, we addressed this issue whether a given centrality measure has enough information via-a-vis and it demonstrates a same behavior in some other networks.

## Conclusion

Network-based methods have been introduced as an emergent approach for simplification, reconstruction, analysis, and comprehension of complex behavior in biological systems. Network-based ranking methods (i.e. centrality analysis) have been found widespread use for predicting essential proteins, proposing drug targets candidates in treatment of cancer, biomarker discovery, human disease genes identification and creation a cell with the minimal genome [76]. However, there is no consensus pipeline for centrality analysis regarding aforementioned applications among network analysts.

In this study, we worked on yeast PPINs which were built using 13 evidence channels in the STRING database. Subsequently, 27 centrality measures were used for the prioritization of the nodes in all PPINs. We illustrated that data reduction and low-dimensional projection help to extract relevant features (i.e. centrality measures) and corresponding relationships. Thus, to quantify connectivity in biological networks, we recommend that before arbitrary picking centrality measures to pinpoint important nodes, PCA (as an example of data projection methods) conduce how to use these measures. In the other word, the analysis of principal components clarifies which measures have the highest contribution values, i.e., which measures comprise much more information about centrality. Freshly, the application of these approach for discovering essential proteins was assayed in a polypharmacology study to prevent epithelial-mesenchymal transition in cancer [77].

### Basic definitions

- **Giant component of a graph** defines the largest connected component of a graph in which there is a path between each pair of nodes [78].
- **Network density** is a representation of the number of interactions to the number of possible interactions among a given network [79].
- **Network centralization** refers to a topological spectrum from star to grid topologies (where each node has a same number of links) of a graph varies from 1 to 0 [79].
- The **network heterogeneity** measure describes as the coefficient of variation of connectivity distribution. A high heterogeneous network implies that the network is exhibited approximate scale-free topology [79, 80].
- The **clustering coefficient** of a node is the number of triangles (3-loops) that pass through it, relative to the maximum number of 3-loops that could pass through the node. The network clustering coefficient defines as the mean of the clustering coefficients for all nodes in the network [81, 82].
- **Influential nodes** which is generally used in social networks analysis point as nodes with good spreading properties in networks [83]. Different centrality measures are used to find influential nodes.
- **Centrality-lethality rule** explains nodes with high centrality values in which maintain the integrity of the network structure, are more related to the survival of the biological system [84].
- The **silhouette criterion** defines how similar a centrality is to its own cluster compared to other clusters. It ranges from − 1 to 1, where a high value infers that the centrality is well matched to its own cluster and poorly matched to neighboring clusters. If most centralities have a high value, then the clustering configuration is proper. If they have low or negative values, then the clustering configuration may have too many or too few clusters [5, 85].

In order to see definitions of all used centrality measures, see http://www.centiserver.org.

## Additional files

Additional file 1: Evidence channel dataset. The contents of 13 evidence channels illustrating the yeast PPIN from STRING database. (downloaded in 3rd Sep 2016) are provided. (TXT 28629 kb)

Additional file 2: Fitted power law distribution. The Degree distribution of each network has been compared to the power law distribution in order to visualize the scale free property in the structure of each network. (PDF 203 kb)

Additional file 3: Scatterplots between groups of centralities. Each panel indicates scatterplots between centralities groups of two networks. (PPTX 1963 kb)

Additional file 4: Contribution values of centralities in each network. These values were computed based on the principal components. The red line shows the threshold used for identifying effective centralities. (PDF 441 kb)

Additional file 5: Visual assessment of cluster tendency plots. Each rectangular represents the clusters of the calculated results of the centrality measures. (PDF 313 kb)

Additional file 6: Clustering properties results. These properties include connectivity, Dunn and Silhouette scores. These scores suggest the sufficient clustering method by a specific number of clusters. (DOCX 16 kb)

Additional file 7: Clusters silhouette plots. Each color represents a cluster and each bar with specific color indicates a centrality. (PDF 417 kb)

Additional file 8: Optimal number of clusters. The suitable number of clusters for hierarchical clustering method was computed using the average silhouette values. (PDF 321 kb)

## Abbreviations

DMNC: Density of Maximum Neighborhood Component
MNC: Maximum Neighborhood Component
PAM: Partitioning Around Medoids
PCA: Principal Component Analysis
PPIN: Protein-protein interaction network
VAT: Visual Assessment of cluster Tendency
