# Supplementary material for: A systematic survey of centrality measures for protein-protein interaction networks
Source: BMC Syst Biol. 2018 Jul 31;12:80. doi: 10.1186/s12918-018-0598-2 (PMC6069823; doi:10.1186/s12918-018-0598-2)
Supplement: Supplementary file 2 — Fitted power law distribution. The Degree distribution of each network has been compared to the power law distribution in order to visualize the scale free property in the structure of each network. (PDF 203 kb) [file 12918_2018_598_MOESM2_ESM.pdf]

“Coexpression”

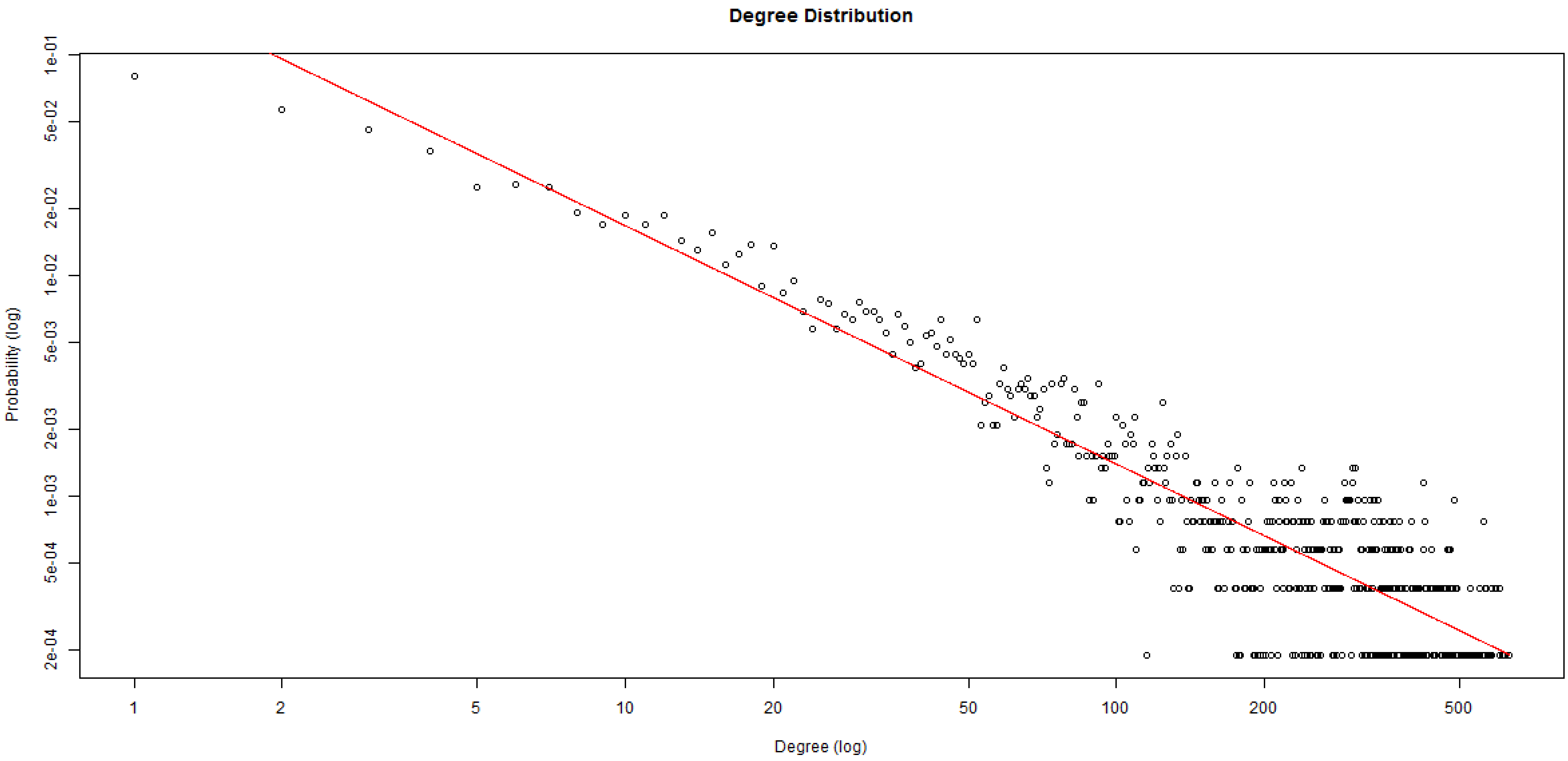

“Coexpression_transferred”

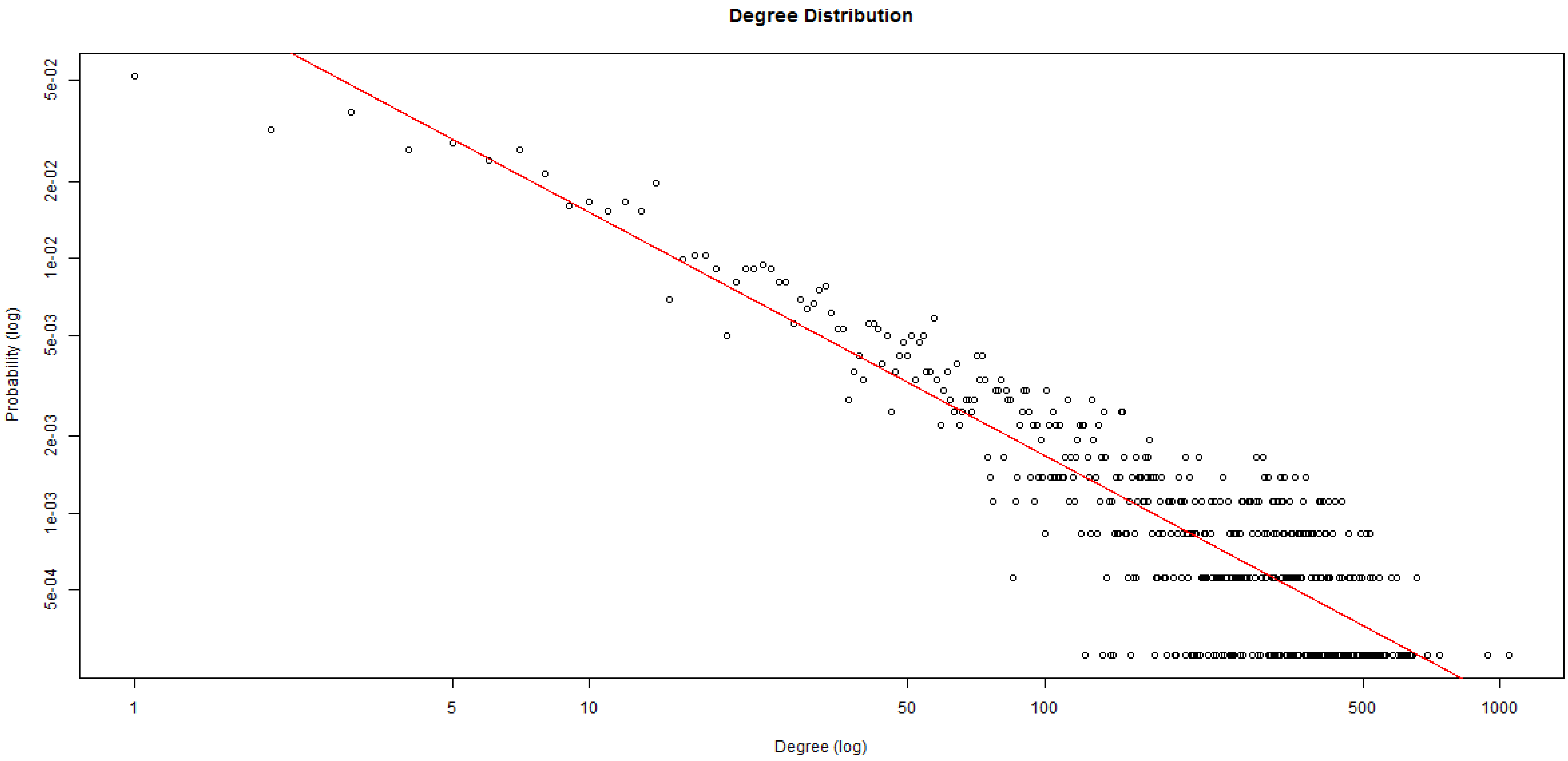

“Cooccurence”

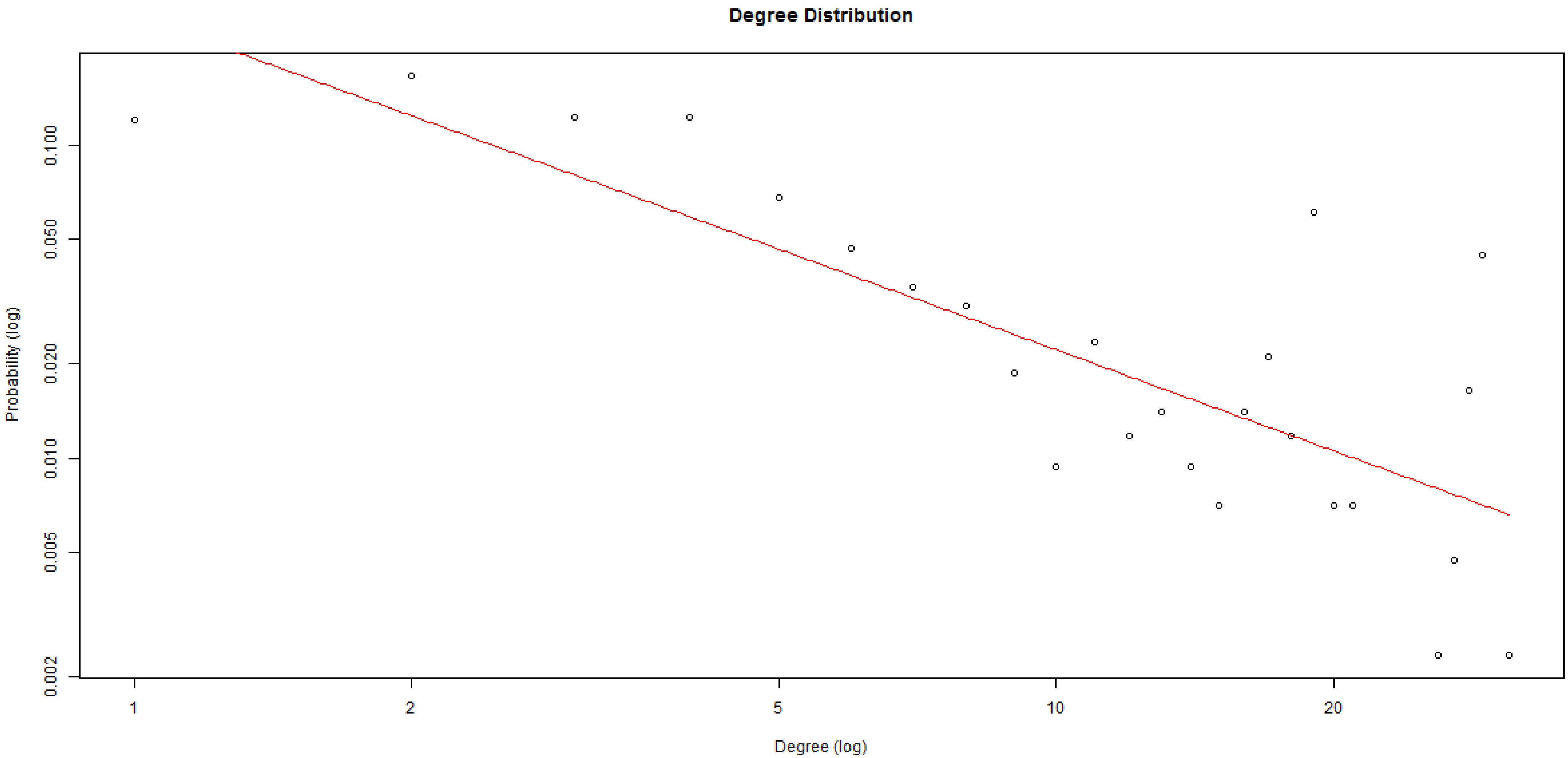

“Database”

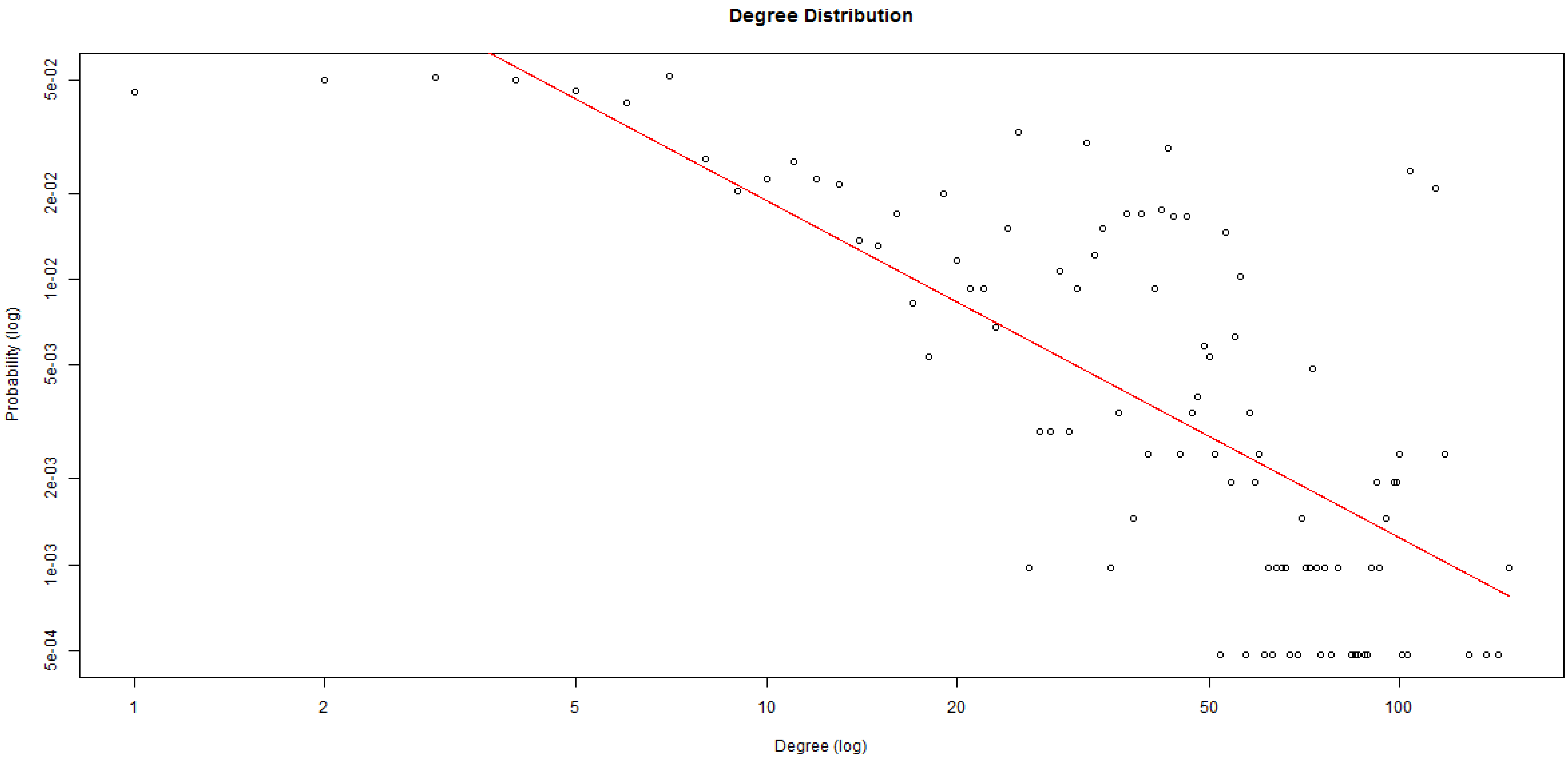
Degree Distribution
Probability (log)
5e-02
2e-02
1e-02
5e-03
2e-03
1e-03
5e-04
1
2
5
10
20
50
100
Degree (log)

“Database_transferred”

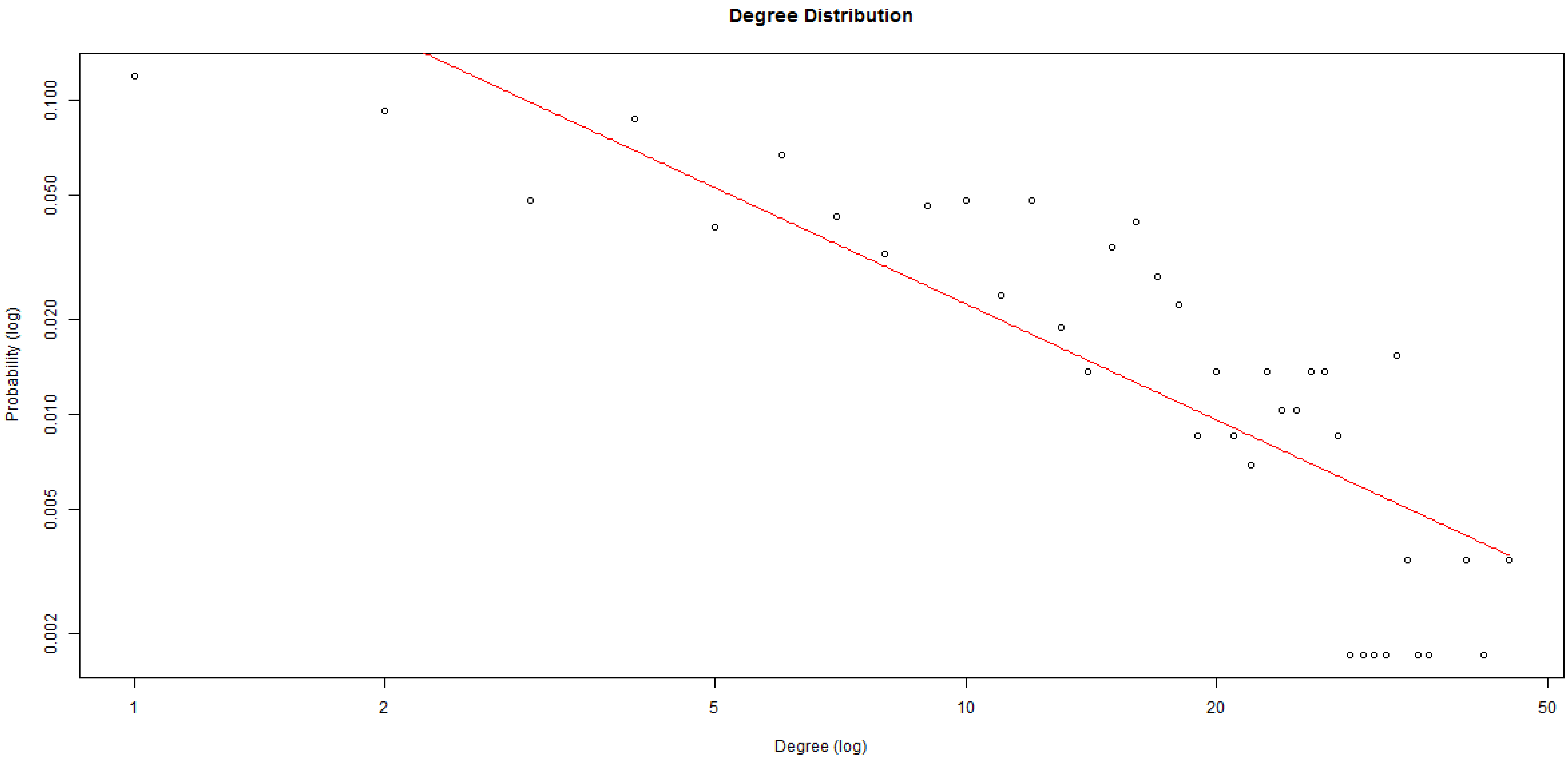

“Experiments”

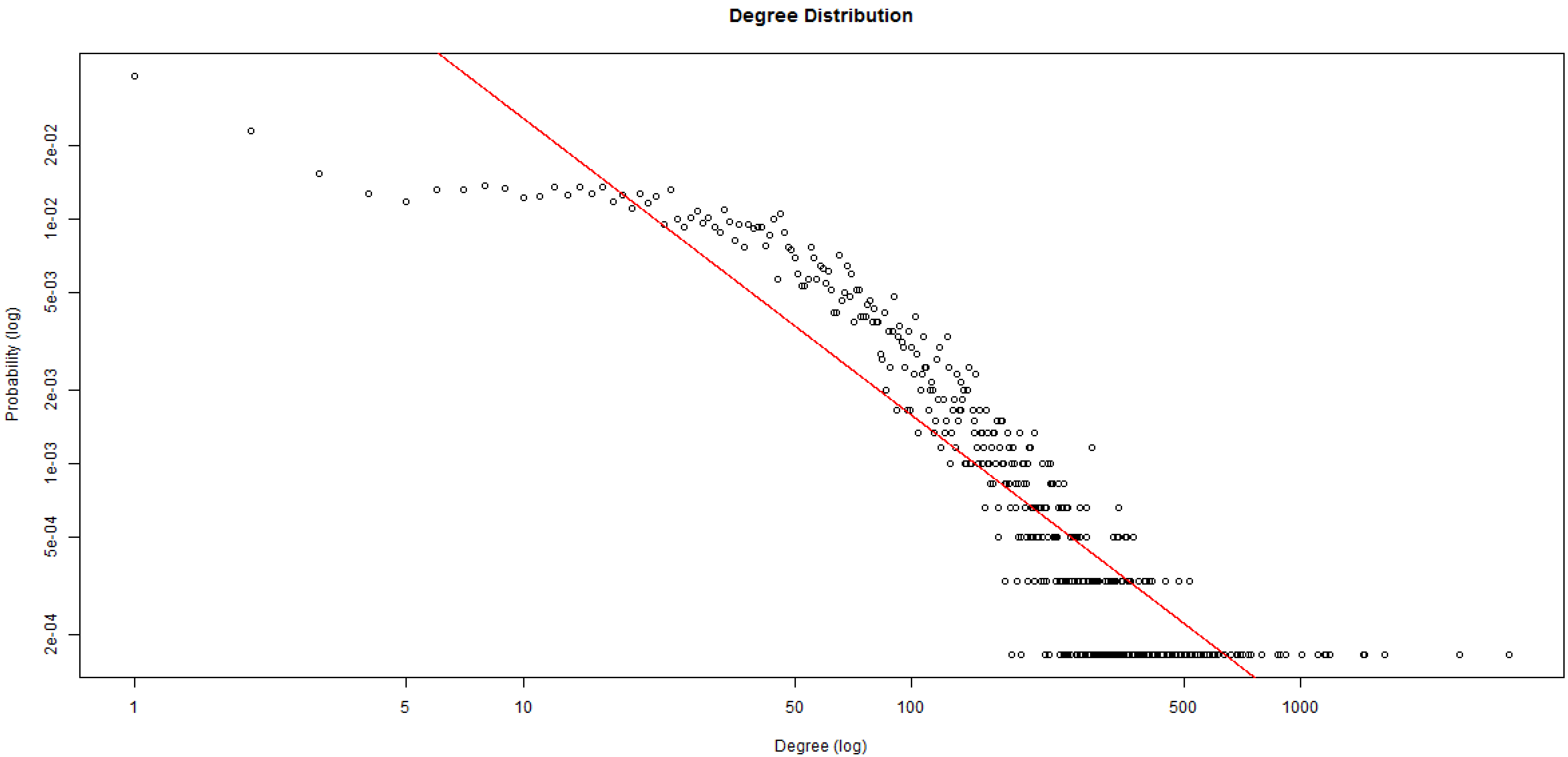

“Experiments_transferred”

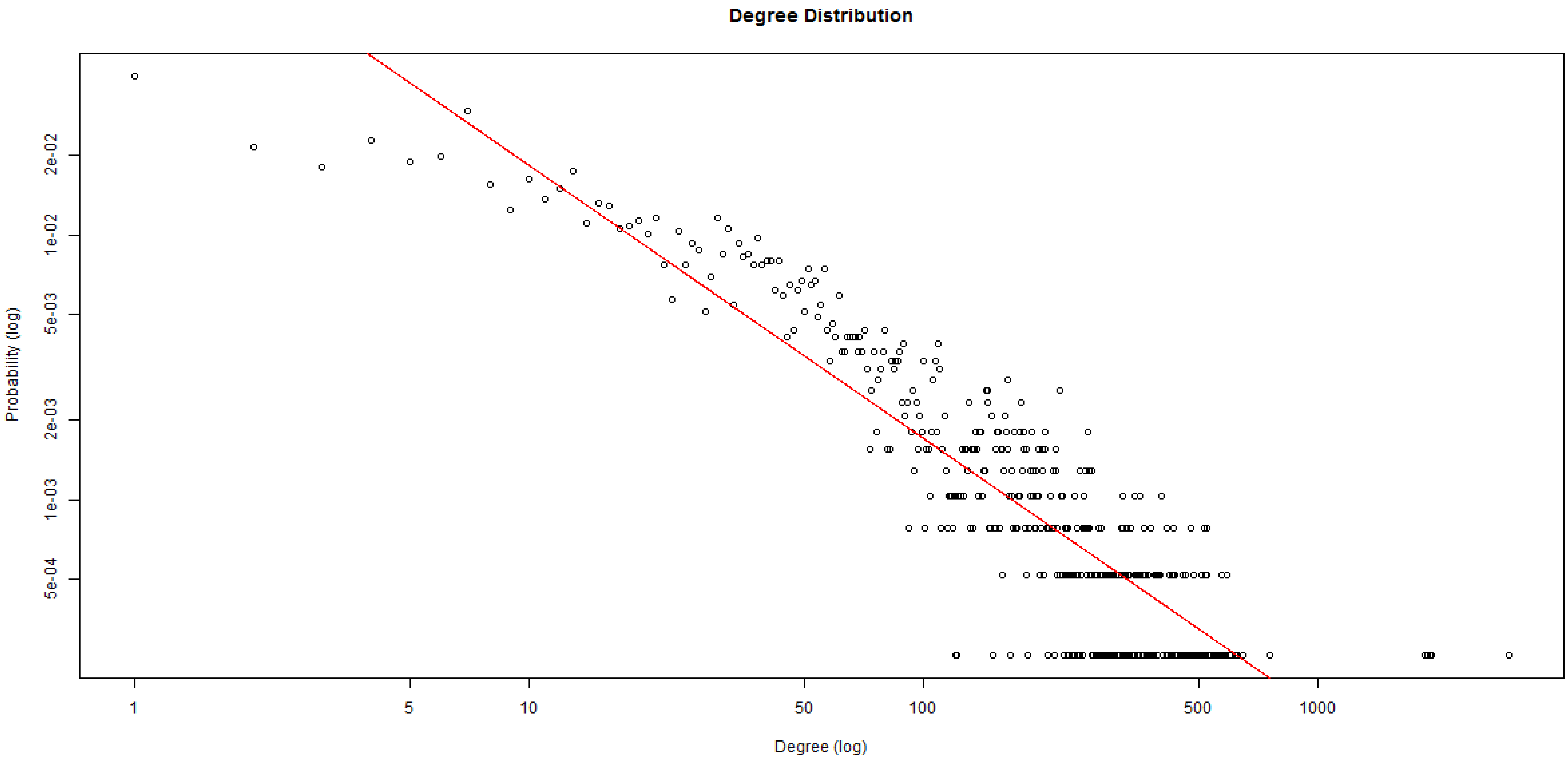

“Textmining”

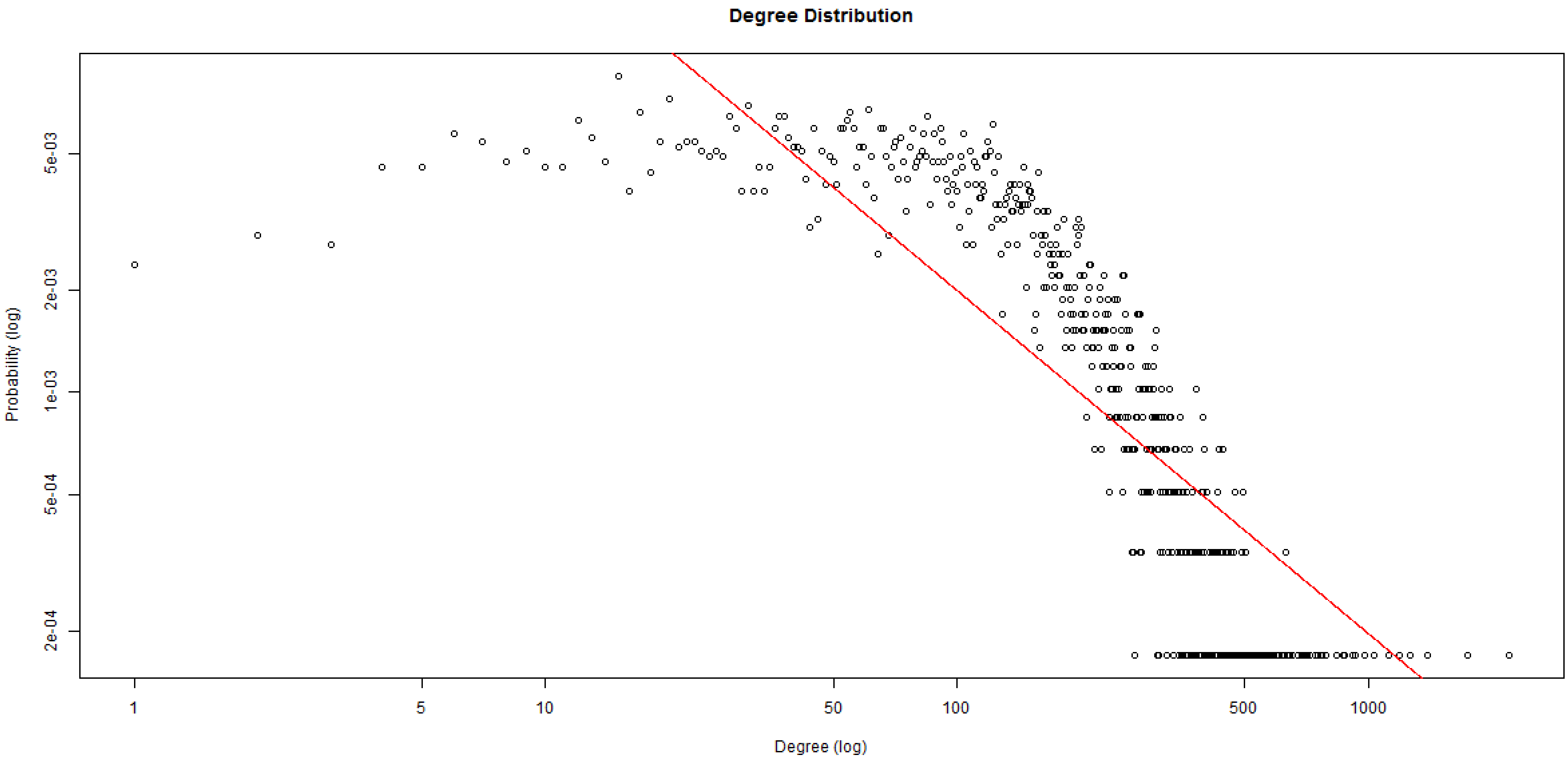
Degree Distribution
Probability (log)
5e-03
2e-03
1e-03
5e-04
2e-04
1
5
10
50
100
500
1000
Degree (log)

“Textmining_transferred”

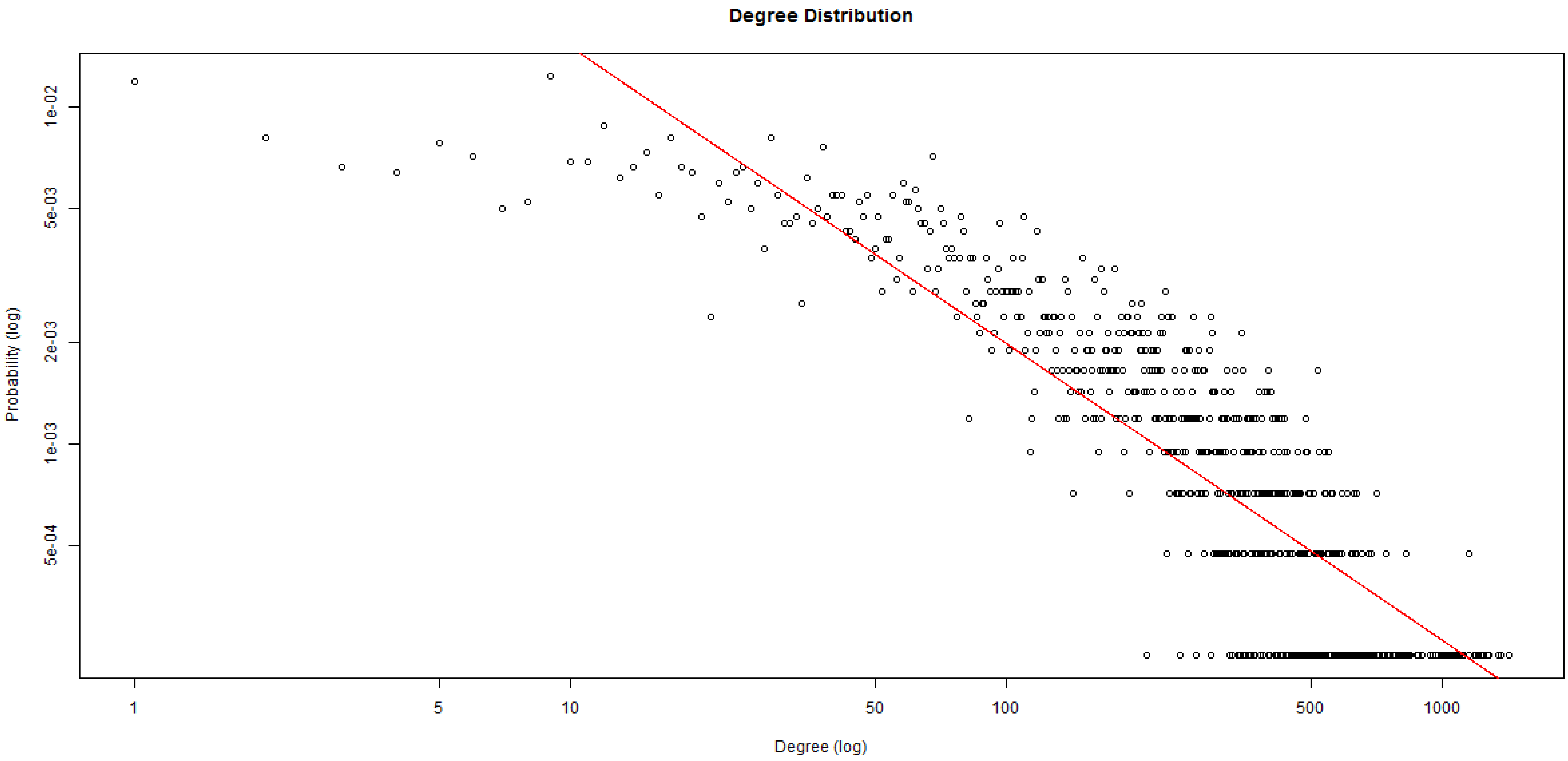

“Neighborhood_transferred”

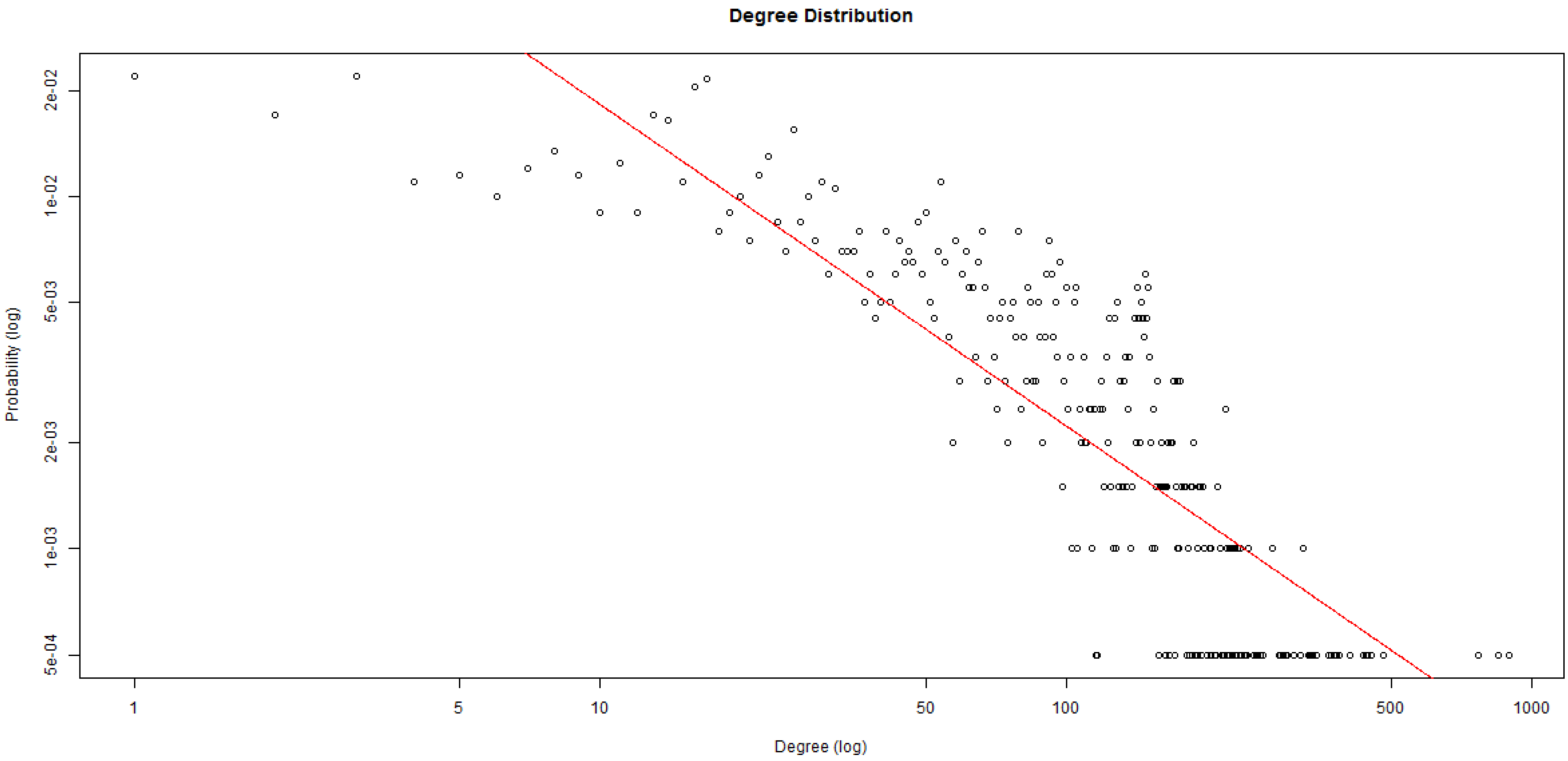

“Fusion”

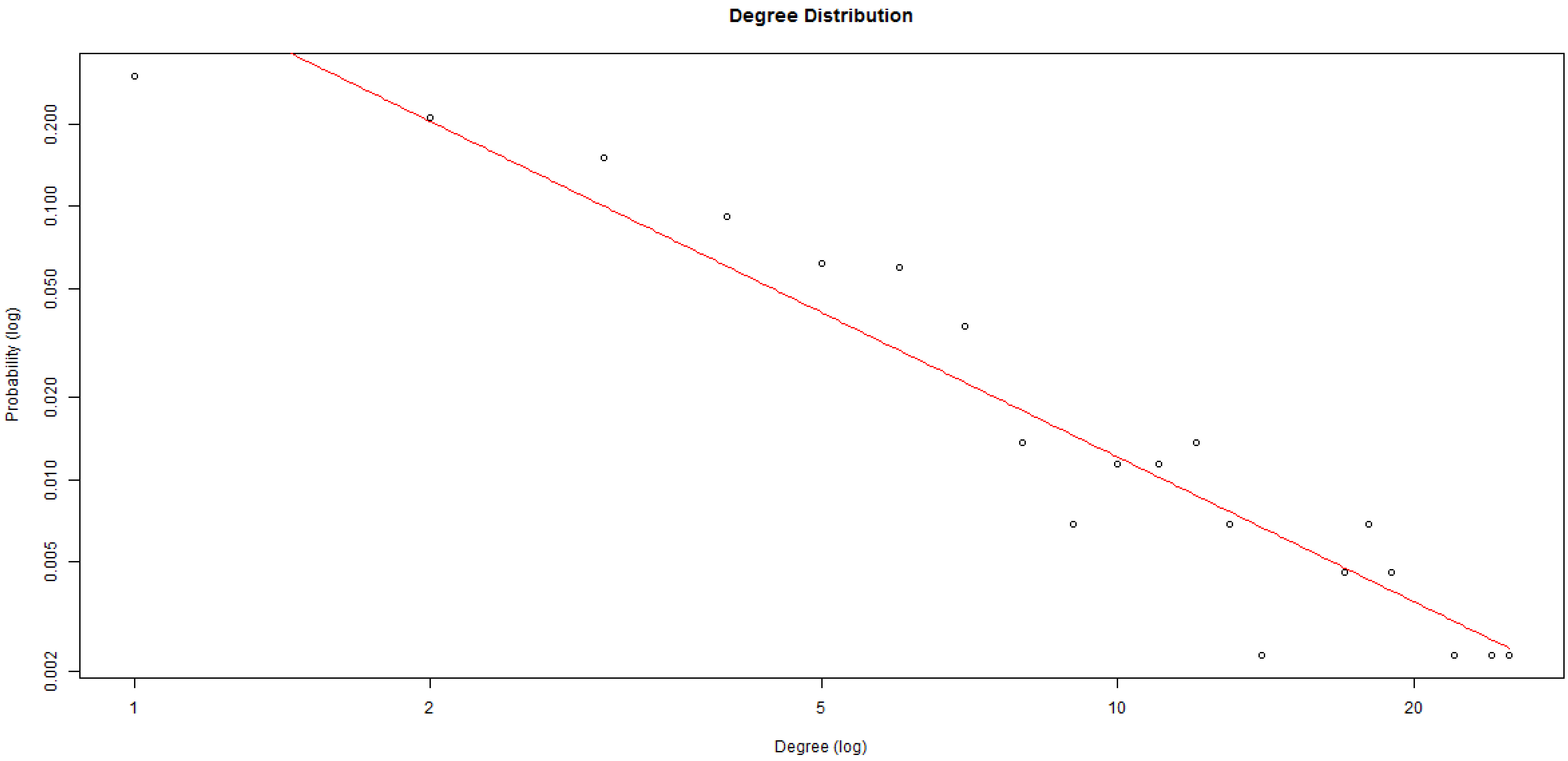

Degree Distribution
0.200
0.100
0.050
0.020
0.010
0.005
0.002
Probability (log)
1
2
5
10
20
Degree (log)

“Combined_score”

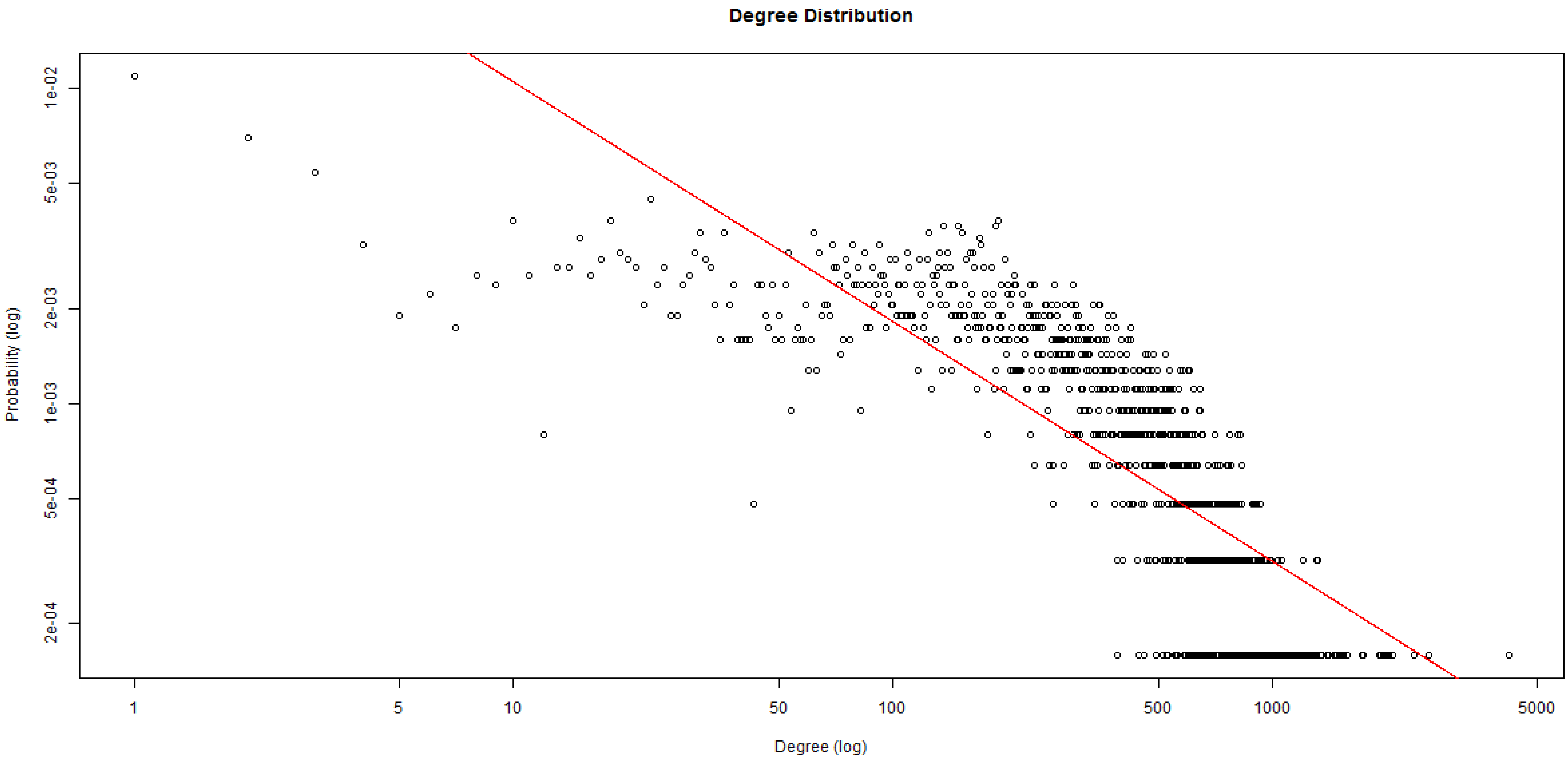

“Homology”

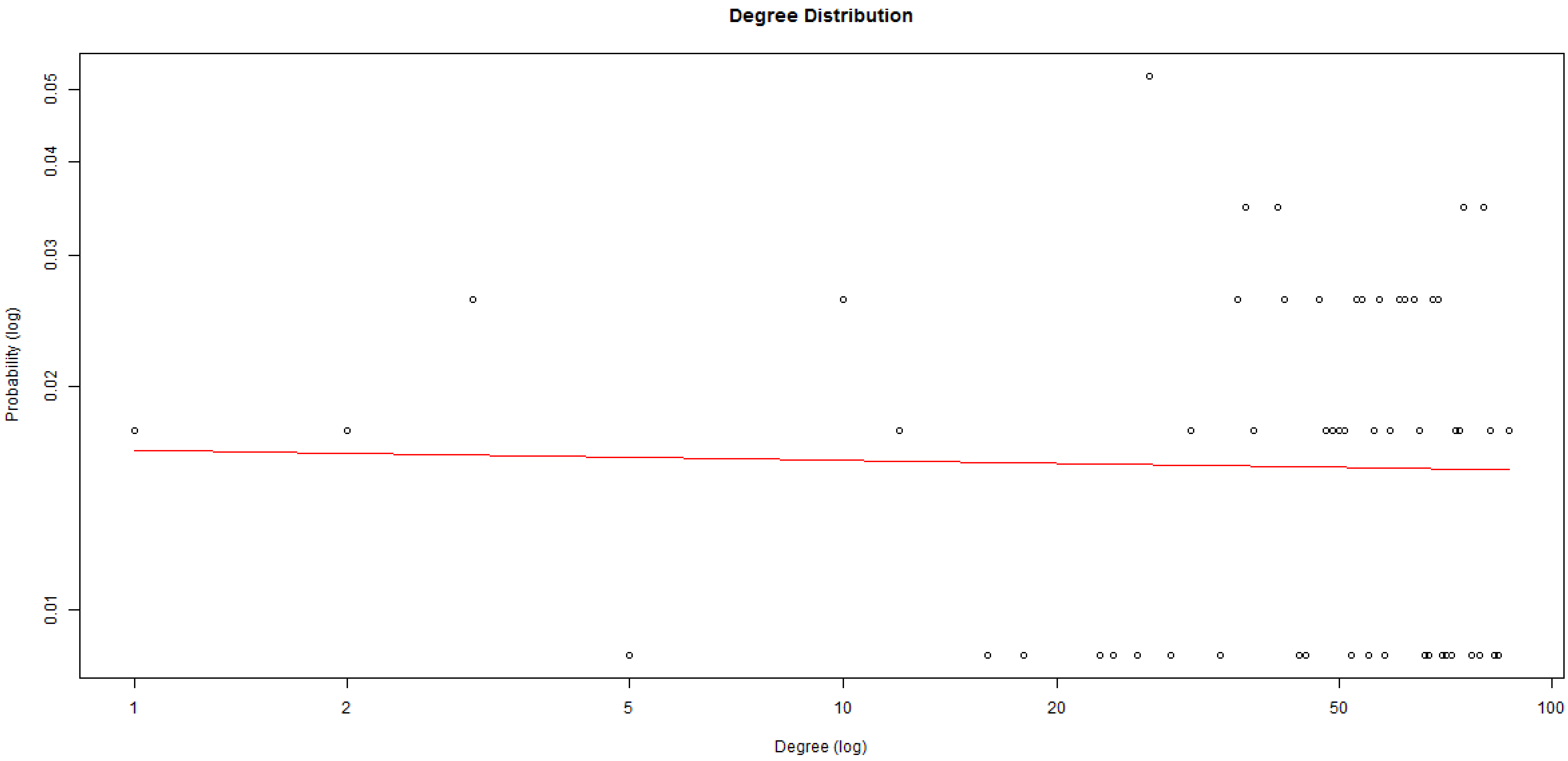

Degree Distribution
Probability (log)
0.05
0.04
0.03
0.02
0.01
1
2
5
10
20
50
100
Degree (log)

“Erdős–Rényi”

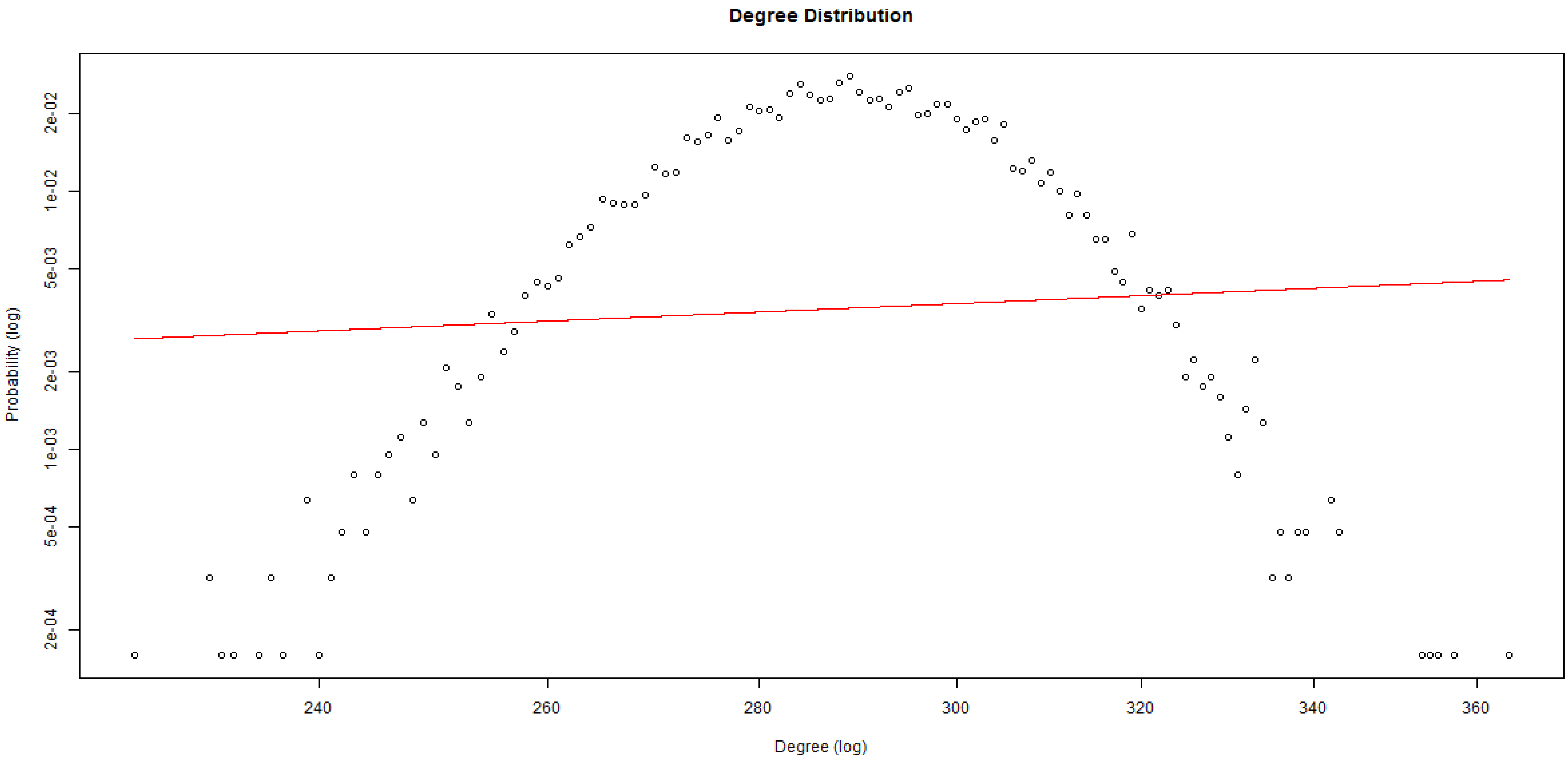
Degree Distribution
Probability (log)
2e-02
1e-02
5e-03
2e-03
1e-03
5e-04
2e-04
240
260
280
300
320
340
360
Degree (log)
