# Supplementary material for: A systematic survey of centrality measures for protein-protein interaction networks
Source: BMC Syst Biol. 2018 Jul 31;12:80. doi: 10.1186/s12918-018-0598-2 (PMC6069823; doi:10.1186/s12918-018-0598-2)
Supplement: Supplementary file 3 — Scatterplots between groups of centralities. Each panel indicates scatterplots between centralities groups of two networks. (PPTX 1963 kb) [file 12918_2018_598_MOESM3_ESM.pptx]

## Slide 1
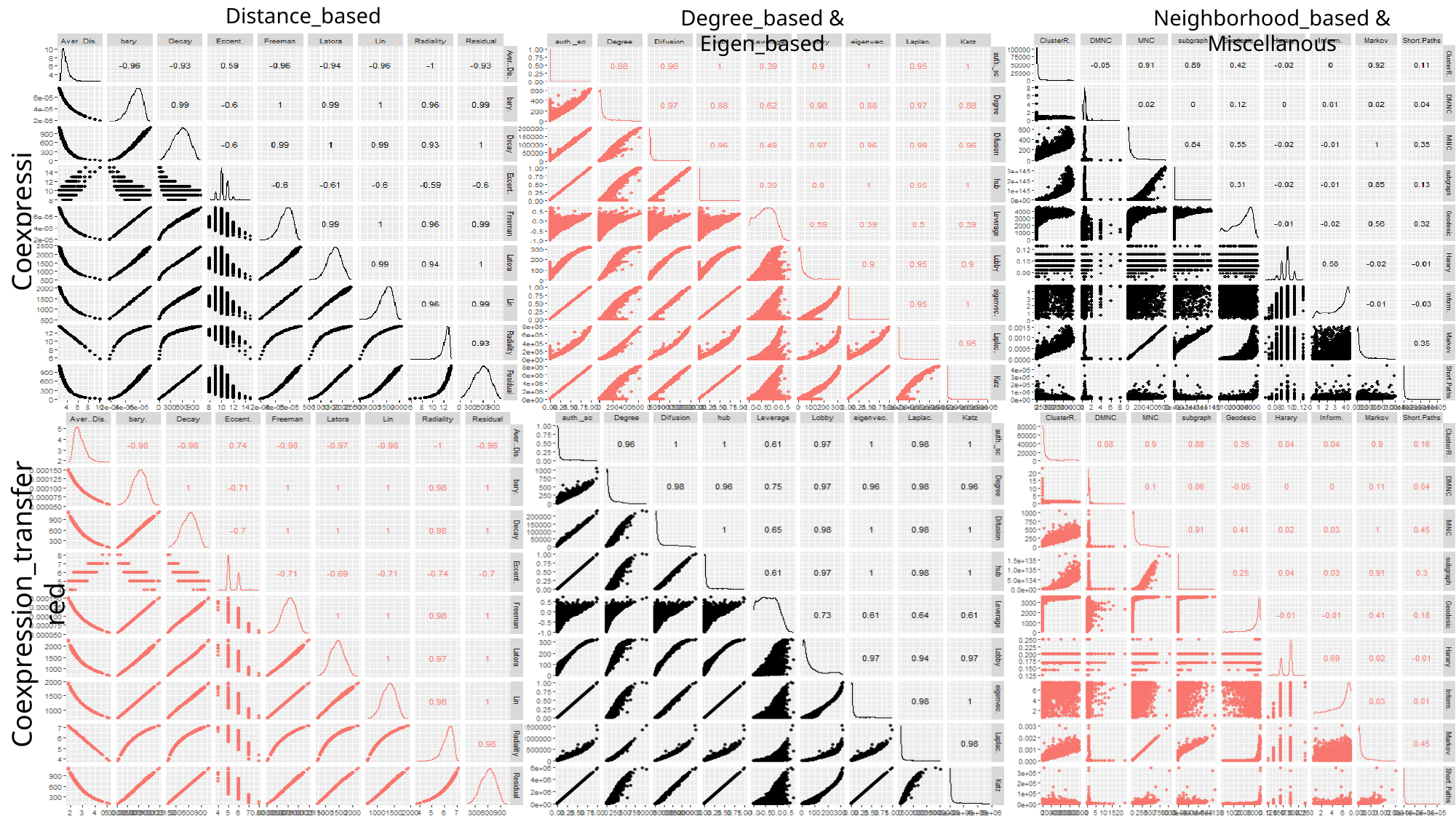

Distance_based
Degree_based & Eigen_based
Neighborhood_based & Miscellanous
Coexpression
Coexpression_transferred

## Slide 2
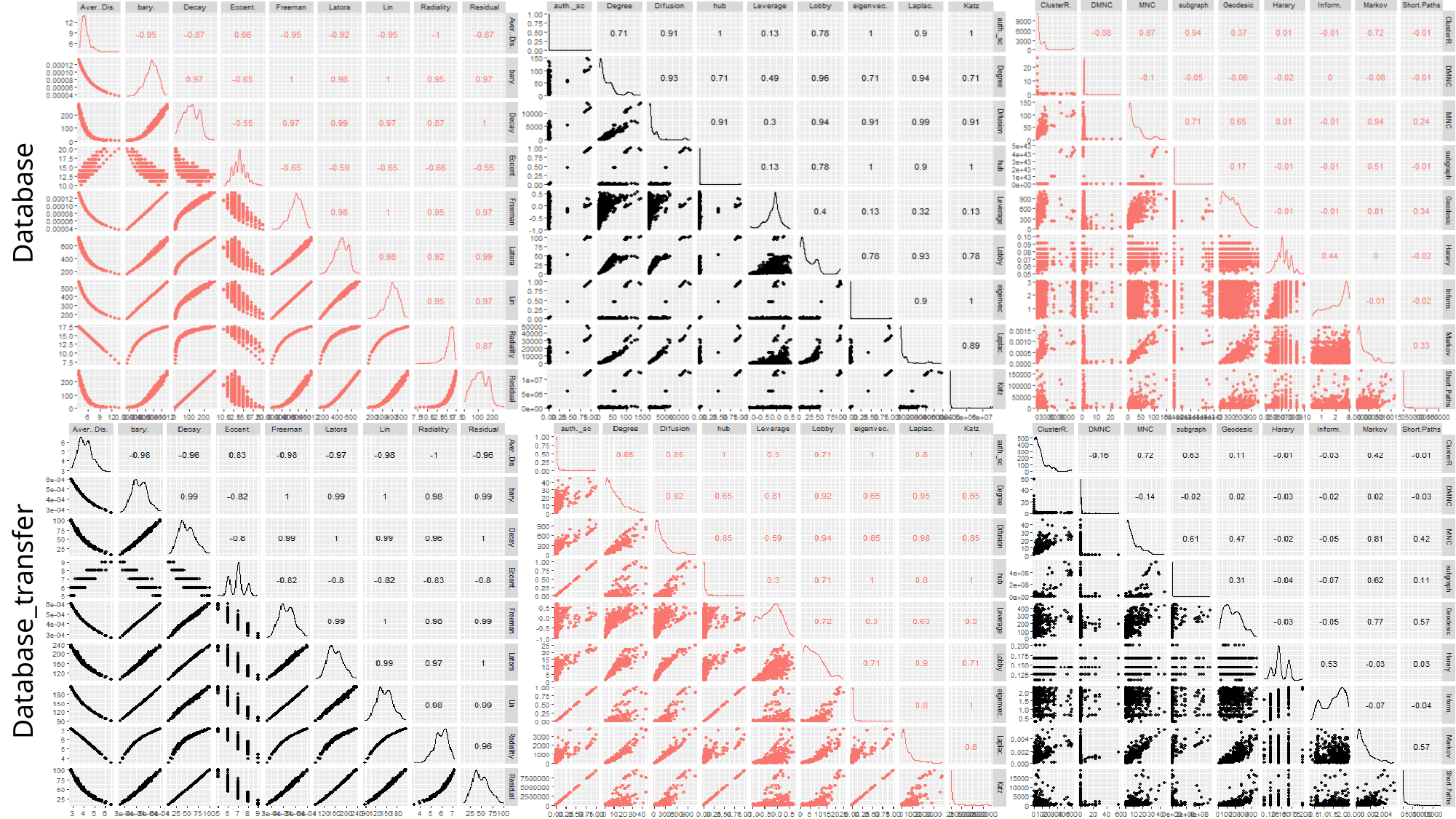

Database
Database_transferred

## Slide 3
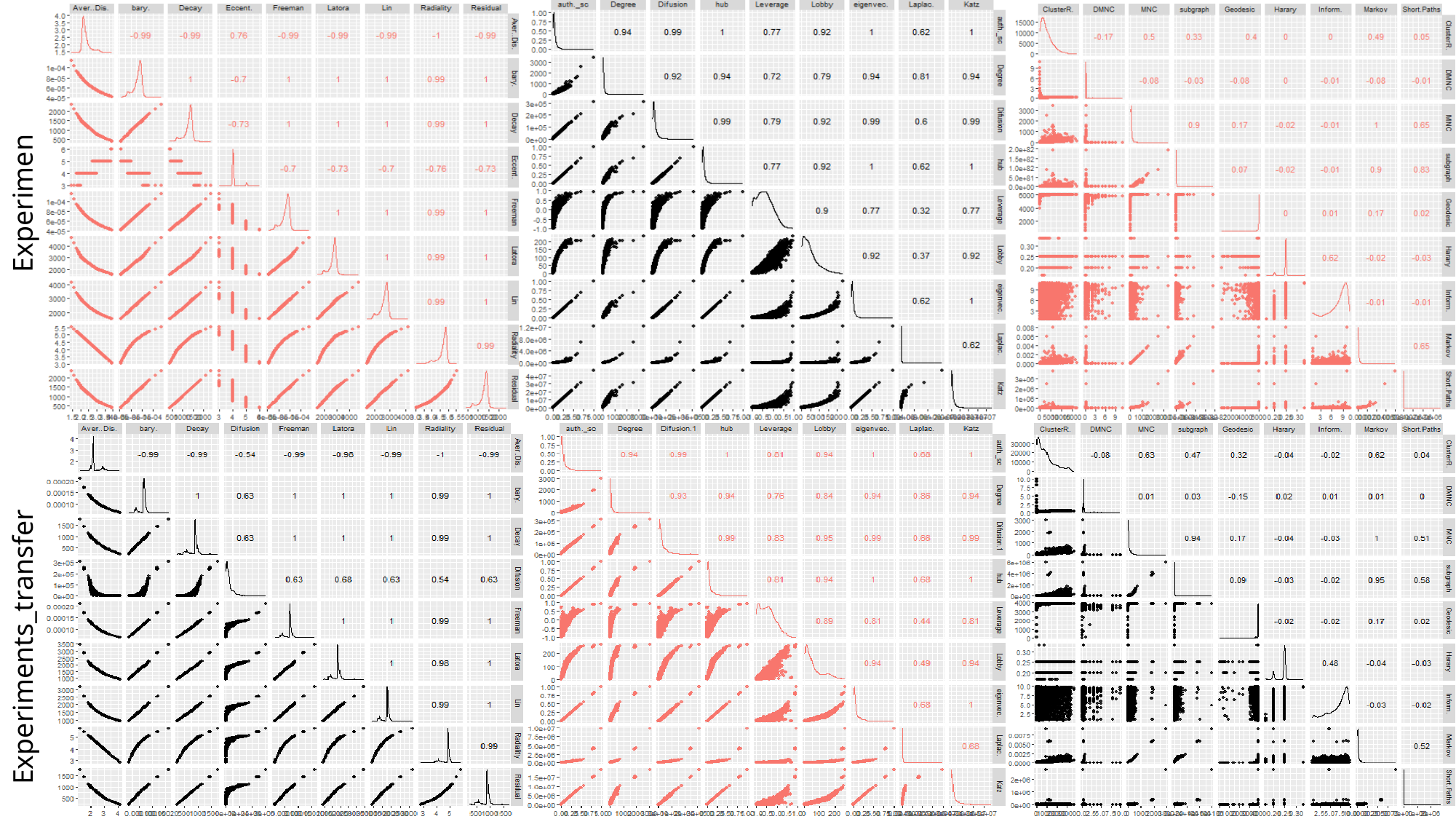

Experiments
Experiments_transferred

## Slide 4
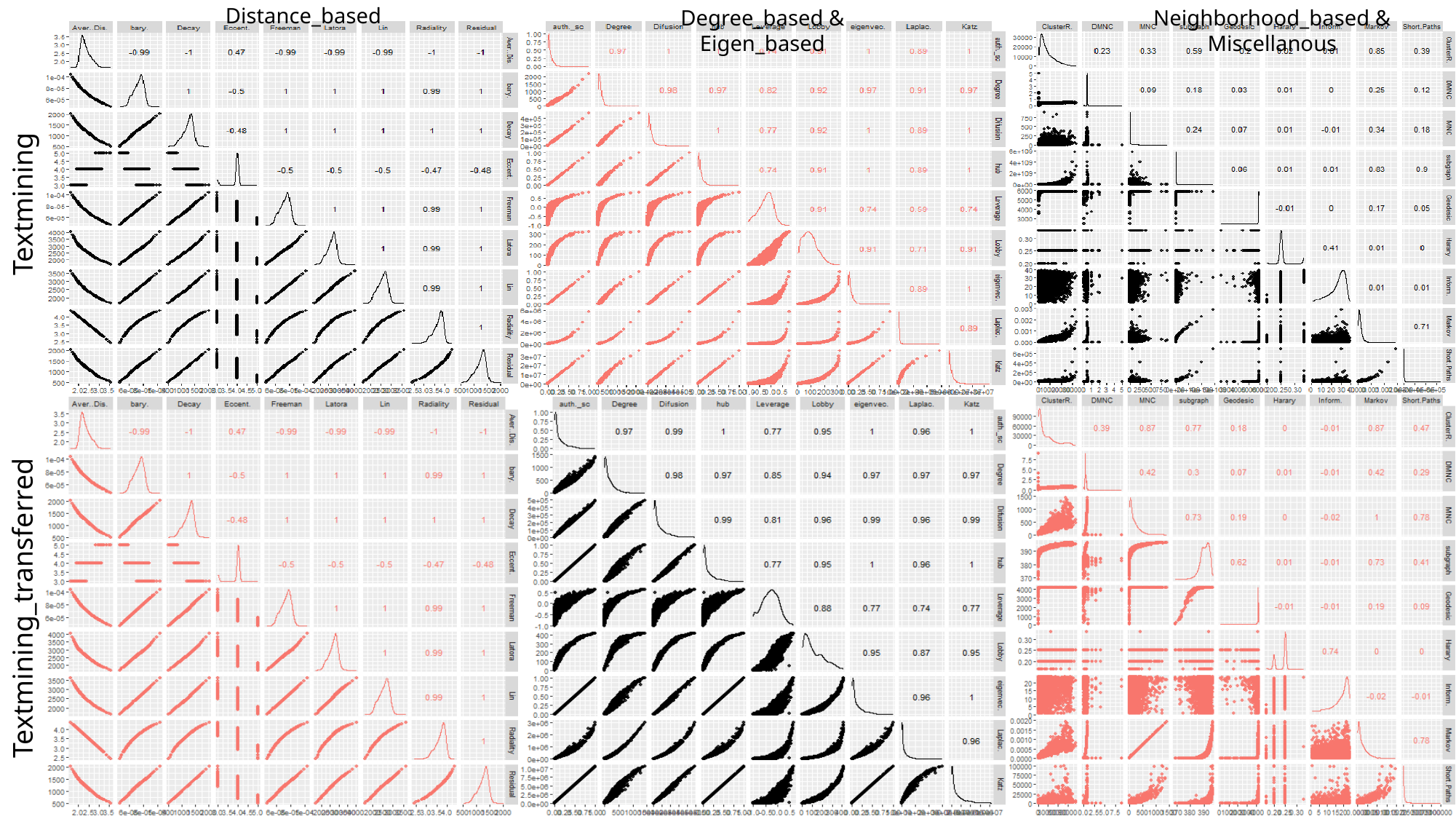

Distance_based
Degree_based & Eigen_based
Neighborhood_based & Miscellanous
Textmining
Textmining_transferred

## Slide 5
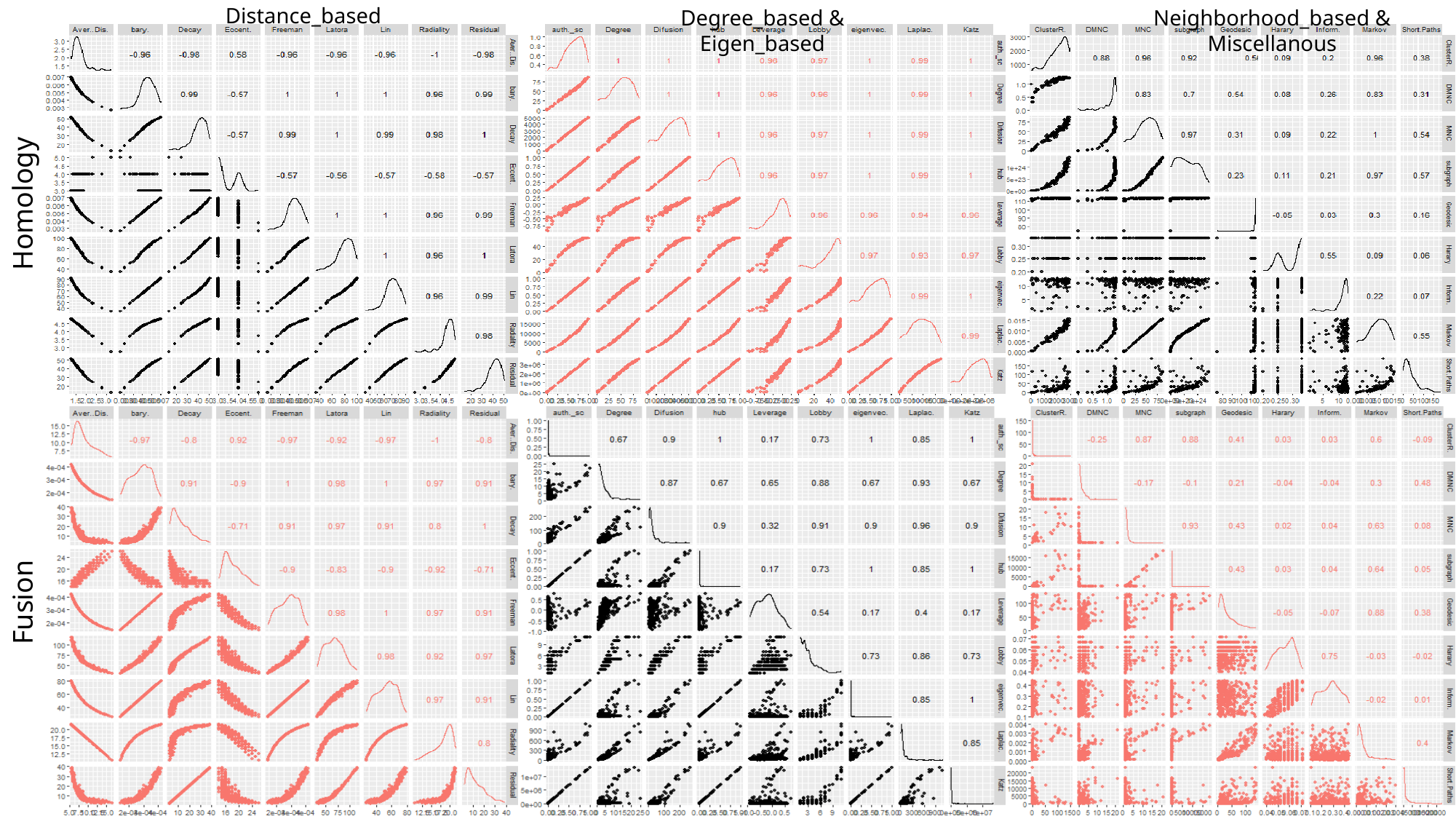

Distance_based
Degree_based & Eigen_based
Neighborhood_based & Miscellanous
Homology
Fusion

## Slide 6
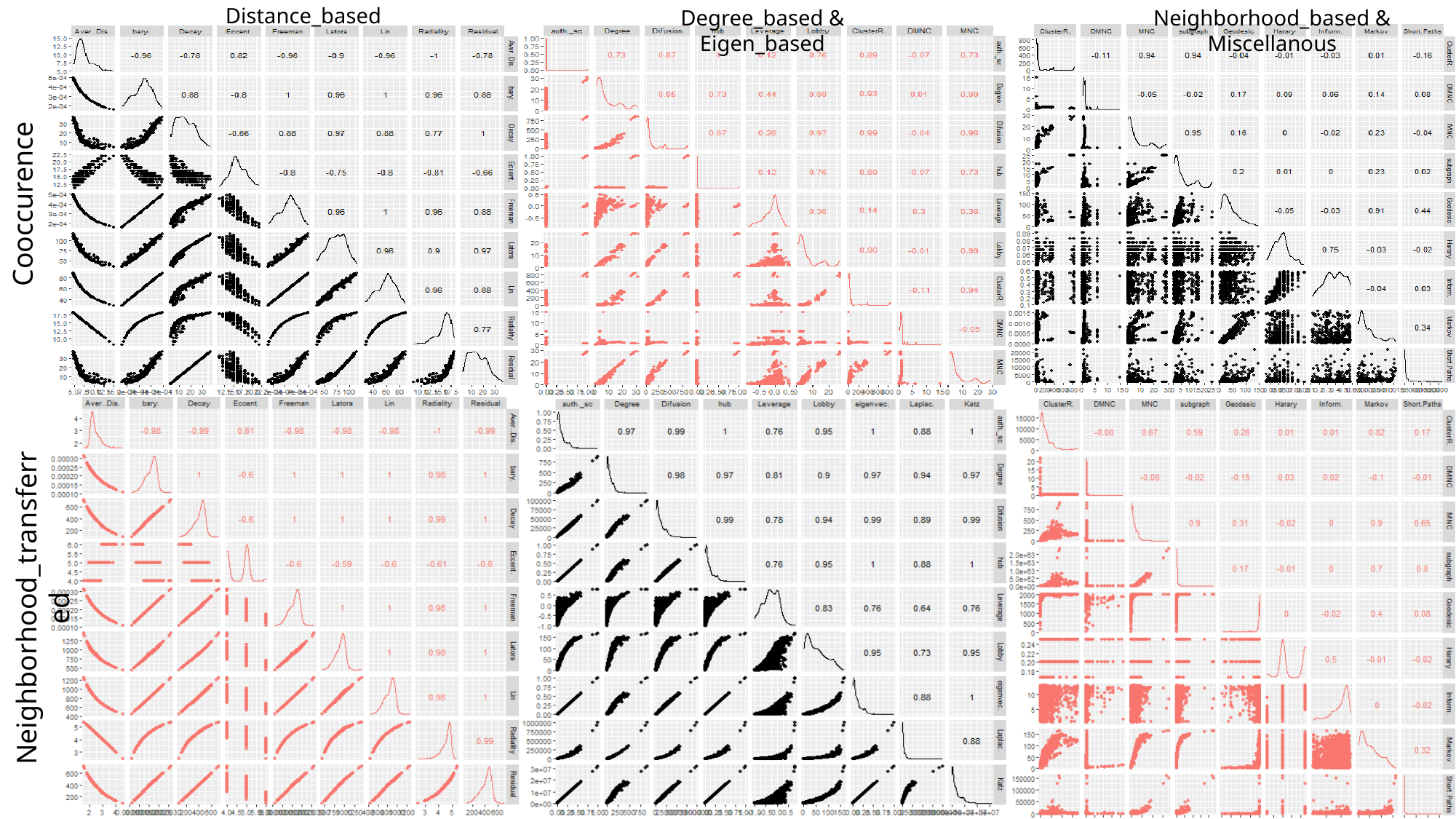

Distance_based
Degree_based & Eigen_based
Neighborhood_based & Miscellanous
Cooccurence
Neighborhood_transferred

## Slide 7
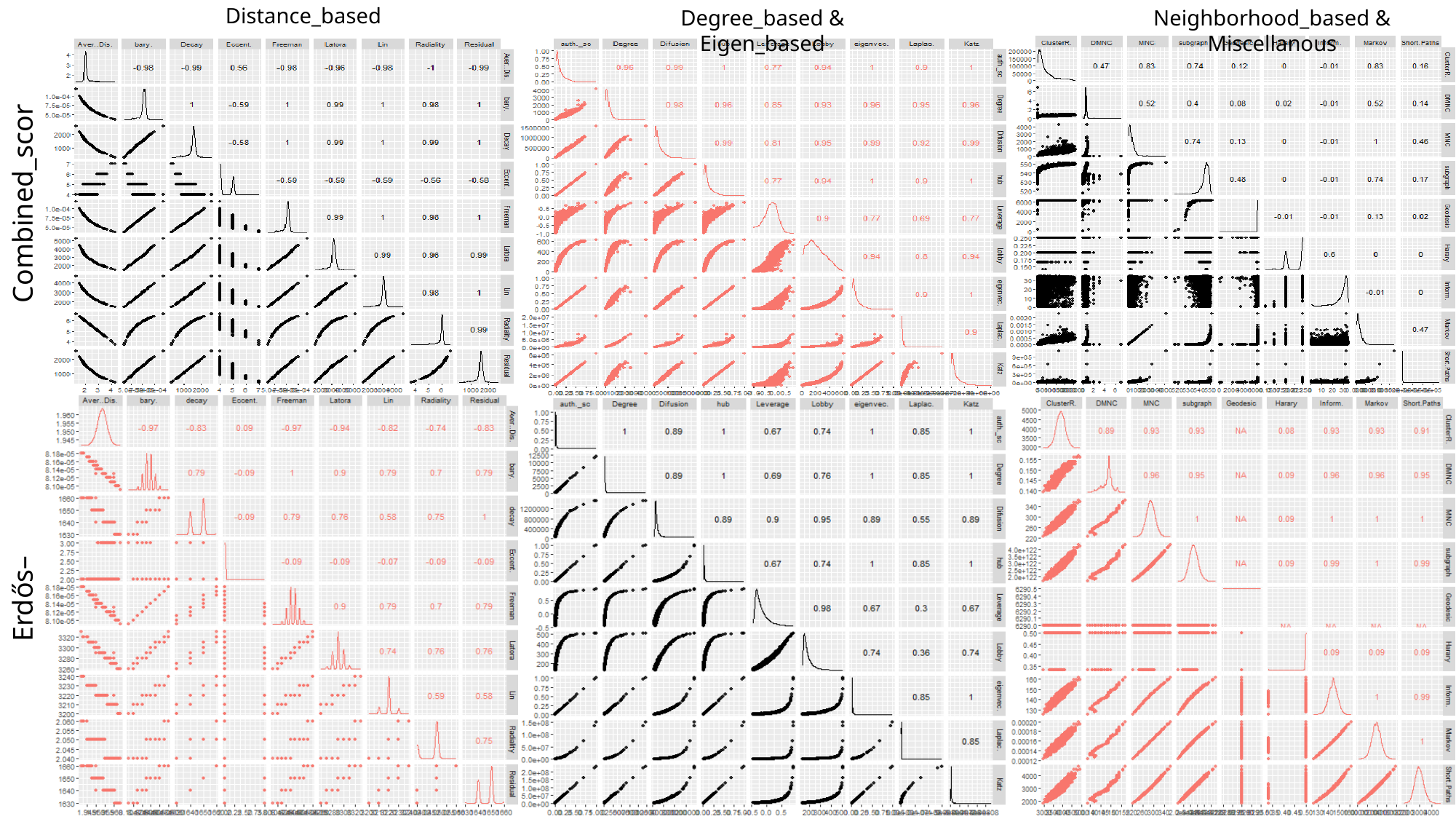

Distance_based
Degree_based & Eigen_based
Neighborhood_based & Miscellanous
Combined_score
Erdős–Rényi
