# Supplementary material for: A systematic survey of centrality measures for protein-protein interaction networks
Source: BMC Syst Biol. 2018 Jul 31;12:80. doi: 10.1186/s12918-018-0598-2 (PMC6069823; doi:10.1186/s12918-018-0598-2)

“Coexpression”

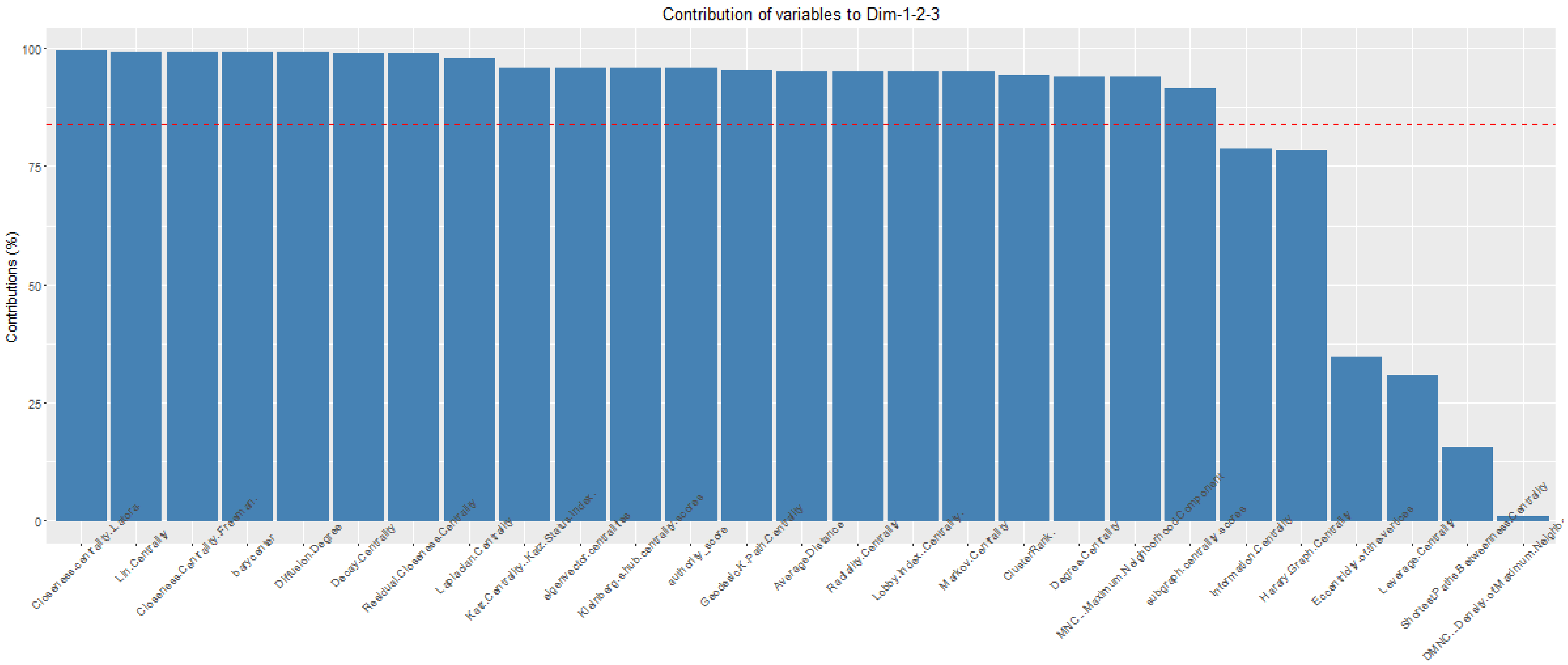

“Coexpression_transferred”

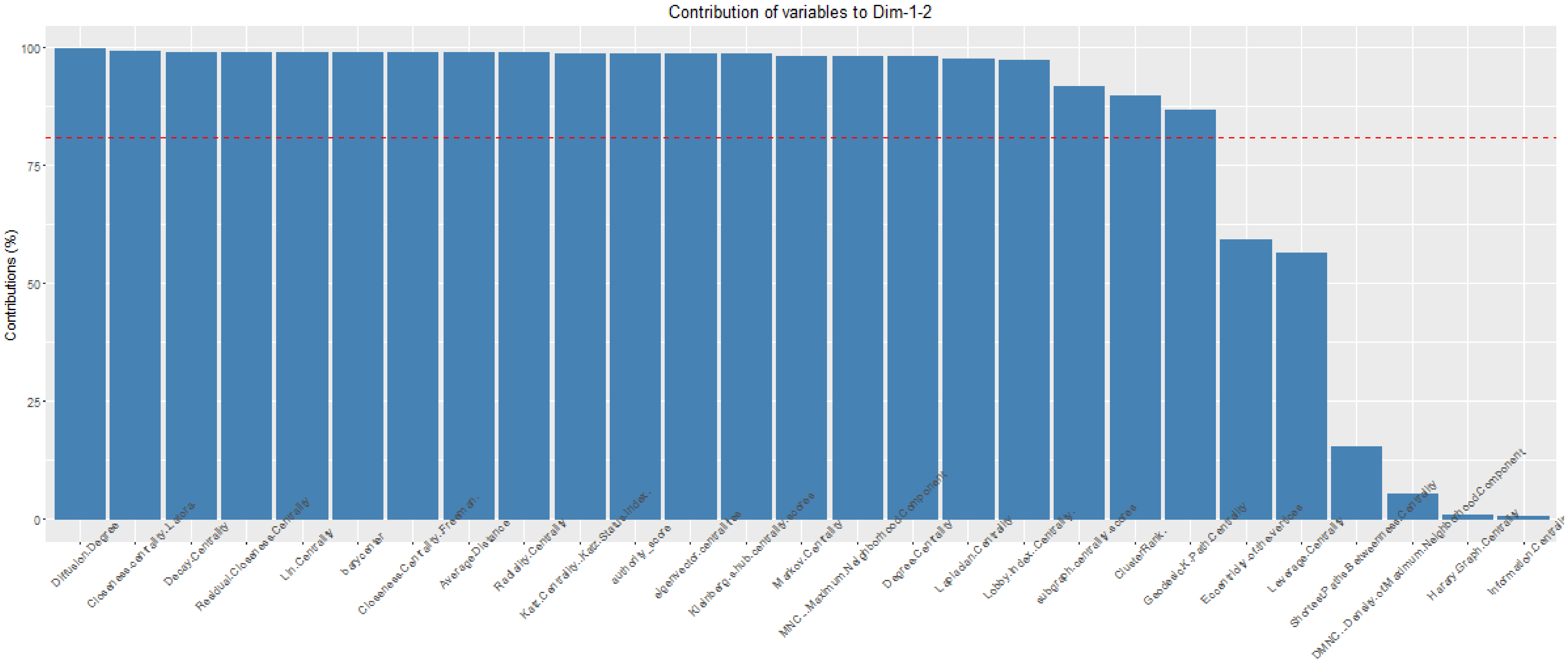

“Cooccurence”

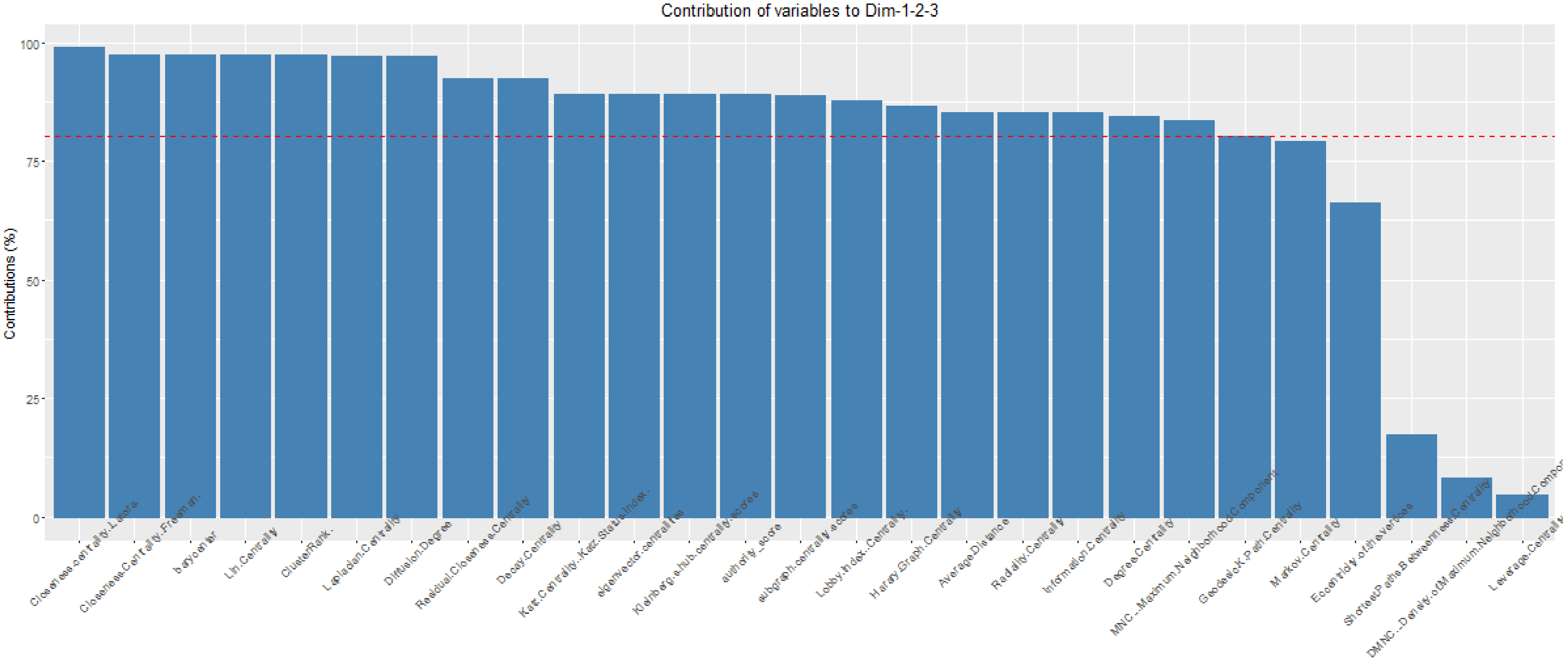

“Database”

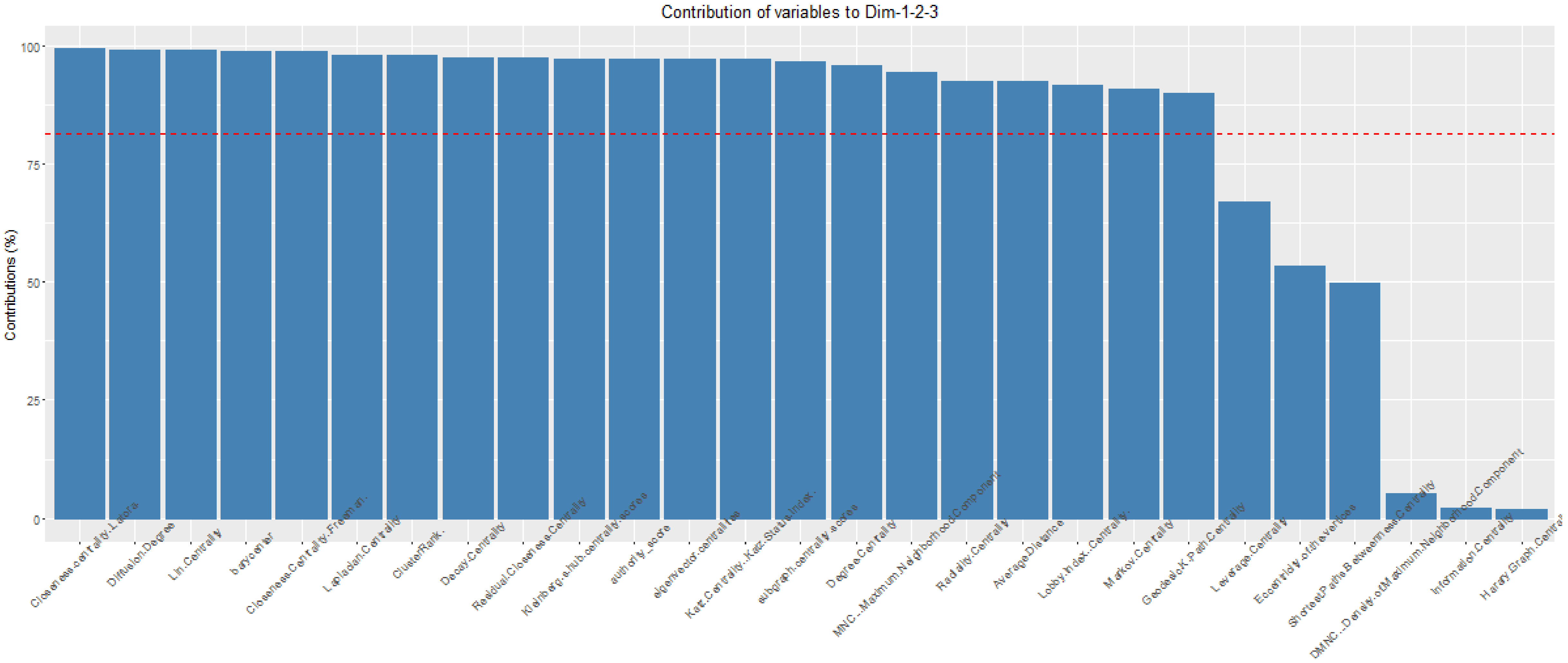
Contribution of variables to Dim-1-2-3
Contributions (%)
100
75
50
25
0
Closeness.centrality.Latora
Diffusion.Degree
Lin.Centrality
barycenter
Closeness.Centrality.Freeman.
Laplacian.Centrality
ClusterRank.
Decay.Centrality
Residual.Closeness.Centrality
Kleinberg.s.hub.centrality.scores
authority_score
eigenvector.centralities
Katz.Centrality..Katz.Status.Index.
subgraph.centrality.scores
Degree.Centrality
MNC...Maximum.Neighborhood.Component
Radiality.Centrality
Average.Distance
Lobby.Index..Centrality.
Markov.Centrality
Geodesic.K.Path.Centrality
Leverage.Centrality
Eccentricity.of.the.vertices
Shortest.Paths.Betweenness.Centrality
DMNC...Density.of.Maximum.Neighborhood.Component
Information.Centrality

“Database_transferred”

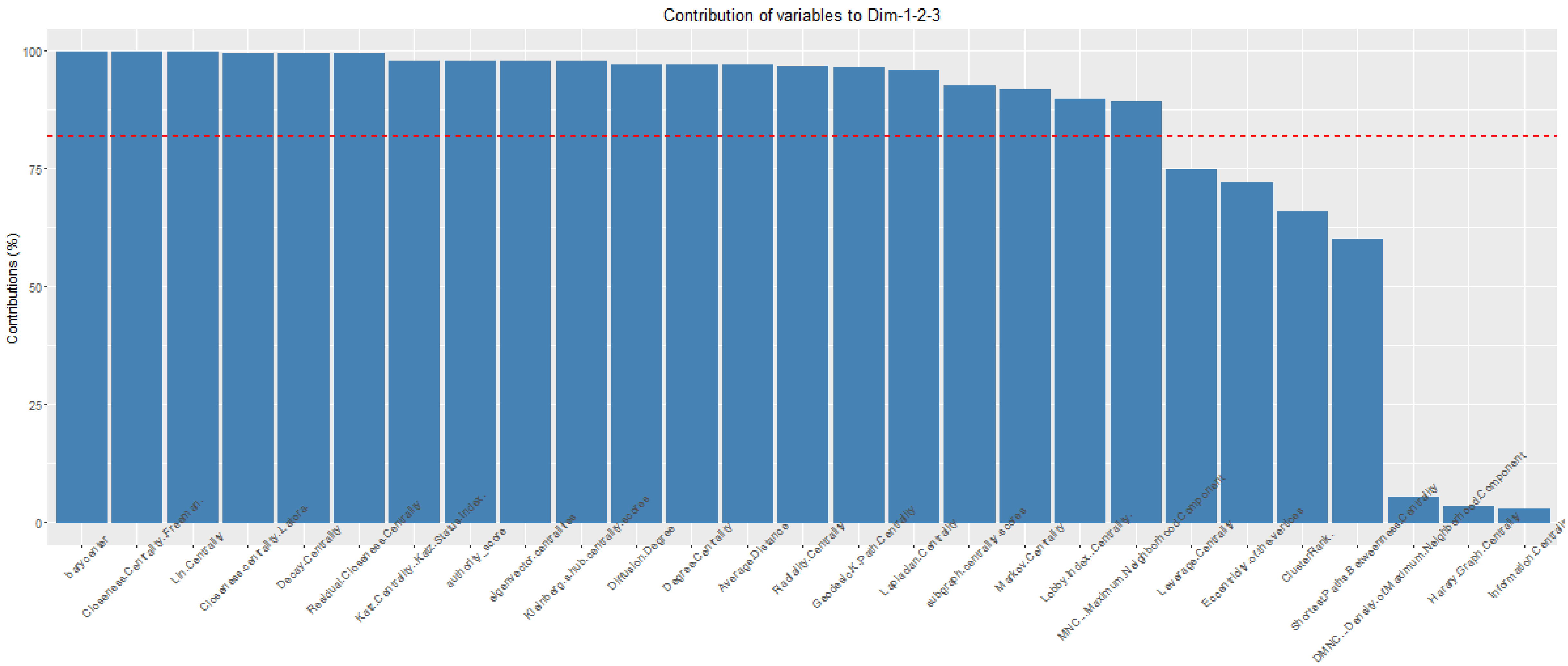

“Experiments”

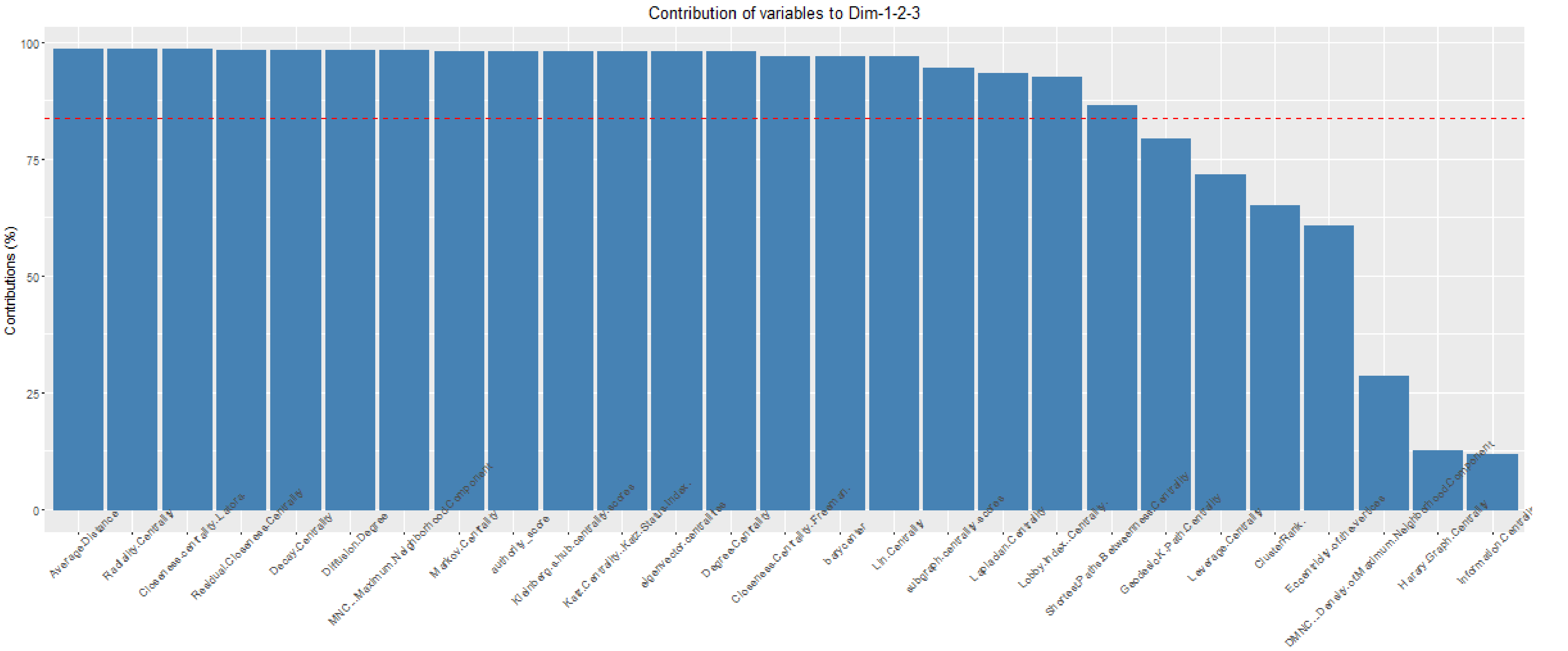

“Experiments_transferred”

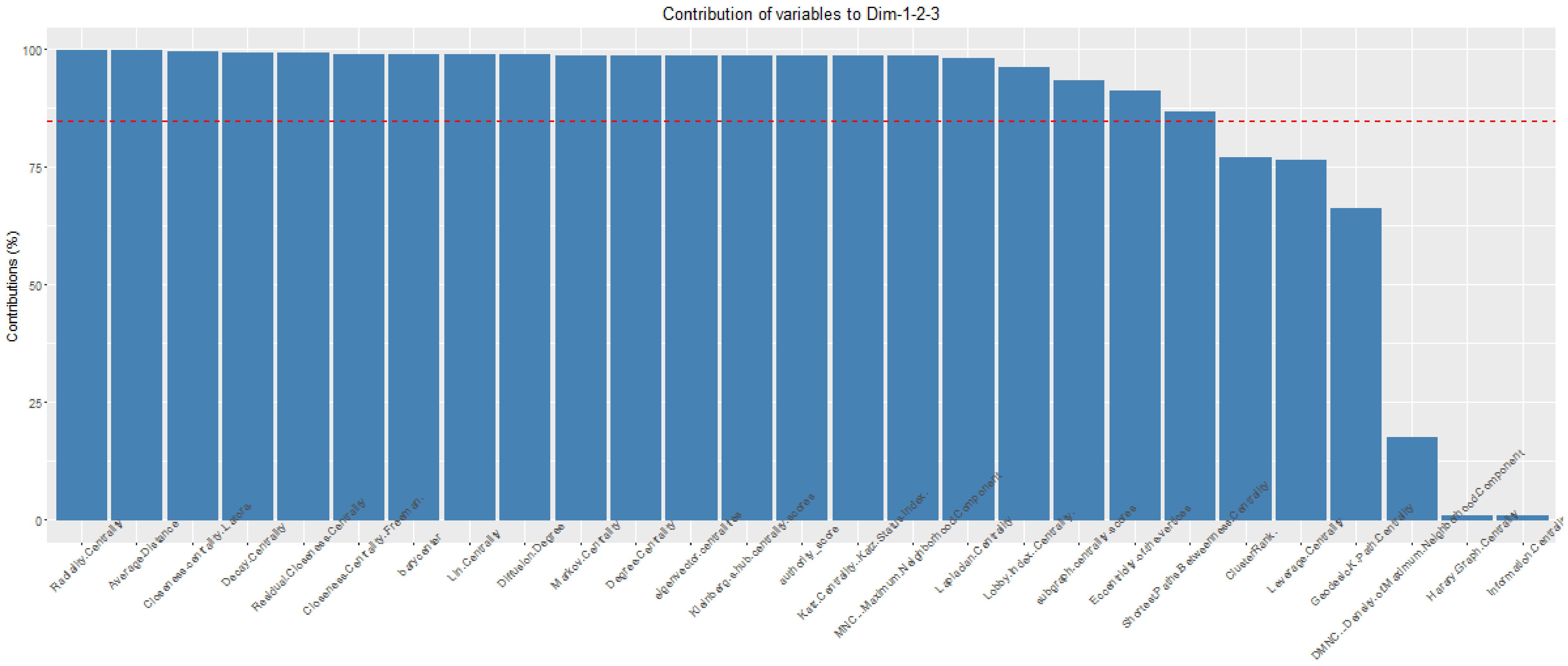

“Textmining”

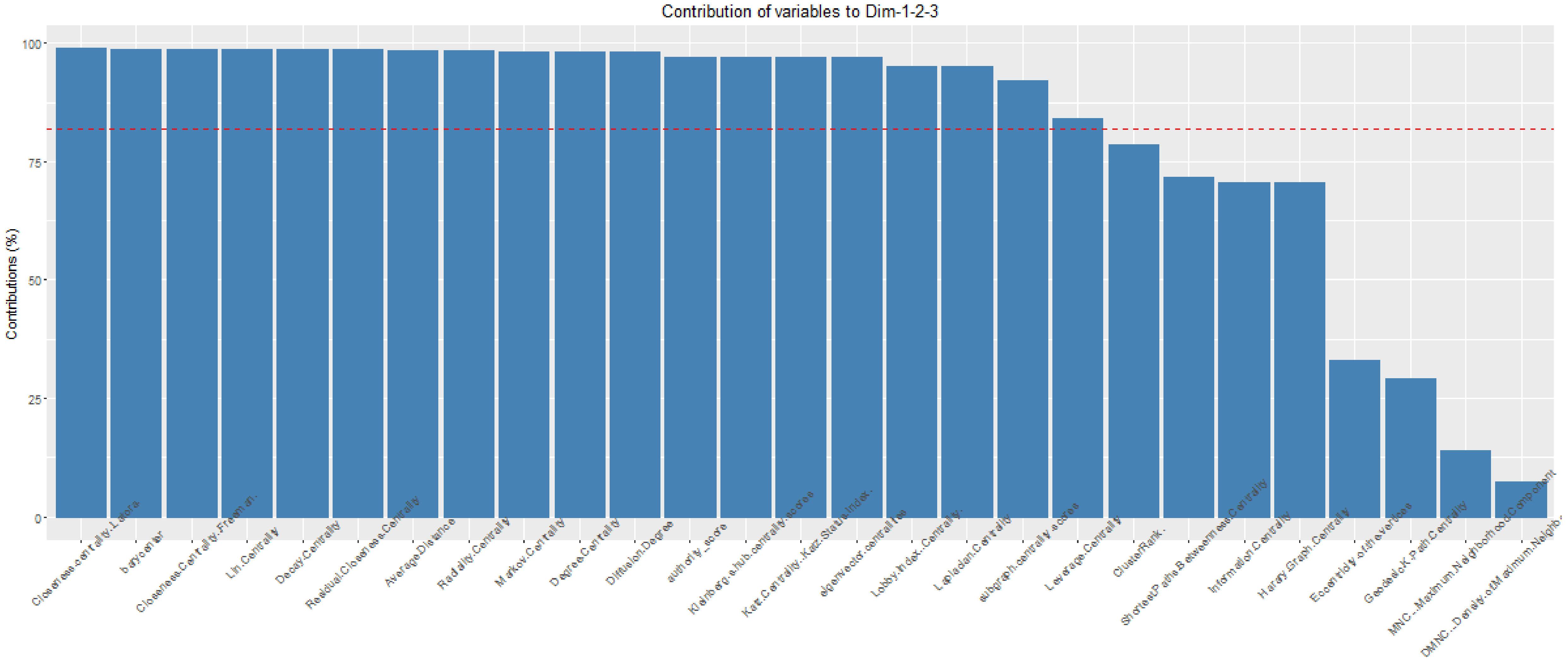

Contribution of variables to Dim-1-2-3
Contributions (%)
100
75
50
25
0
Closeness.centrality.Latora
barycenter
Closeness.Centrality.Freeman.
Lin.Centrality
Decay.Centrality
Residual.Closeness.Centrality
Average.Distance
Radiality.Centrality
Markov.Centrality
Degree.Centrality
Diffusion.Degree
authority_score
Kleinberg.s.hub.centrality.scores
Katz.Centrality..Katz.Status.Index.
eigenvector.centralities
Lobby.Index..Centrality.
Laplacian.Centrality
subgraph.centrality.scores
Leverage.Centrality
ClusterRank.
Shortest.Paths.Betweenness.Centrality
Information.Centrality
Harary.Graph.Centrality
Eccentricity.of.the.vertices
Geodesic.K.Path.Centrality
MNC...Maximum.Neighborhood.Component.
DMNC...Density.of.Maximum.Neighb...

“Textmining_transferred”

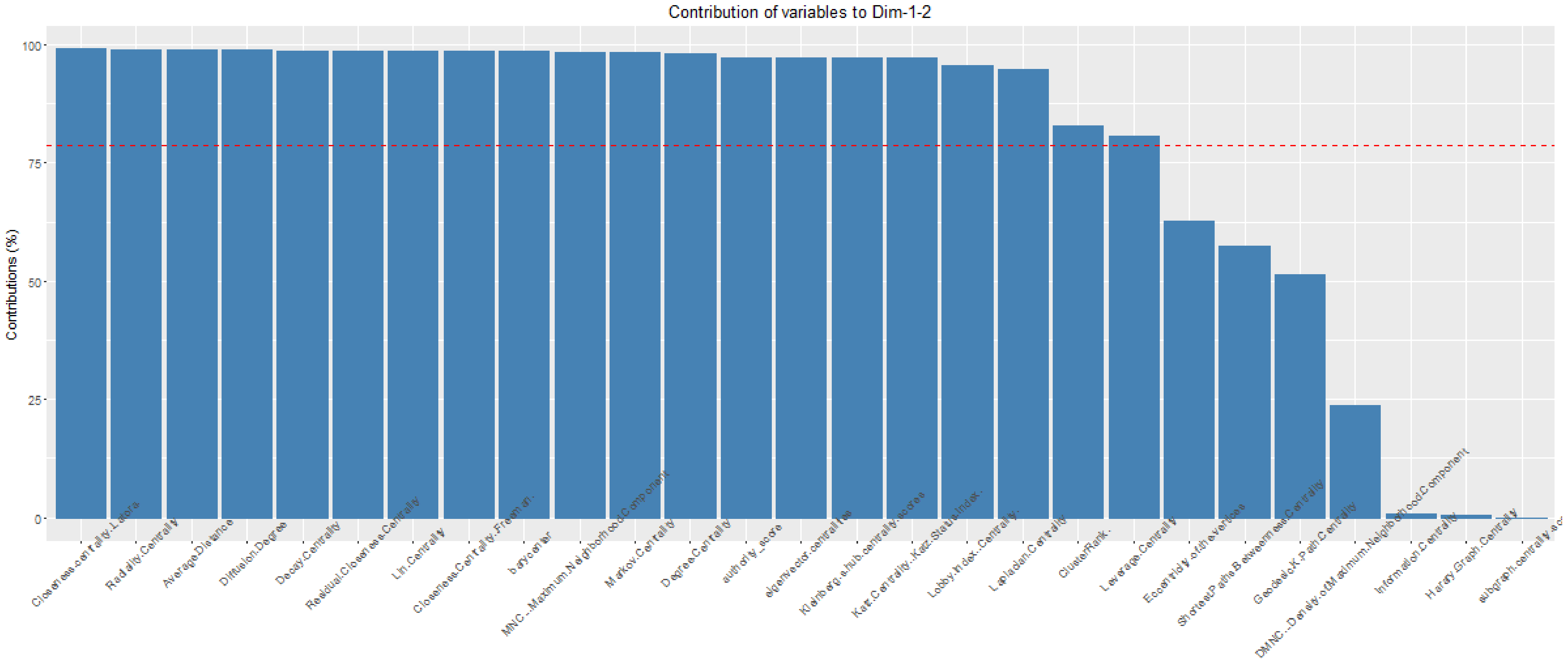

“Neighborhood_transferred”

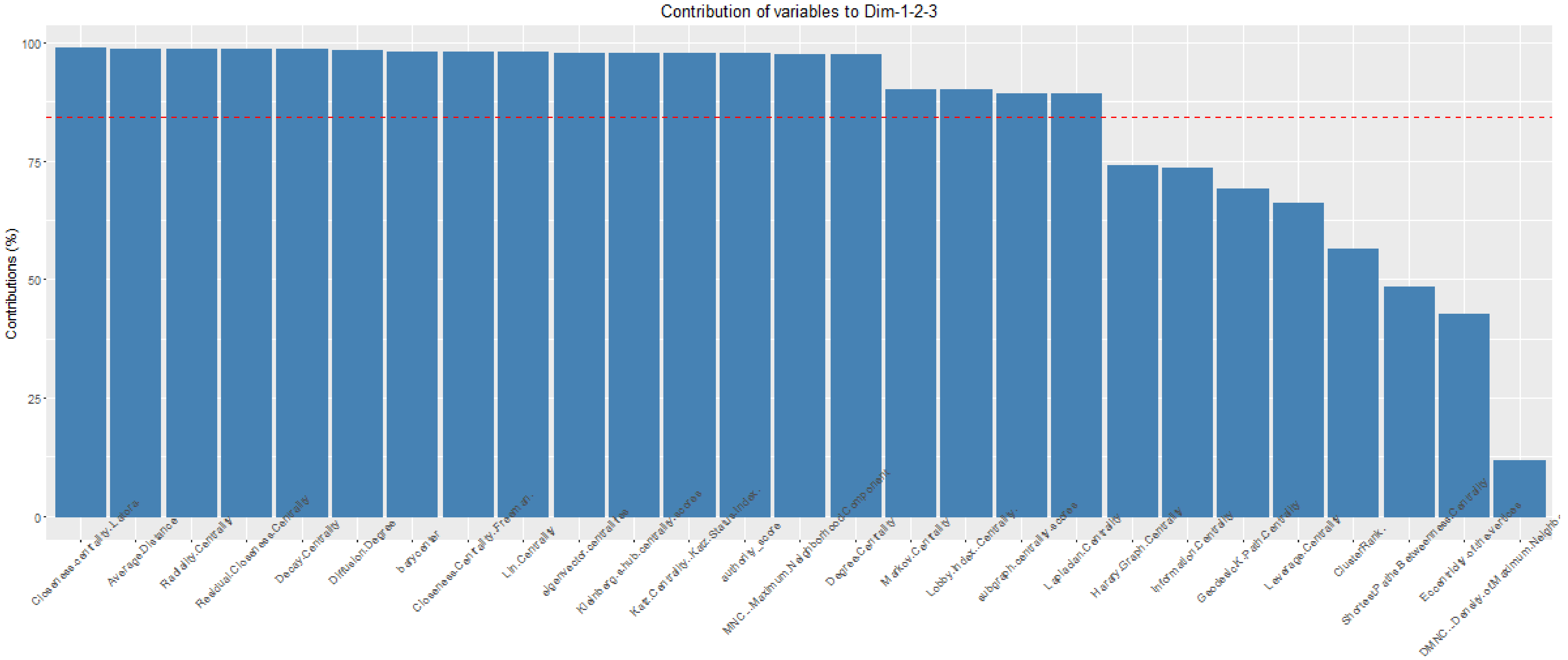

“Fusion”

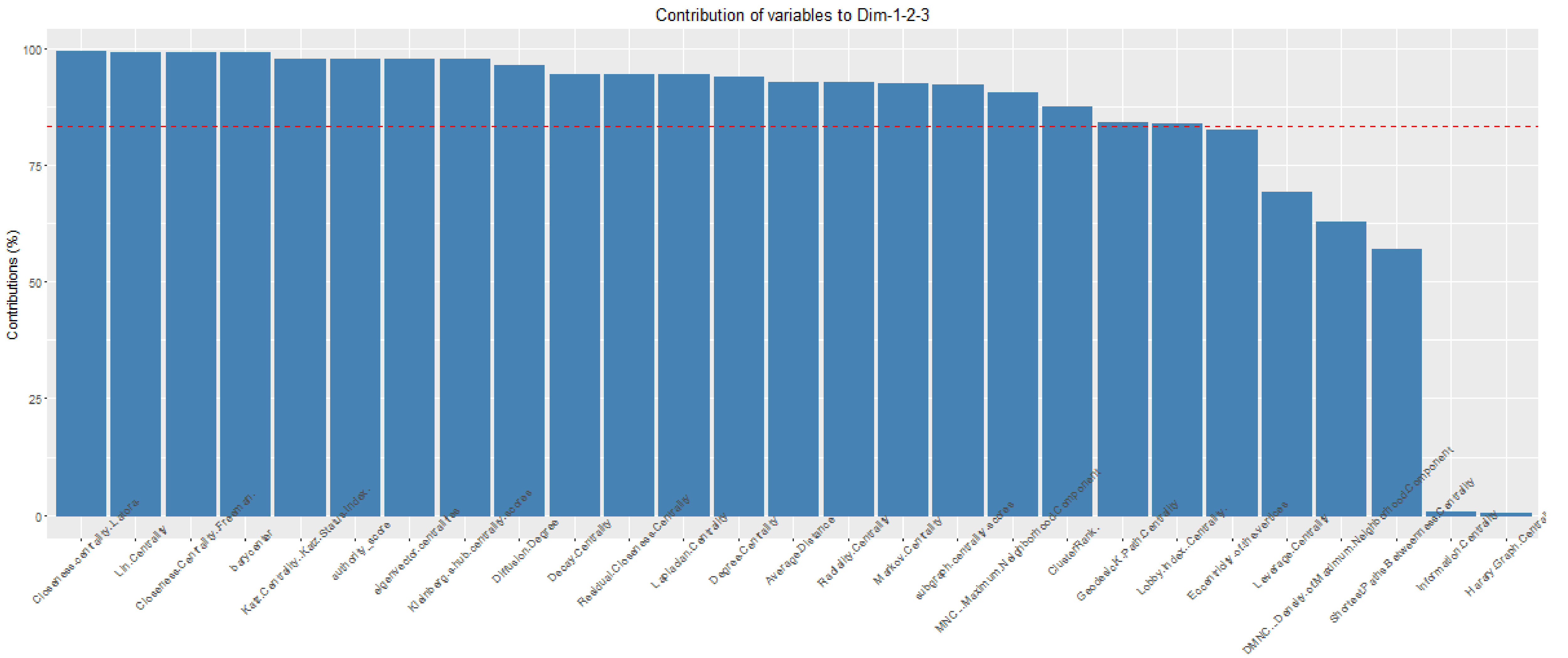

Contribution of variables to Dim-1-2-3
Contributions (%)
100
75
50
25
0
Closeness.centrality.Latora
Lin.Centrality
Closeness.Centrality.Freeman.
barycenter
Katz.Centrality..Katz.Status.Index.
authority_score
eigenvector.centralities
Kleinberg.s.hub.centrality.scores
Diffusion.Degree
Decay.Centrality
Residual.Closeness.Centrality
Laplacian.Centrality
Degree.Centrality
Average.Distance
Radiality.Centrality
Markov.Centrality
subgraph.centrality.scores
MNC...Maximum.Neighborhood.Component
ClusterRank.
Geodesic.K.Path.Centrality
Lobby.Index..Centrality.
Eccentricity.of.the.vertices
Leverage.Centrality
DMNC...Density.of.Maximum.Neighborhood.Component
Shortest.Paths.Betweenness.Centrality
Information.Centrality
Harary.Graph.Centrality

“Combined_score”

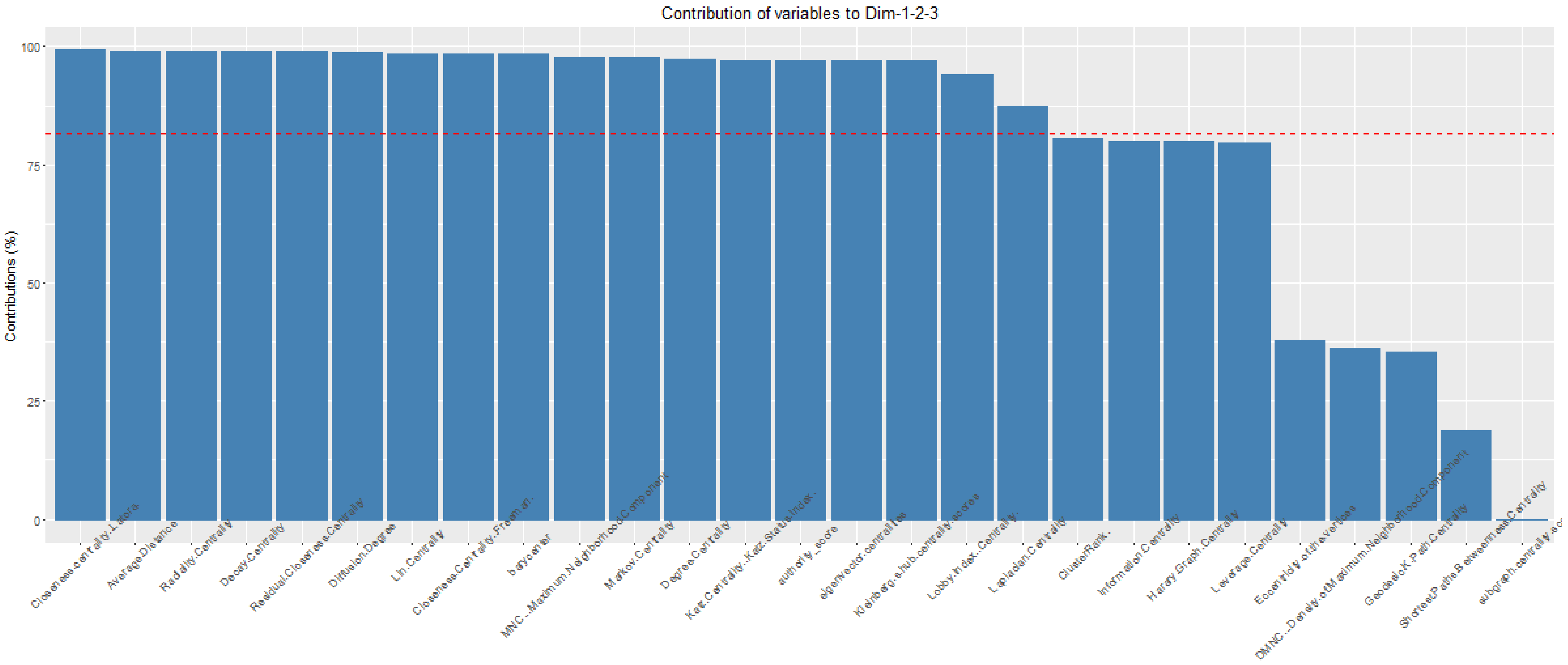

“Homology”

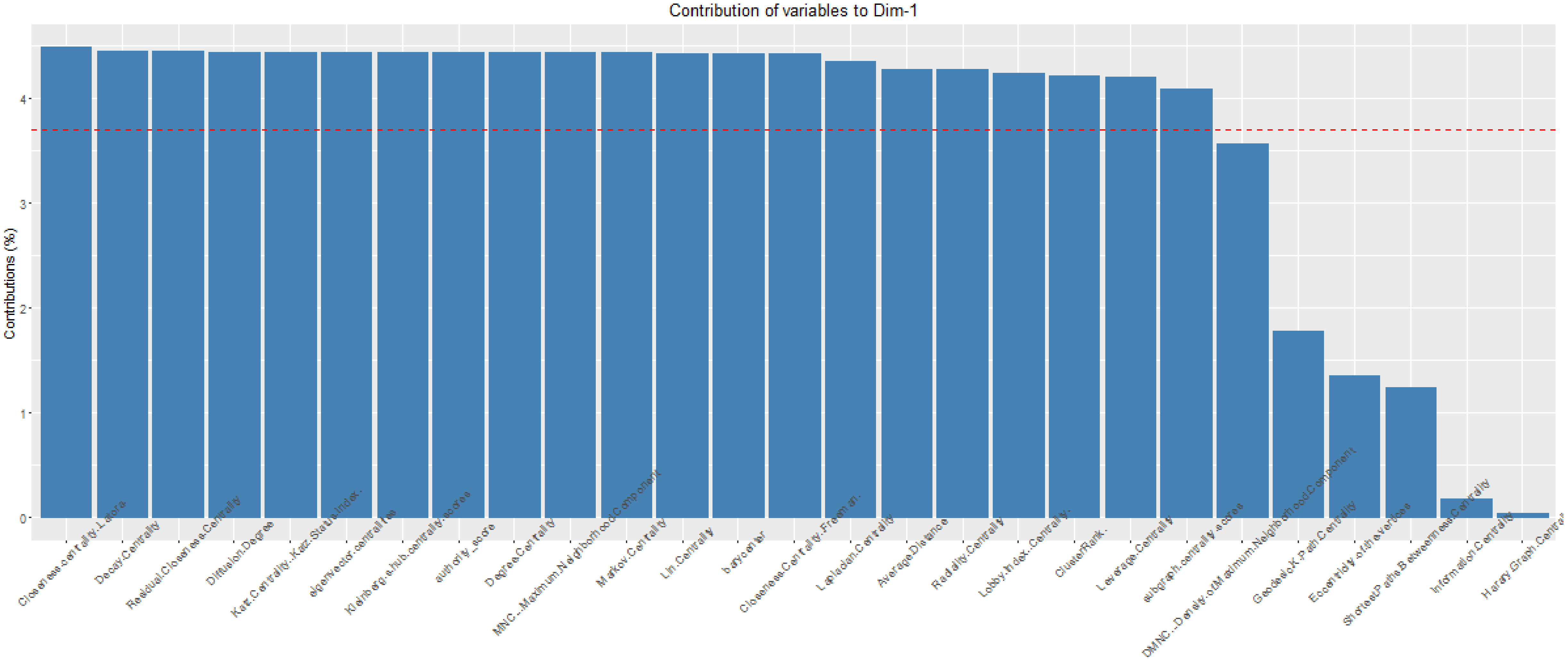
Contribution of variables to Dim-1
Contributions (%)
0
1
2
3
4
Closeness.centrality.Latora
Decay.Centrality
Residual.Closeness.Centrality
Diffusion.Degree
Katz.Centrality..Katz.Status.Index.
eigenvector.centralities
Kleinberg.s.hub.centrality.scores
authority_score
Degree.Centrality
MNC...Maximum.Neighborhood.Component
Markov.Centrality
Lin.Centrality
barycenter
Closeness.Centrality..Freeman.
Laplacian.Centrality
Average.Distance
Radiality.Centrality
Lobby.Index..Centrality.
ClusterRank.
Leverage.Centrality
subgraph.centrality.scores
DMNC...Density.of.Maximum.Neighborhood.Component
Geodesic.K.Path.Centrality
Eccentricity.of.the.vertices
Shortest.Paths.Betweenness.Centrality
Information.Centrality

“Erdős–Rényi”

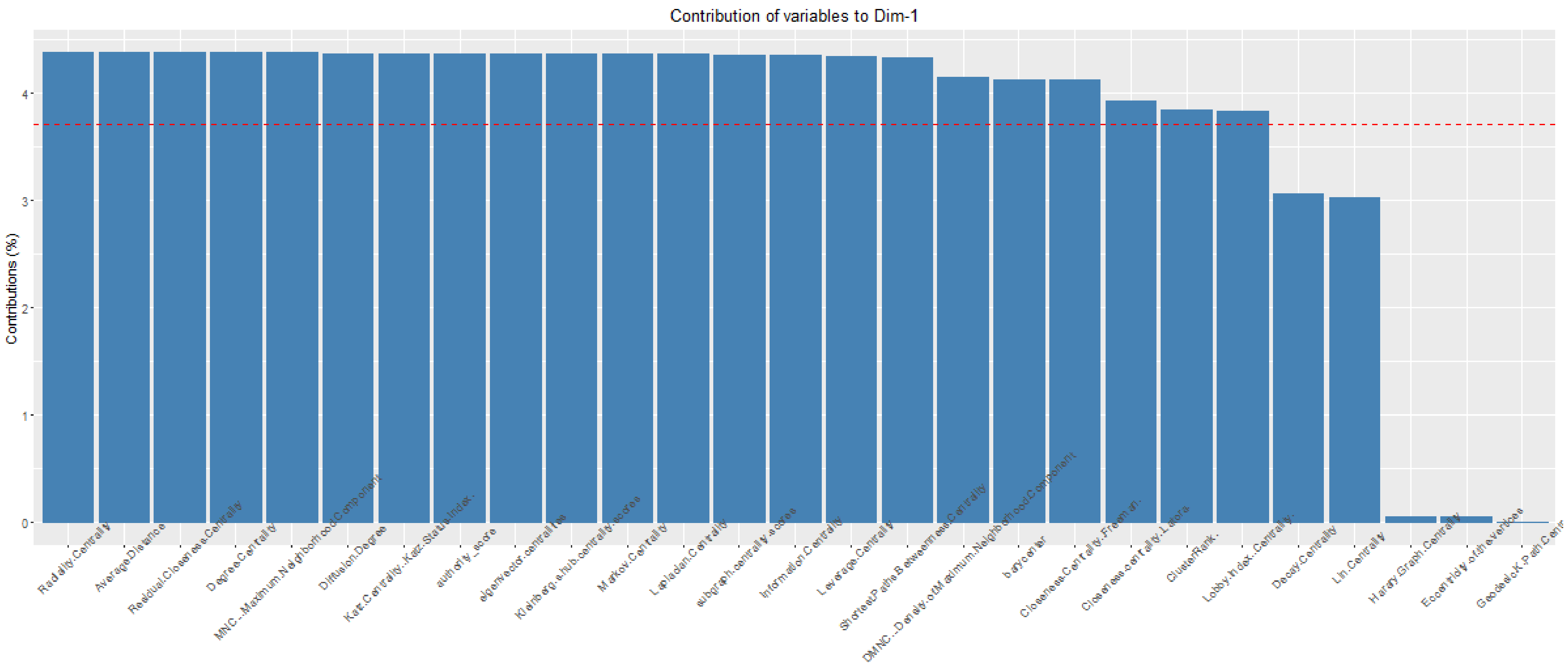

Supplement: Supplementary file 4 — Contribution values of centralities in each network. These values were computed based on the principal components. The red line shows the threshold used for identifying effective centralities. (PDF 441 kb) [file 12918_2018_598_MOESM4_ESM.pdf]
