# Supplementary material for: A systematic survey of centrality measures for protein-protein interaction networks
Source: BMC Syst Biol. 2018 Jul 31;12:80. doi: 10.1186/s12918-018-0598-2 (PMC6069823; doi:10.1186/s12918-018-0598-2)

"Coexpression"

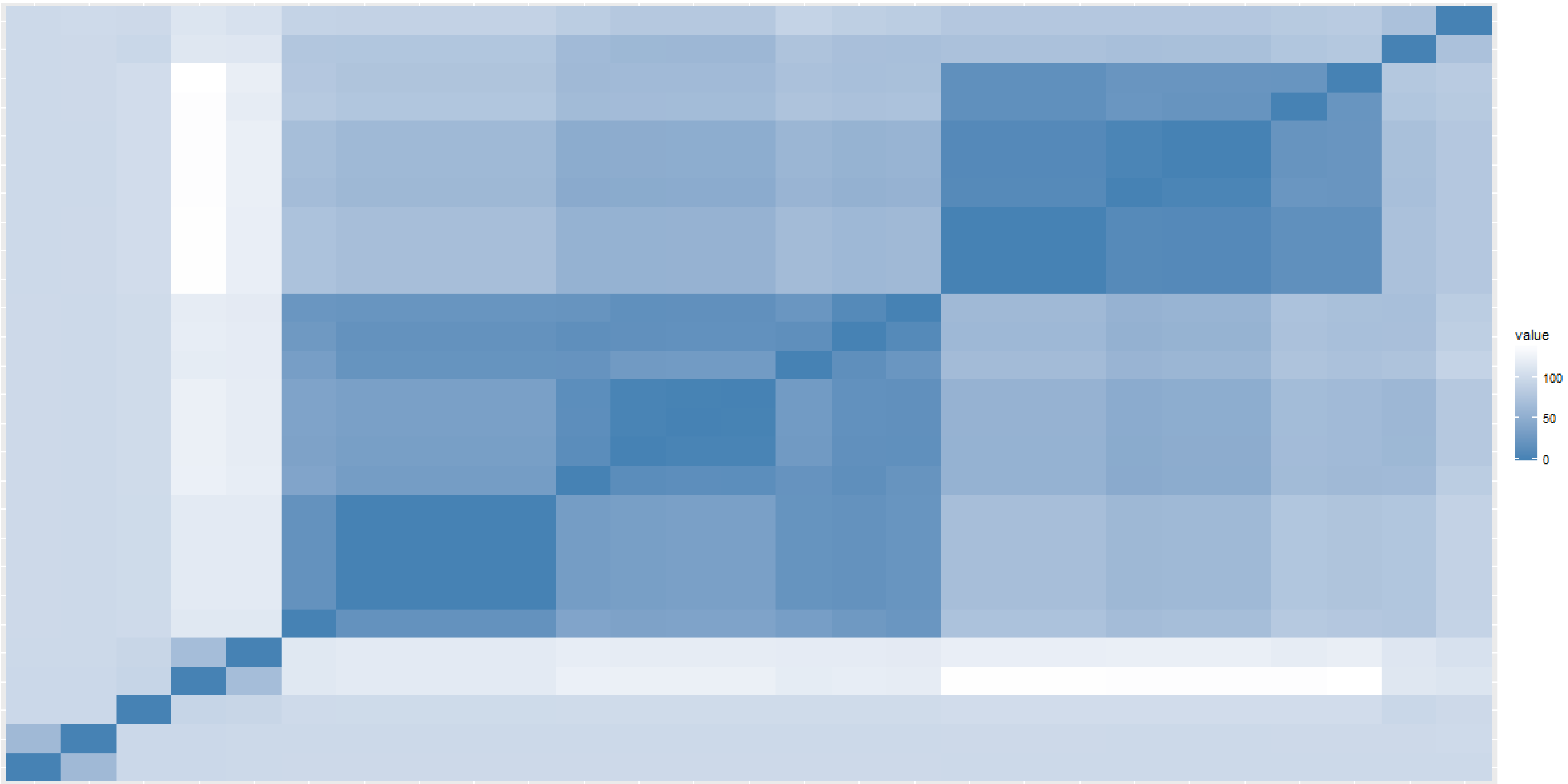

"Coexpression_transferred"

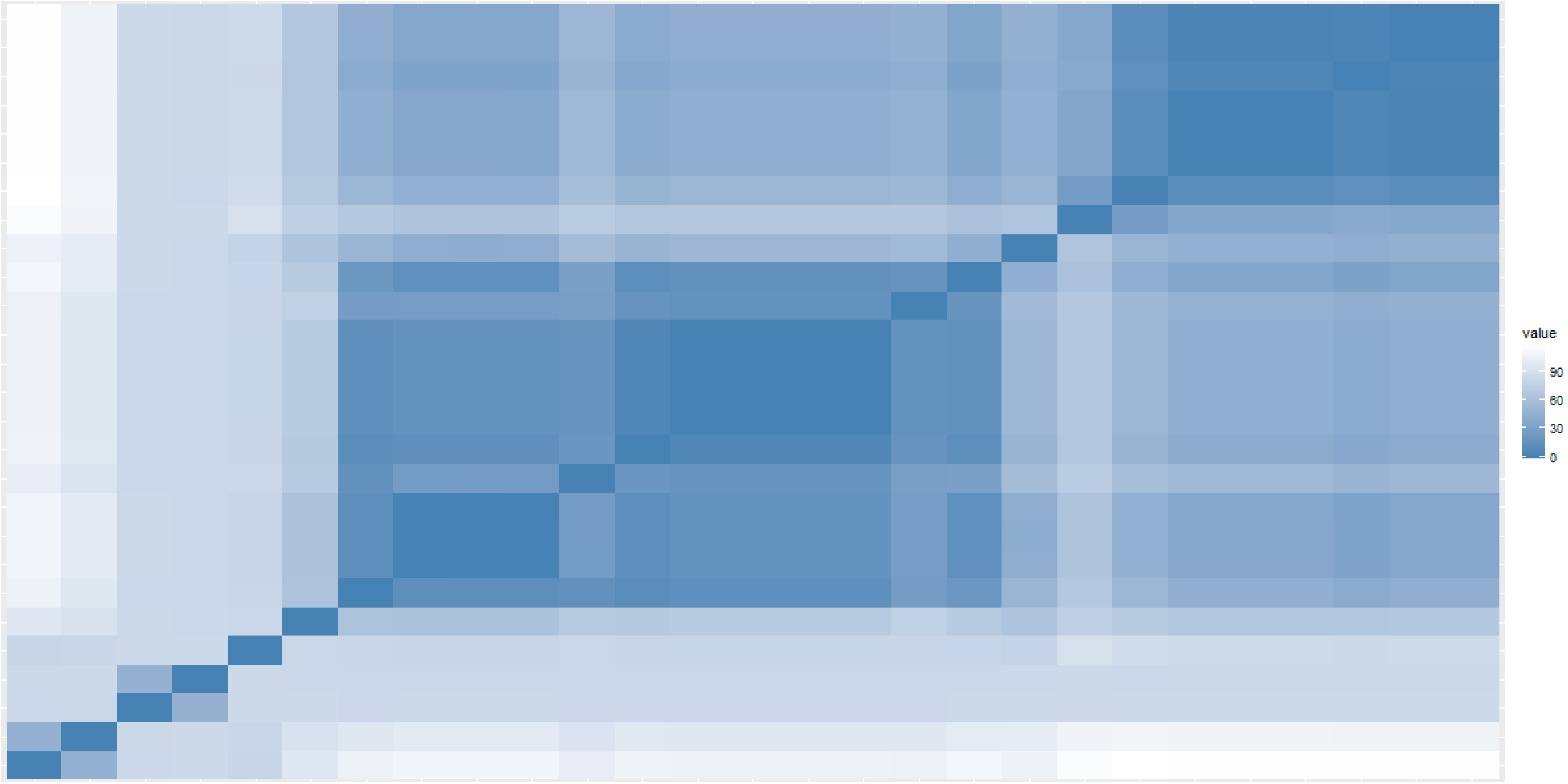

“Cooccurence”

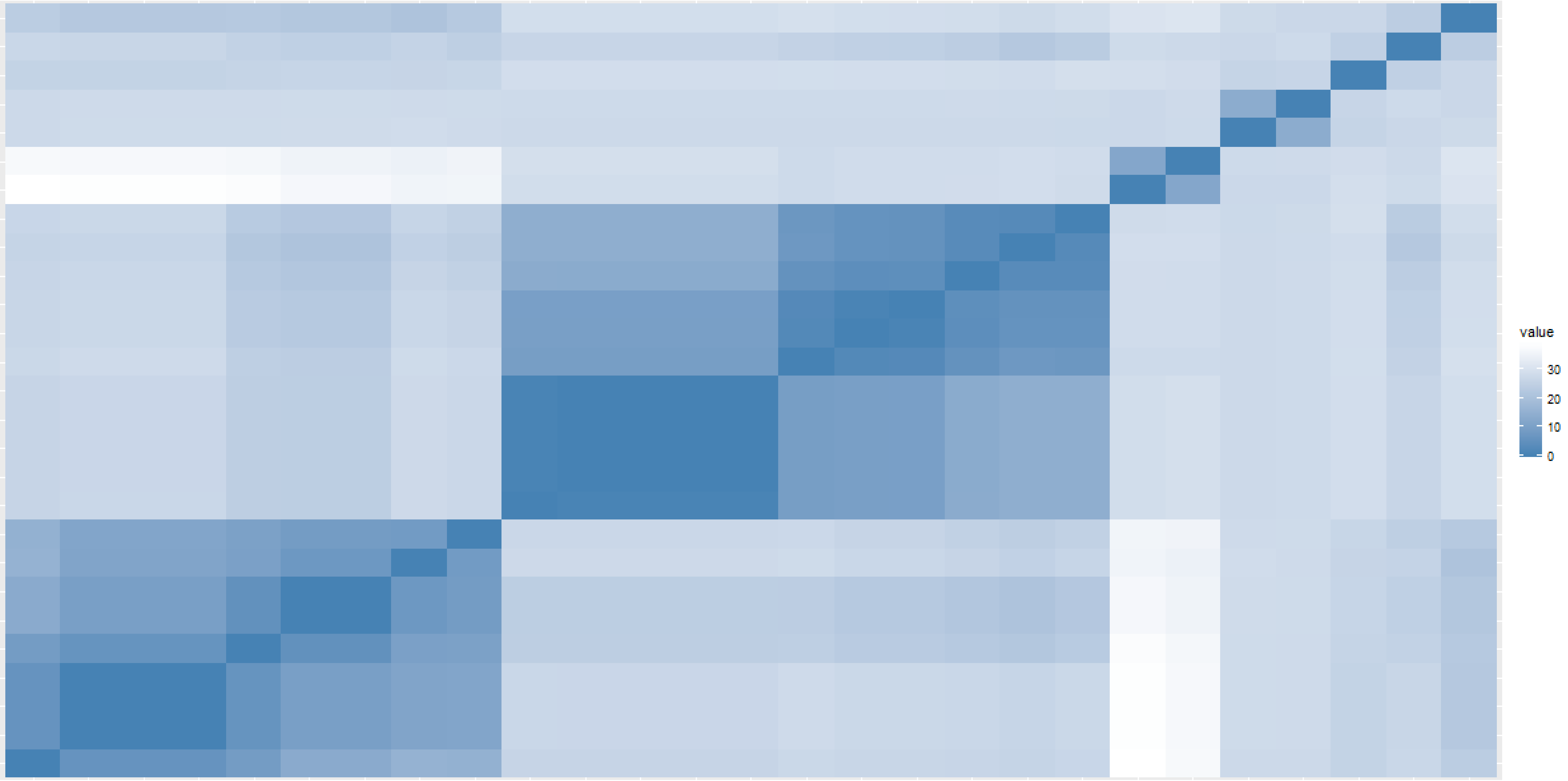

“Database”

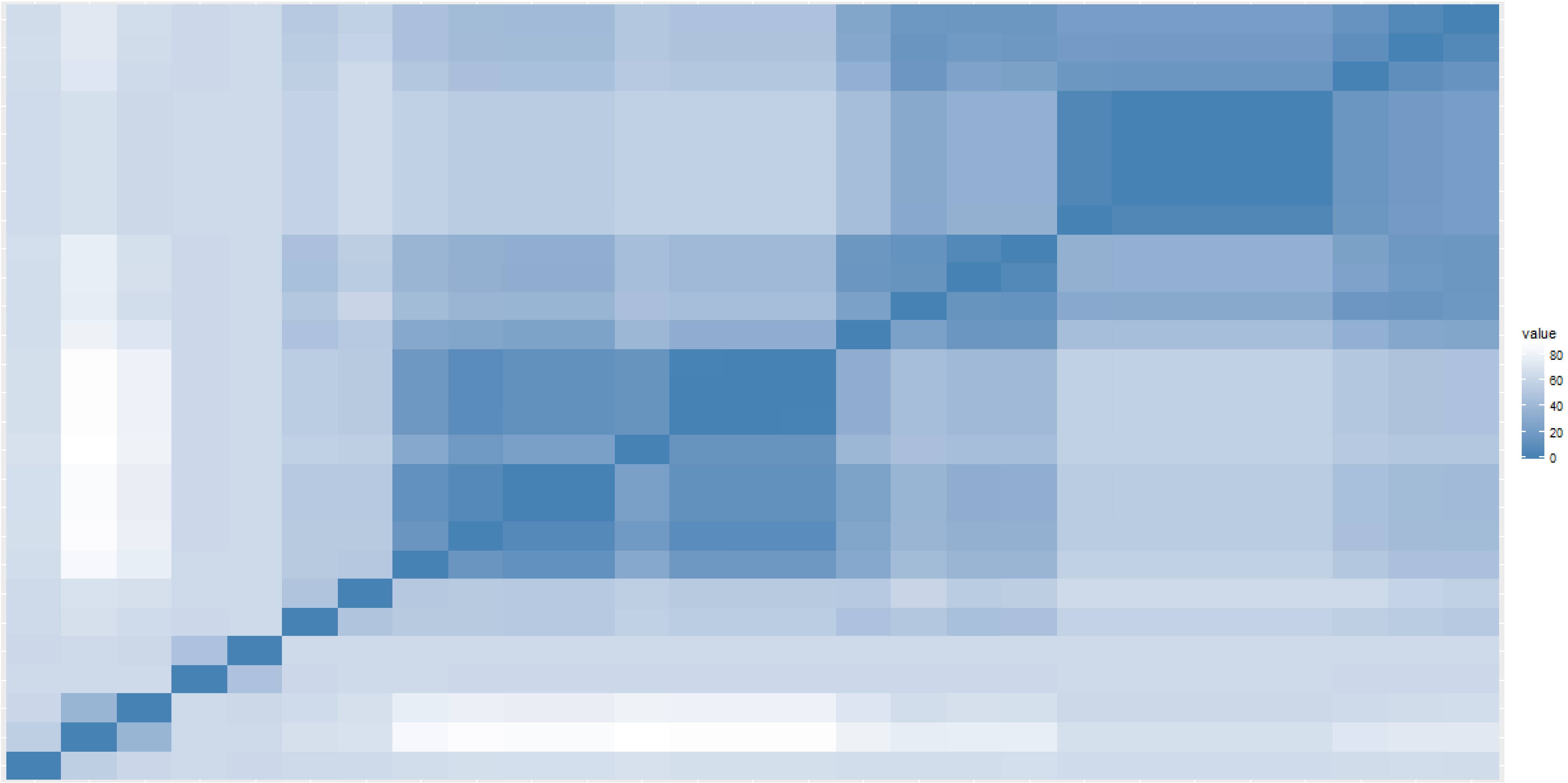

“Database_transferred”

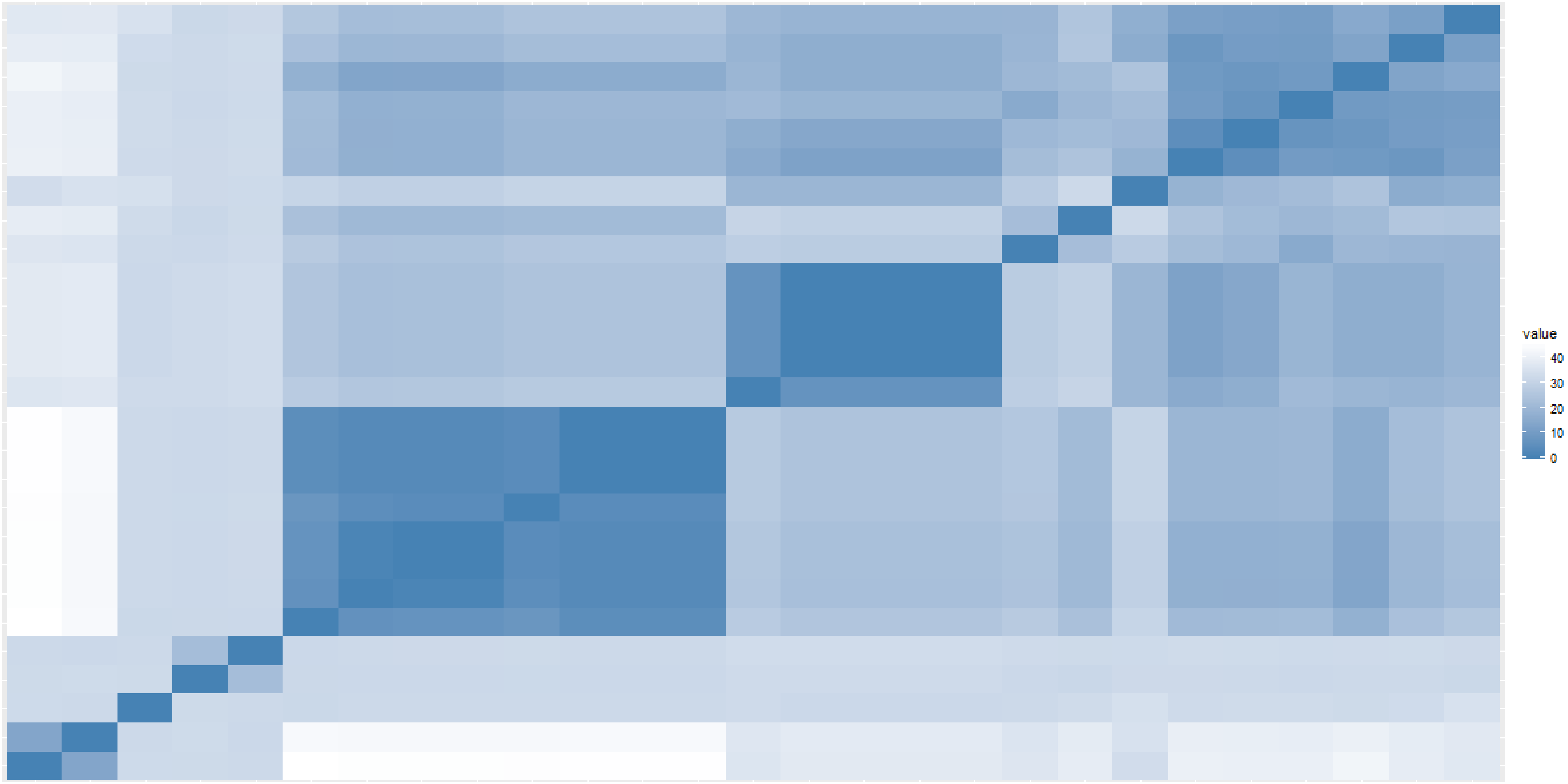

“Experiments”

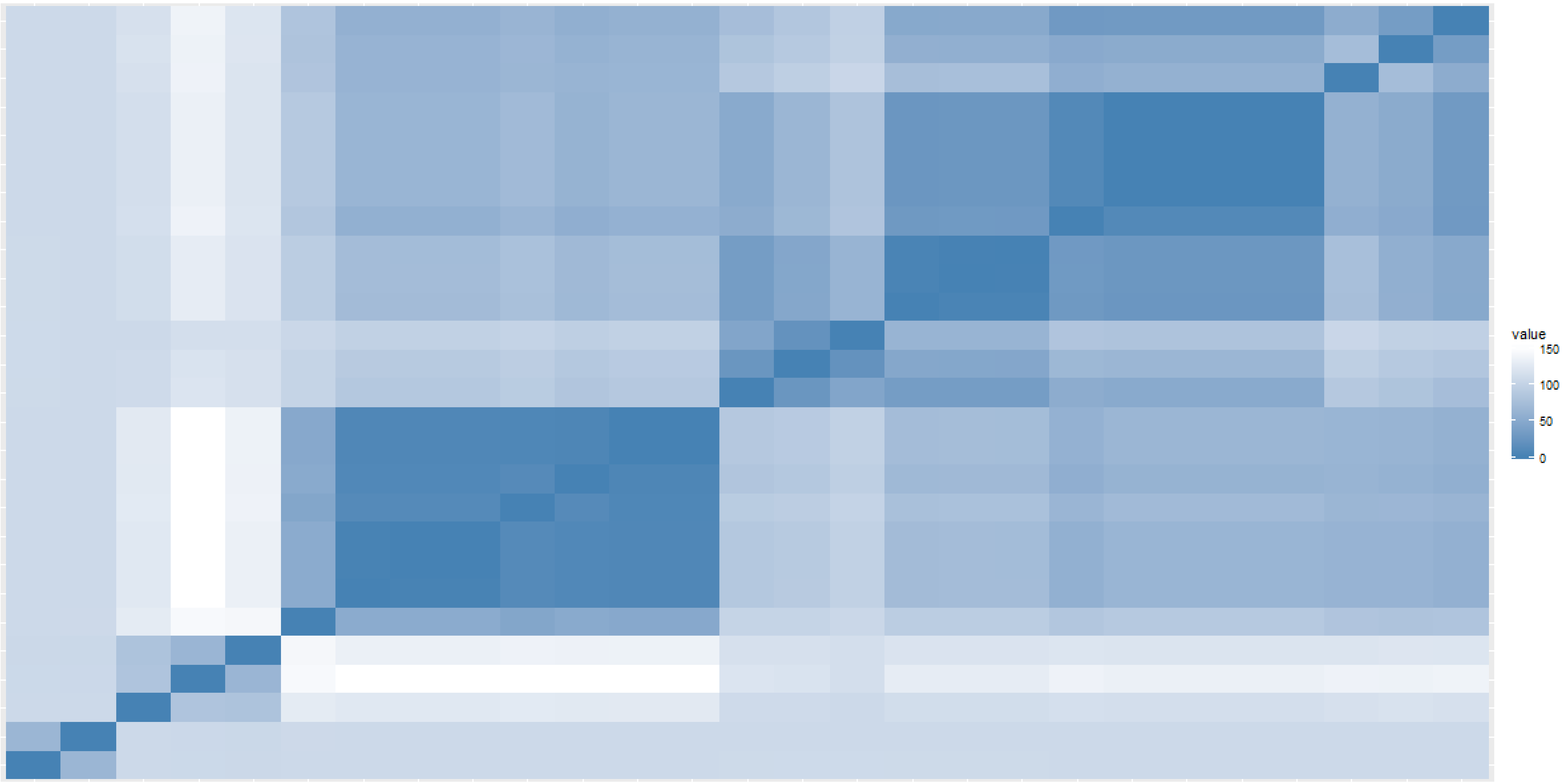

“Experiments_transferred”

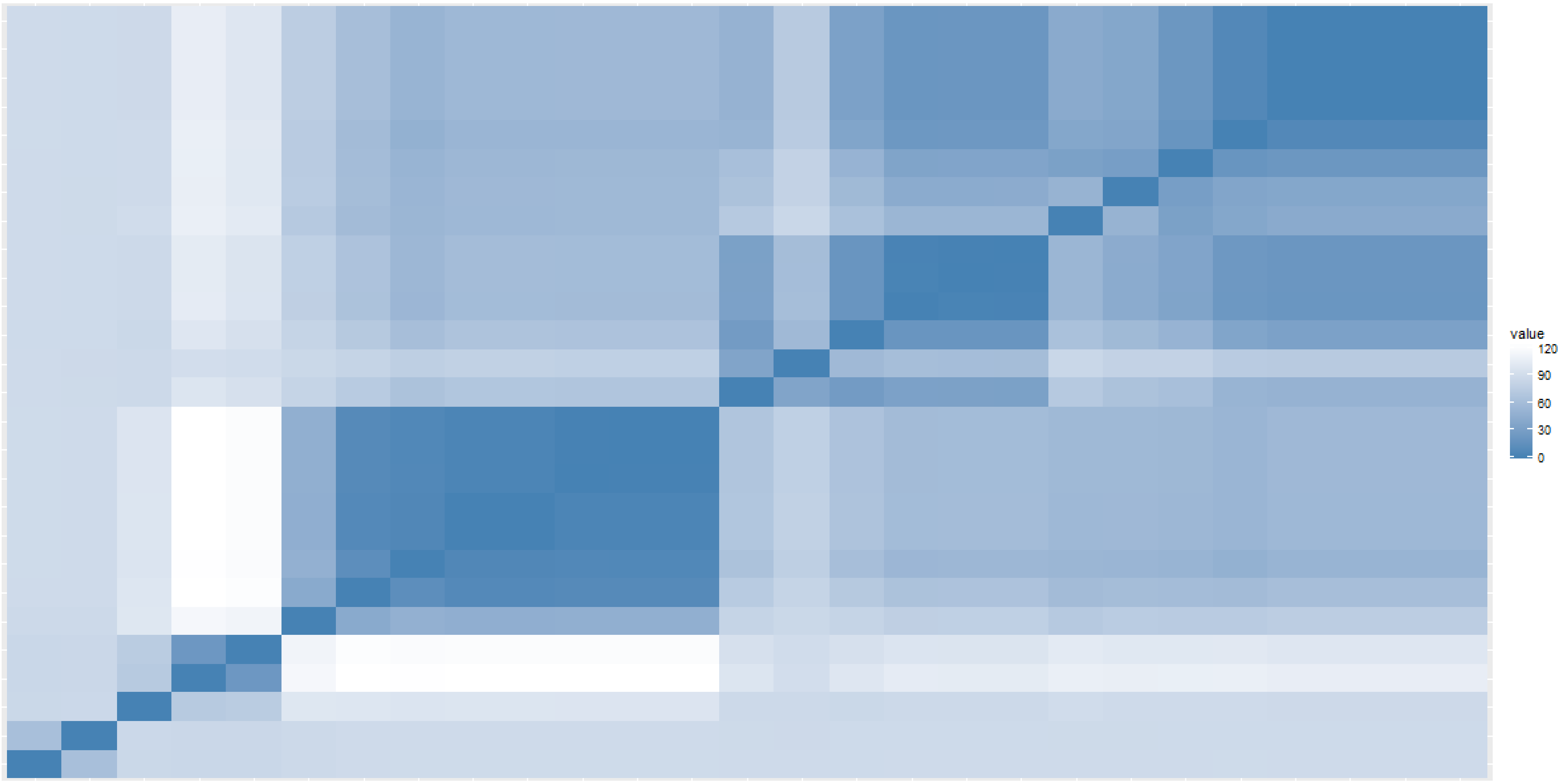

“Textmining”

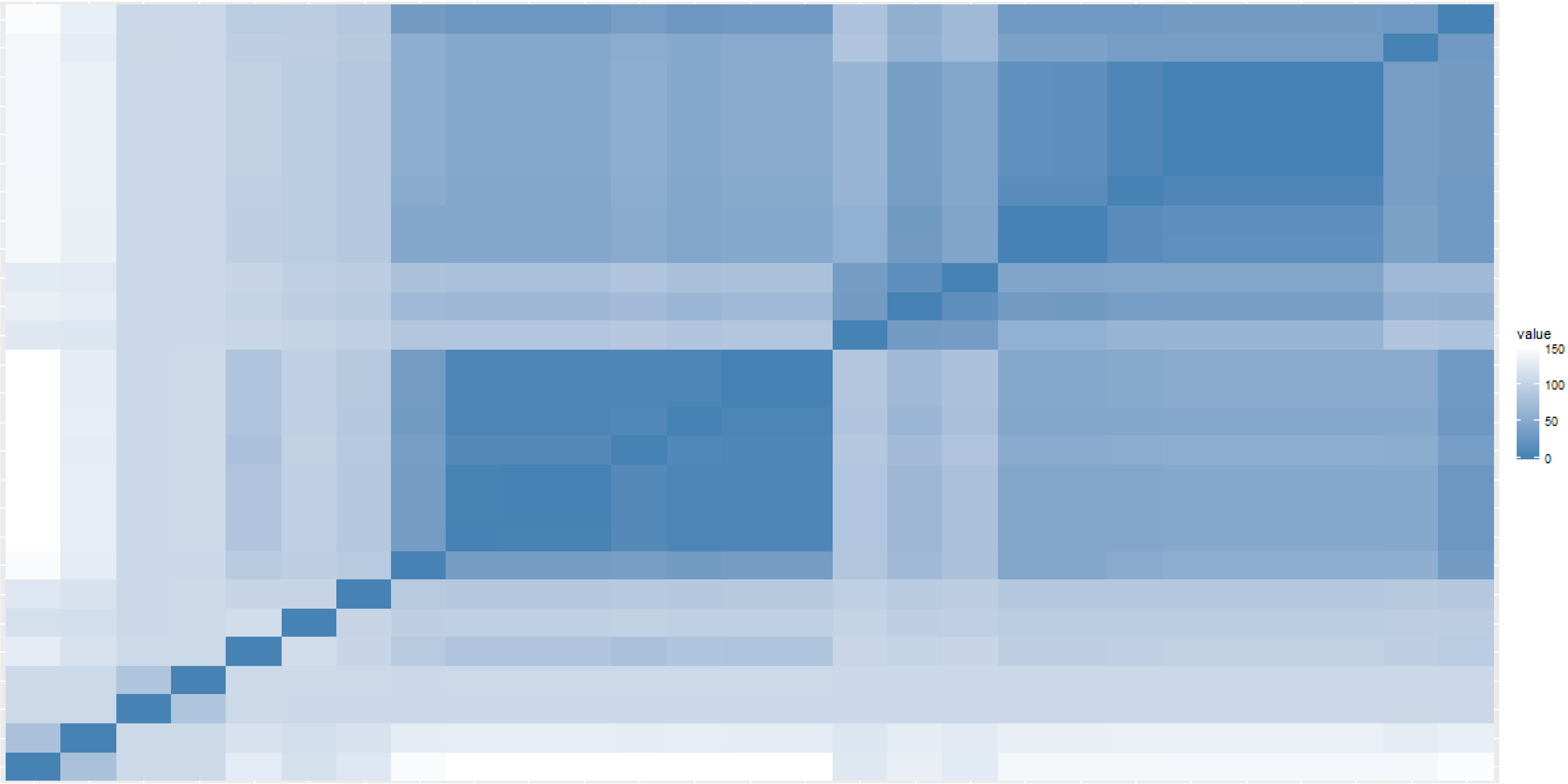

“Textmining_transferred”

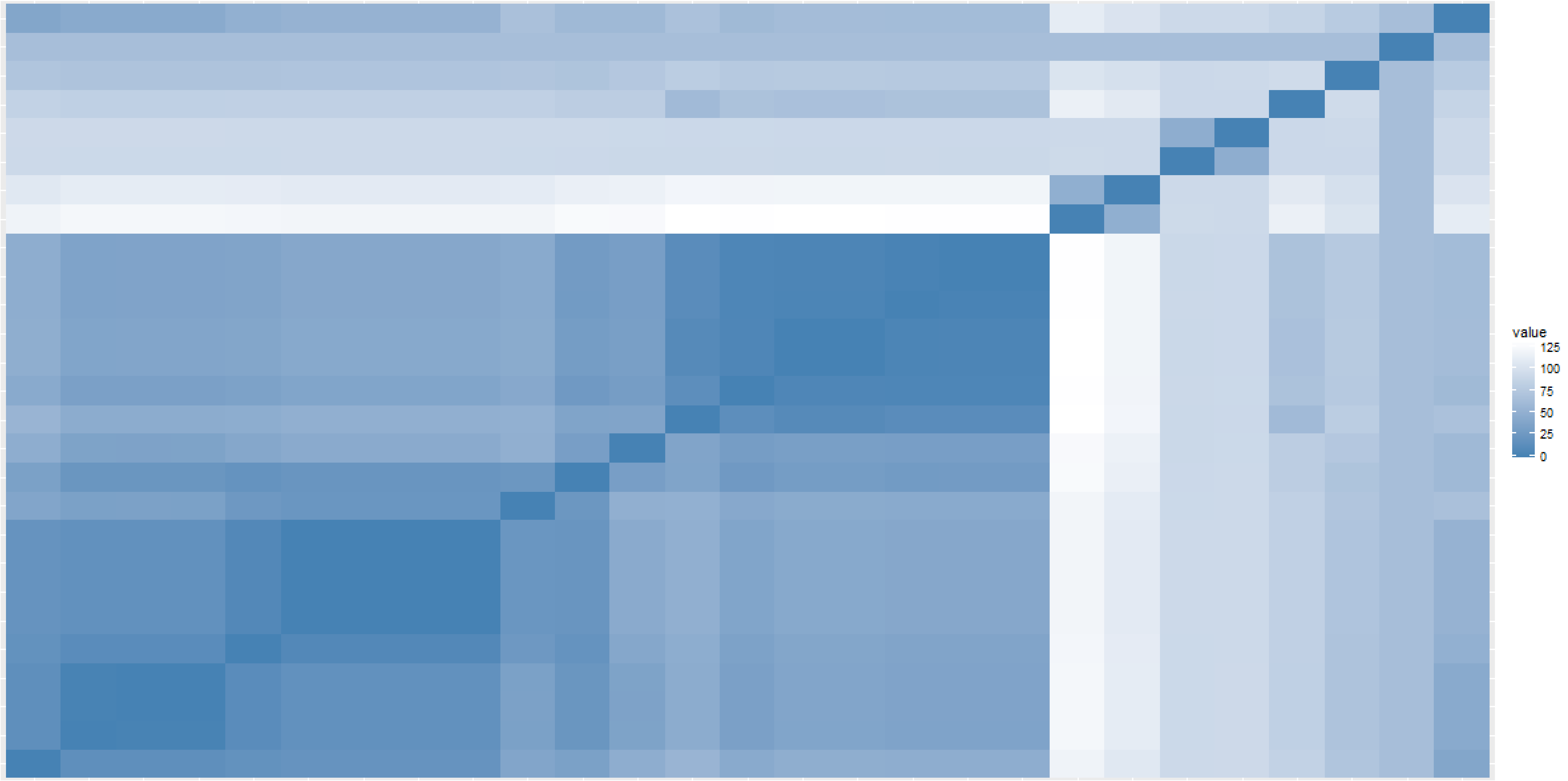

“Neighborhood_transferred”

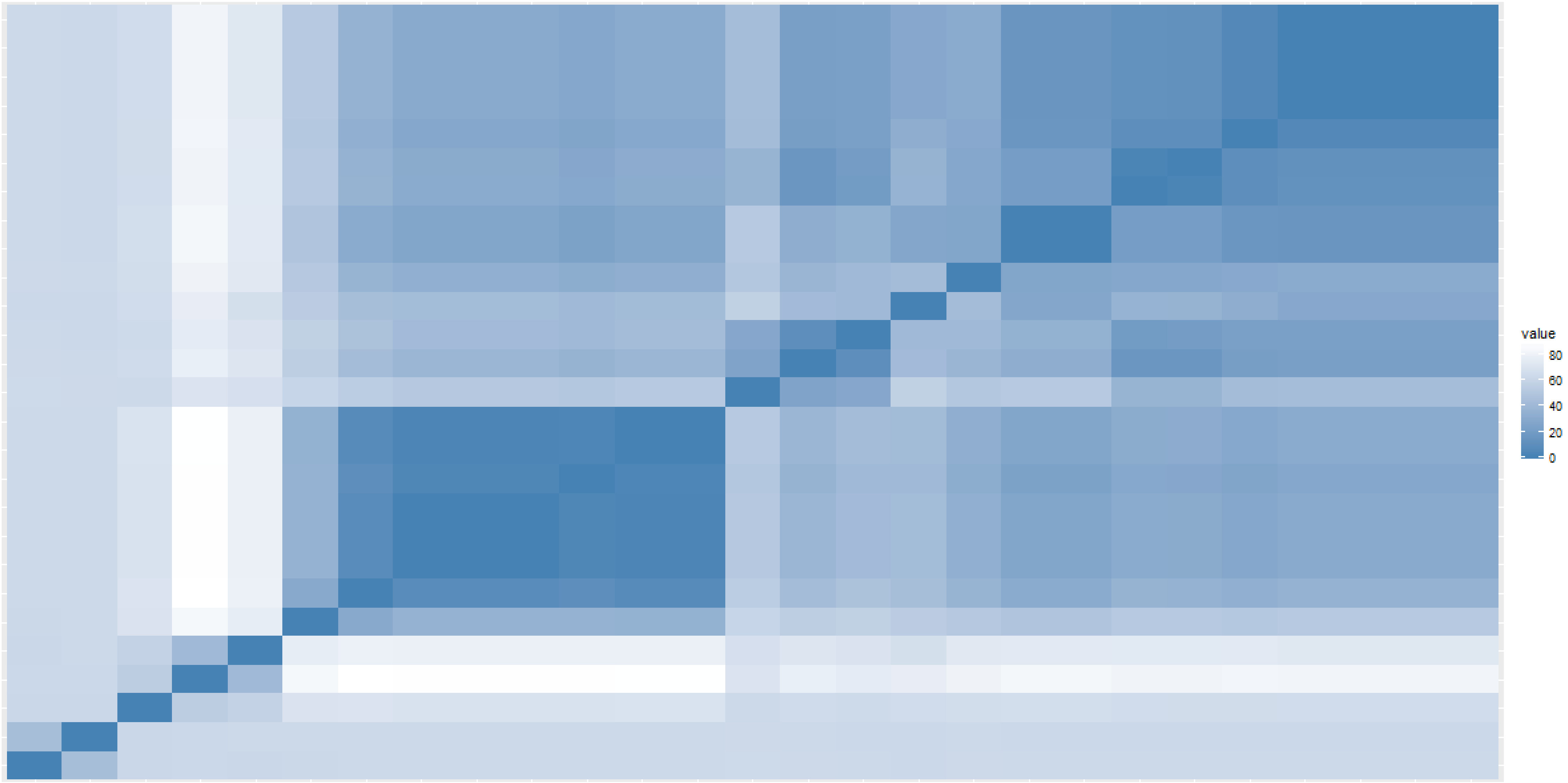

“Fusion”

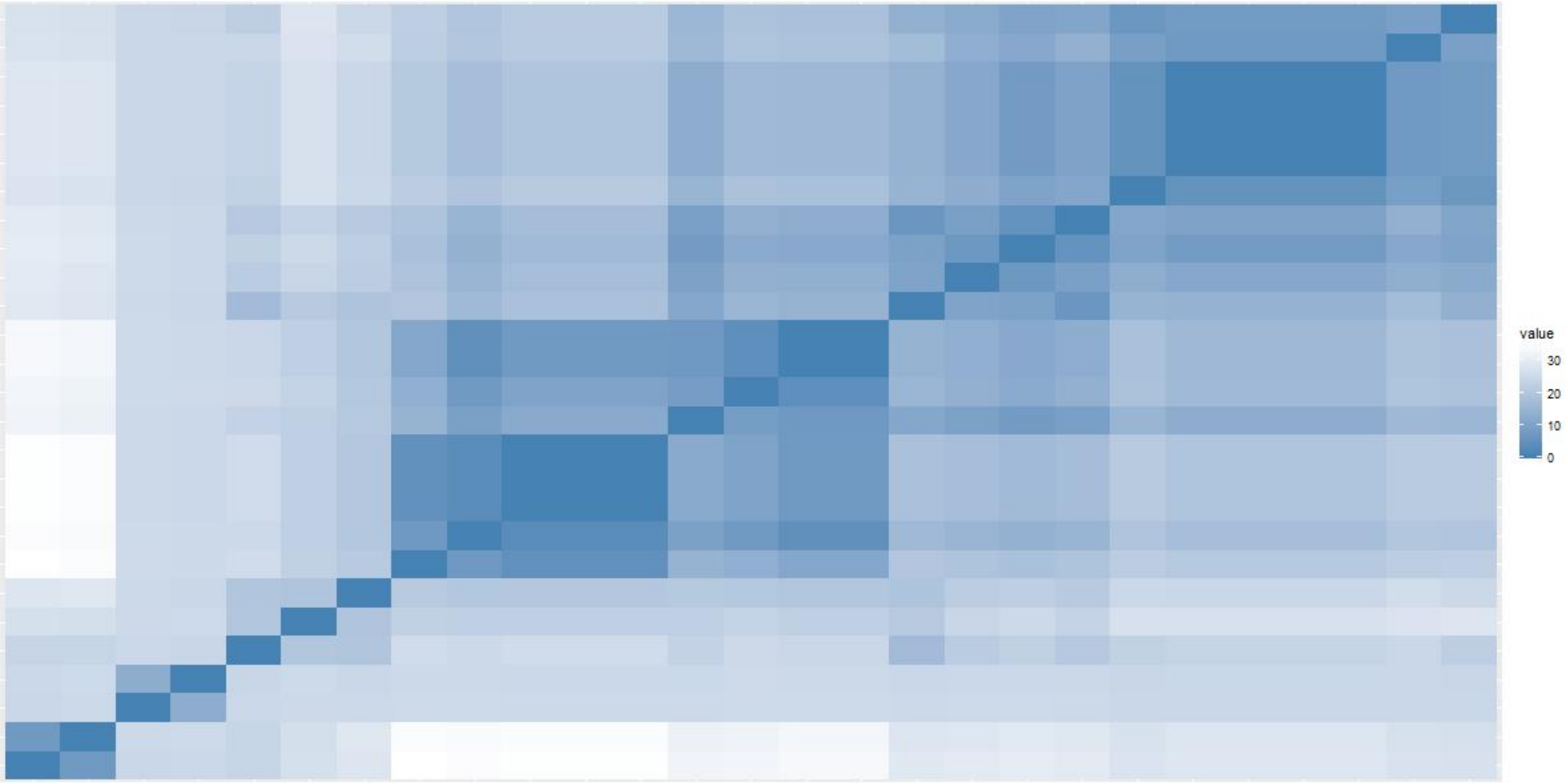

“Combined_score”

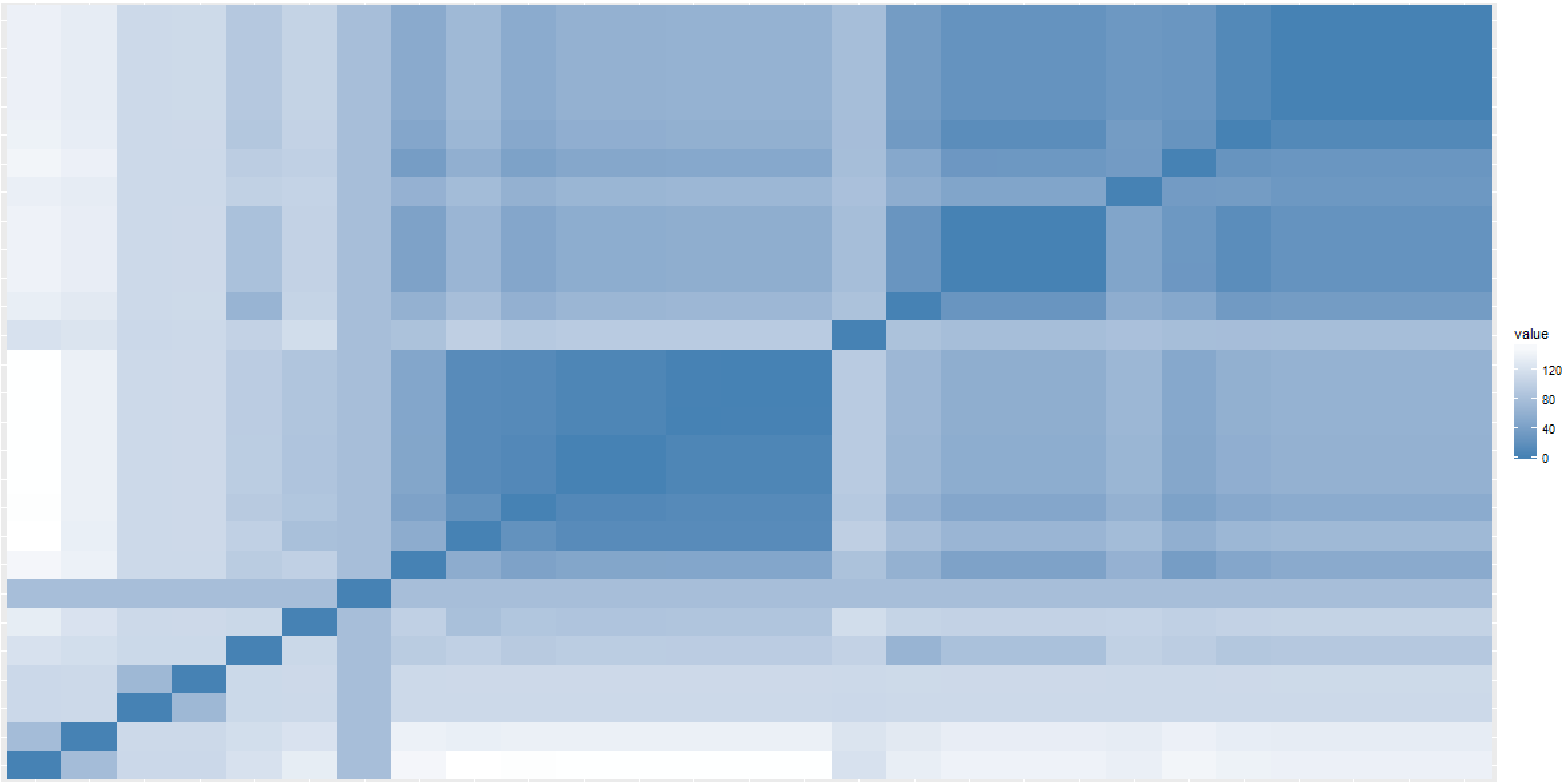

“Homology”

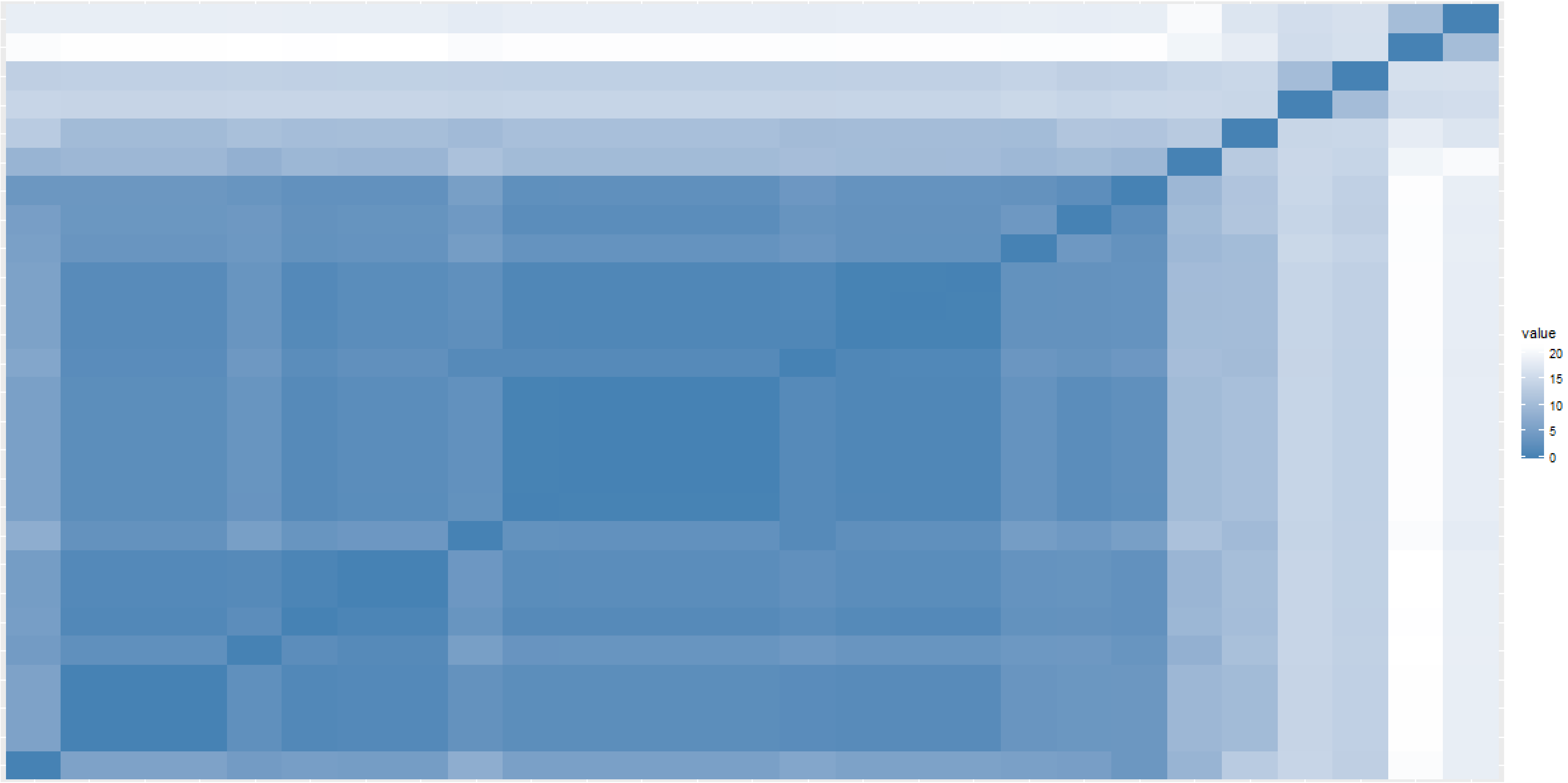

Supplement: Supplementary file 5 — Visual assessment of cluster tendency plots. Each rectangular represents the clusters of the calculated results of the centrality measures. (PDF 313 kb) [file 12918_2018_598_MOESM5_ESM.pdf]
