# Supplementary material for: A systematic survey of centrality measures for protein-protein interaction networks
Source: BMC Syst Biol. 2018 Jul 31;12:80. doi: 10.1186/s12918-018-0598-2 (PMC6069823; doi:10.1186/s12918-018-0598-2)
Supplement: Supplementary file 6 — Clustering properties results. These properties include connectivity, Dunn and Silhouette scores. These scores suggest the sufficient clustering method by a specific number of clusters. (DOCX 16 kb) [file 12918_2018_598_MOESM6_ESM.docx]

| **Clusters** | **Method** | **Score** | **Property** | **Network** |
| --- | --- | --- | --- | --- |
| 2 | hierarchical | 4.4448 | Connectivity | coexpression |
| 4 | hierarchical | 1.0183 | Dunn |  |
| 3 | pam | 0.4989 | Silhouette |  |
| 2 | hierarchical | 5.6619 | Connectivity | coexpression_transferred |
| 4 | hierarchical | 1.0219 | Dunn |  |
| 6 | kmeans | 0.5610 | Silhouette |  |
| 2 | hierarchical | 6.6520 | Connectivity | combined_score |
| 7 | hierarchical | 0.8297 | Dunn |  |
| 2 | hierarchical | 0.4698 | Silhouette |  |
| 2 | pam | 4.5456 | Connectivity | cooccurence |
| 7 | hierarchical | 1.3448 | Dunn |  |
| 5 | kmeans | 0.5838 | Silhouette |  |
| 2 | hierarchical | 5.7242 | Connectivity | database |
| 3 | hierarchical | 0.9848 | Dunn |  |
| 7 | hierarchical | 0.5060 | Silhouette |  |
| 2 | hierarchical | 7.6587 | Connectivity | database_transferred |
| 4 | hierarchical | 0.9716 | Dunn |  |
| 6 | kmeans | 0.4971 | Silhouette |  |
| 2 | hierarchical | 4.5782 | Connectivity | experiments |
| 3 | hierarchical | 1.0047 | Dunn |  |
| 5 | pam | 0.5220 | Silhouette |  |
| 2 | Pam | 3.2000 | Connectivity | experiments_transferred |
| 2 | hierarchical | 0.9951 | Dunn |  |
| 5 | kmeans | 0.5305 | Silhouette |  |
| 2 | hierarchical | 4.1913 | Connectivity | fussion |
| 2 | hierarchical | 0.8475 | Dunn |  |
| 7 | hierarchical | 0.4849 | Silhouette |  |
| 2 | hierarchical | 3.8579 | Connectivity | homology |
| 7 | hierarchical | 1.0859 | Dunn |  |
| 2 | hierarchical | 0.6944 | Silhouette |  |
| 2 | pam | 4.3294 | Connectivity | neighborhood_transferred |
| 3 | hierarchical | 1.0275 | Dunn |  |
| 2 | hierarchical | 0.5195 | Silhouette |  |
| 2 | hierarchical | 4.1913 | Connectivity | textminig_transferred |
| 5 | hierarchical | 0.8927 | Dunn |  |
| 2 | hierarchical | 0.5499 | Silhouette |  |
| 2 | hierarchical | 3.8579 | Connectivity | textmining |
| 2 | hierarchical | 0.9514 | Dunn |  |
| 2 | hierarchical | 0.5086 | Silhouette |  |
