# Supplementary material for: A systematic survey of centrality measures for protein-protein interaction networks
Source: BMC Syst Biol. 2018 Jul 31;12:80. doi: 10.1186/s12918-018-0598-2 (PMC6069823; doi:10.1186/s12918-018-0598-2)
Supplement: Supplementary file 7 — Clusters silhouette plots. Each color represents a cluster and each bar with specific color indicates a centrality. (PDF 417 kb) [file 12918_2018_598_MOESM7_ESM.pdf]

“Coexpression”

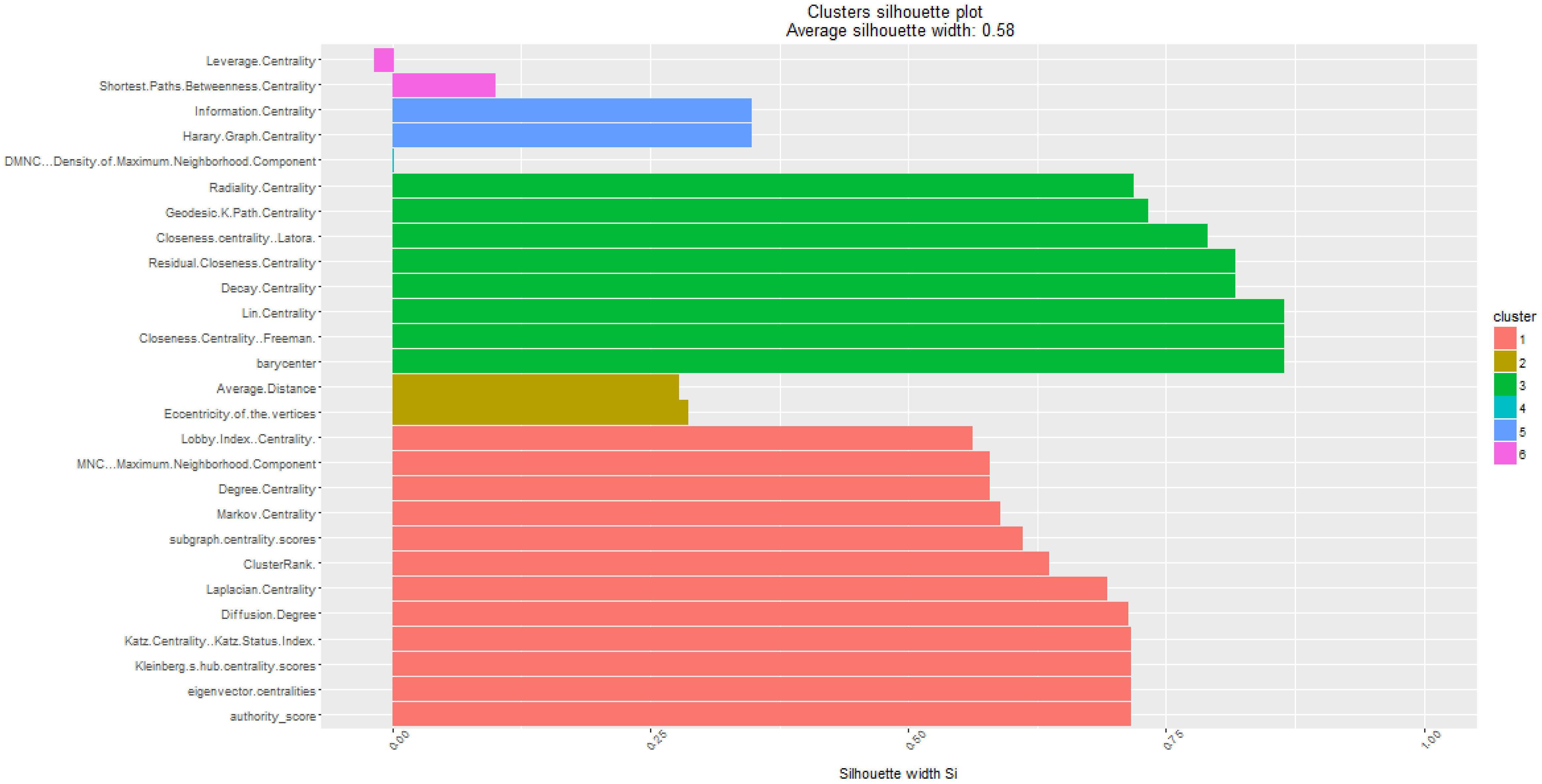

“Coexpression_transferred”

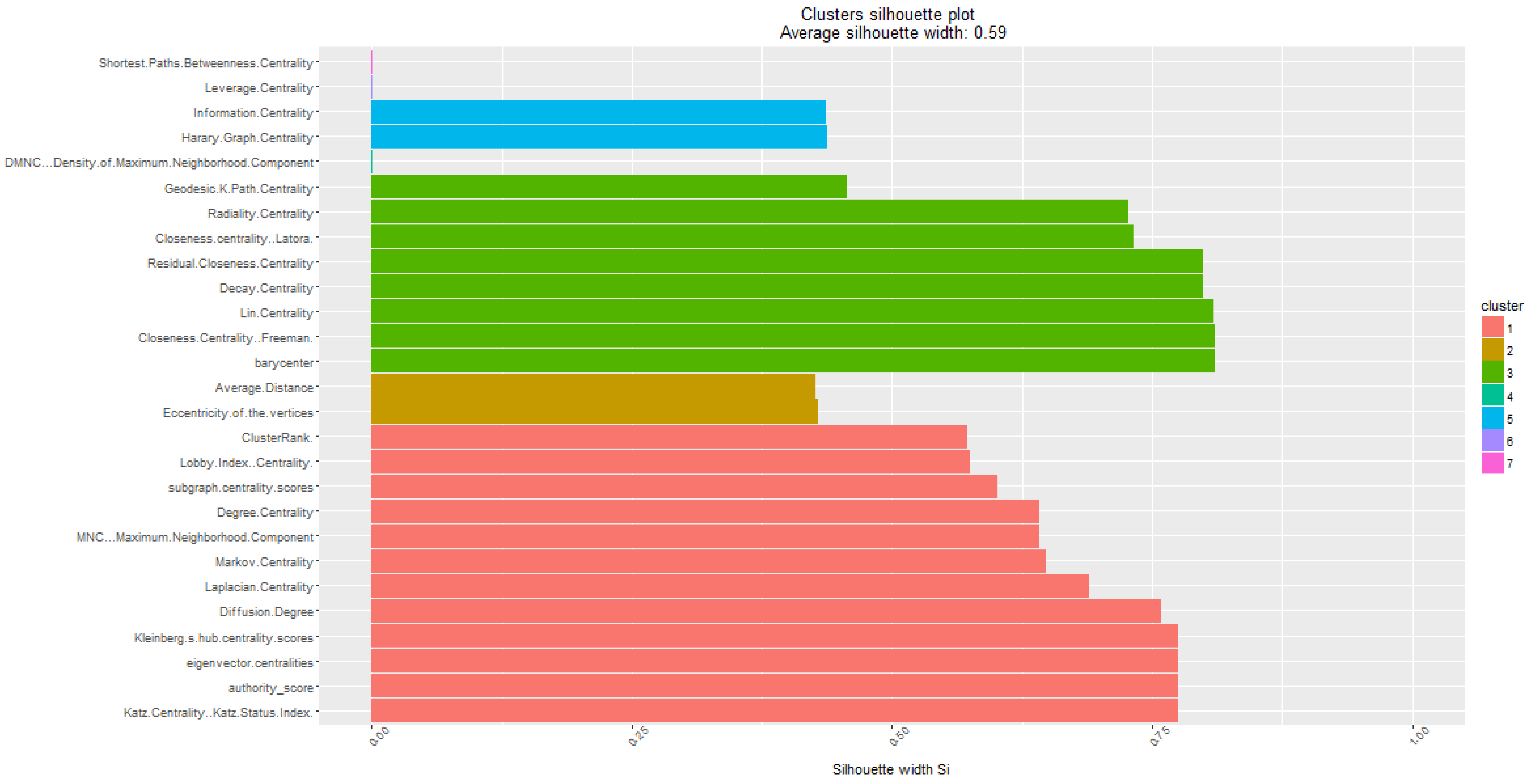

“Cooccurence”

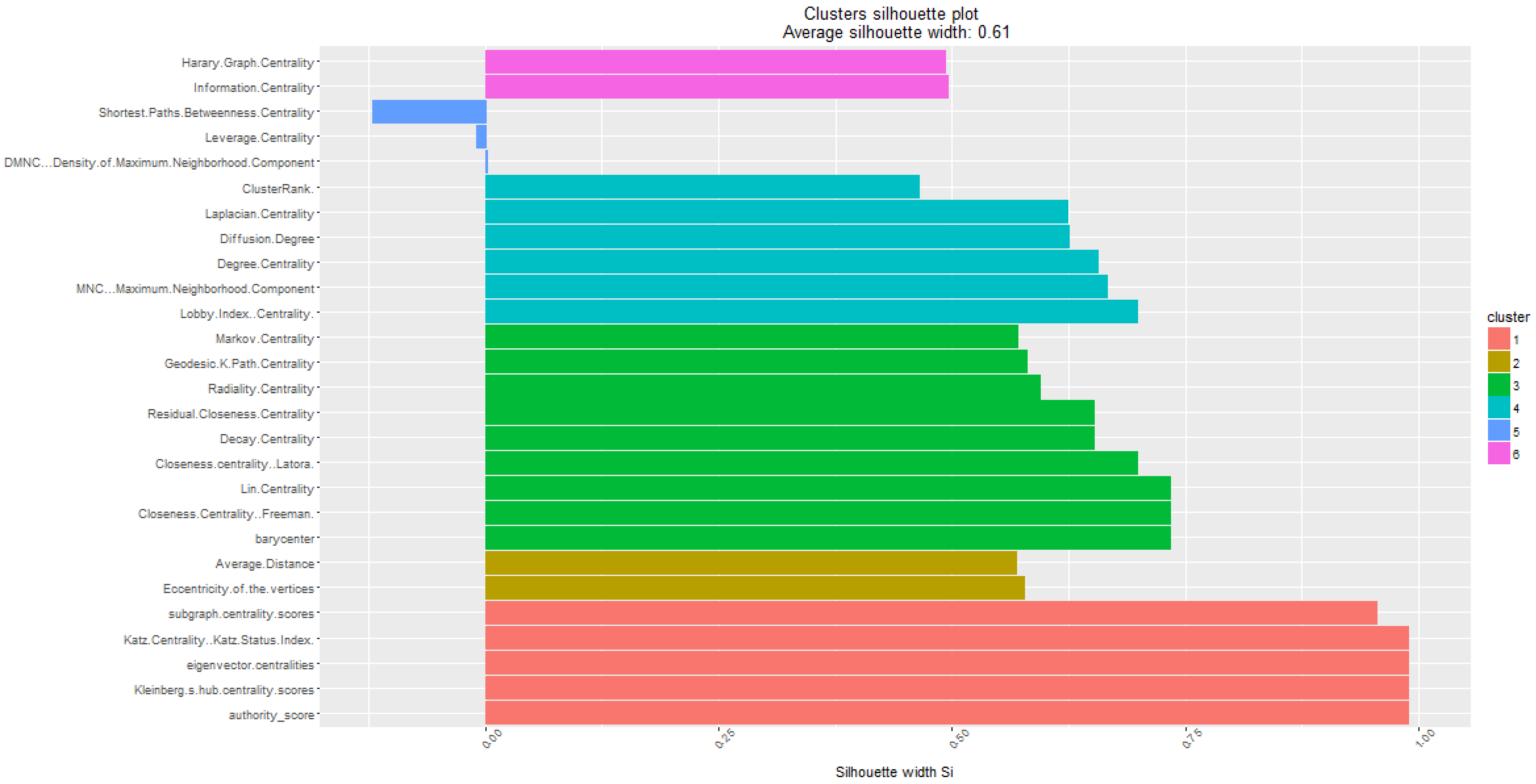

“Database”

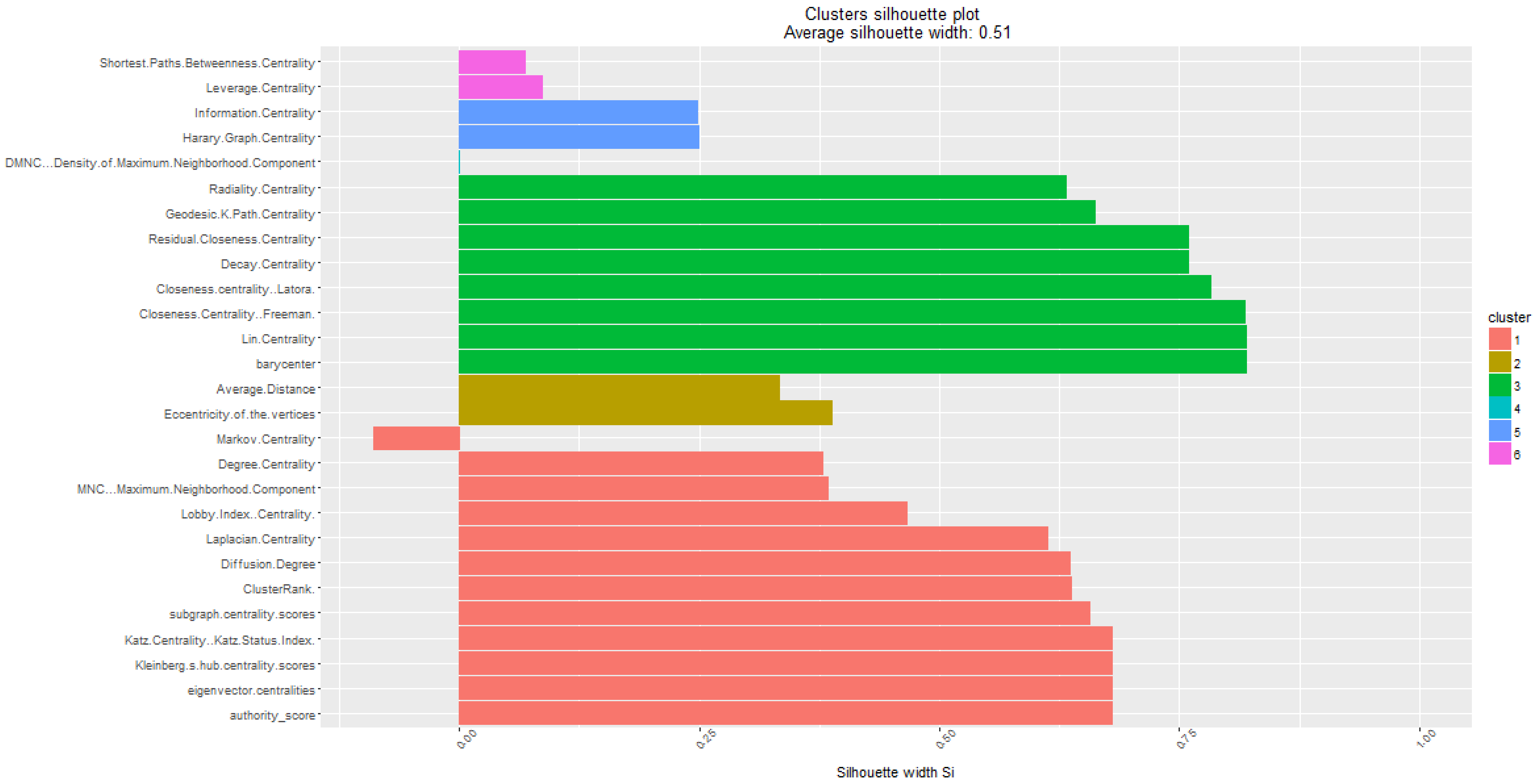
Clusters silhouette plot
Average silhouette width: 0.51
Shortest.Paths.Betweenness.Centrality
Leverage.Centrality
Information.Centrality
Harary.Graph.Centrality
DMNC...Density.of.Maximum.Neighborhood.Component
Radiality.Centrality
Geodesic.K.Path.Centrality
Residual.Closeness.Centrality
Decay.Centrality
Closeness.centrality..Latora.
Closeness.Centrality..Freeman.
Lin.Centrality
barycenter
Average.Distance
Eccentricity.of.the.vertices
Markov.Centrality
Degree.Centrality
MNC...Maximum.Neighborhood.Component
Lobby.Index..Centrality.
Laplacian.Centrality
Diffusion.Degree
ClusterRank.
subgraph.centrality.scores
Katz.Centrality..Katz.Status.Index.
Kleinberg.s.hub.centrality.scores
eigenvector.centralities
authority_score
0.00
0.25
0.50
0.75
1.00
Silhouette width Si
cluster
1
2
3
4
5
6

“Database_transferred”

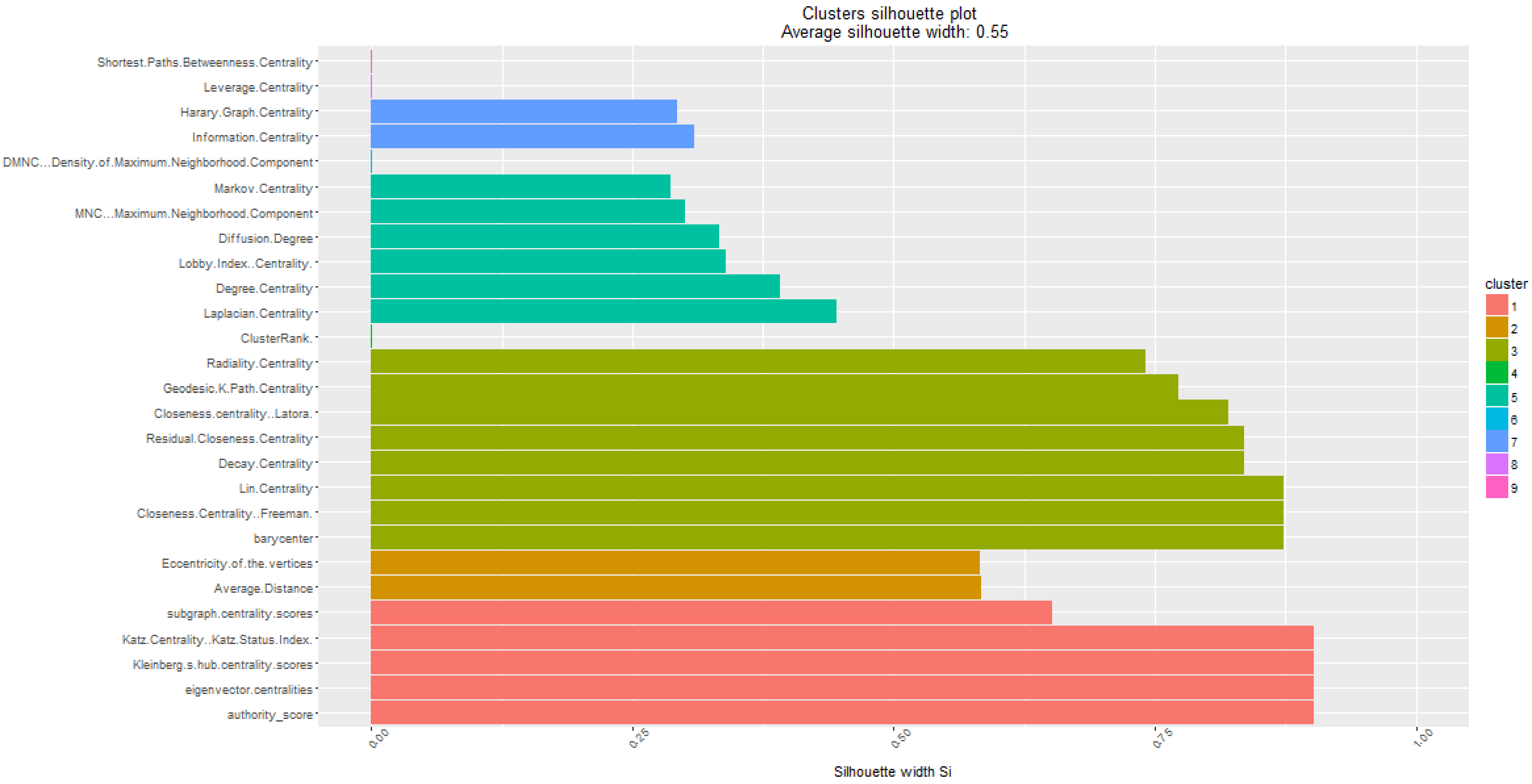

“Experiments”

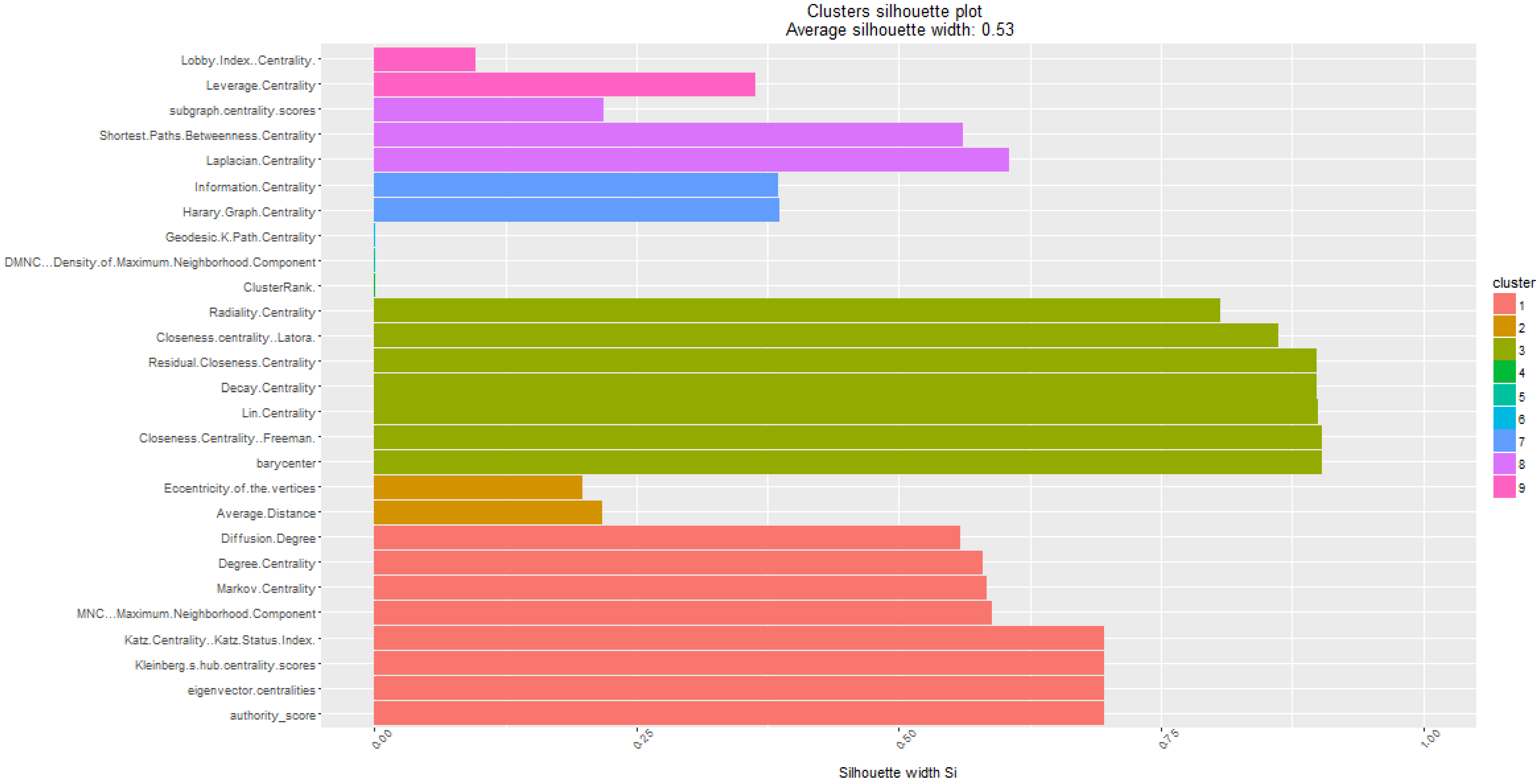

“Experiments_transferred”

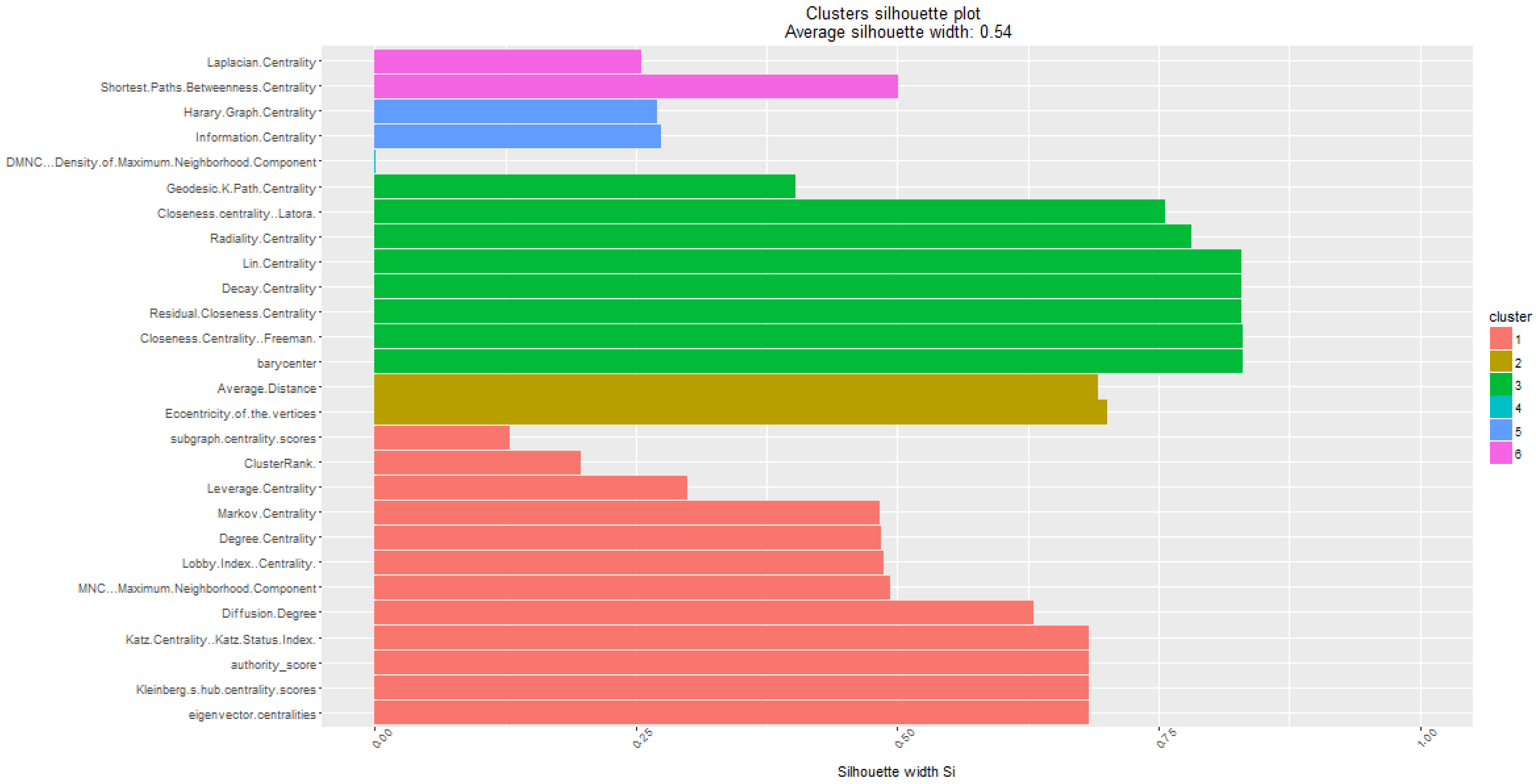

“Textmining”

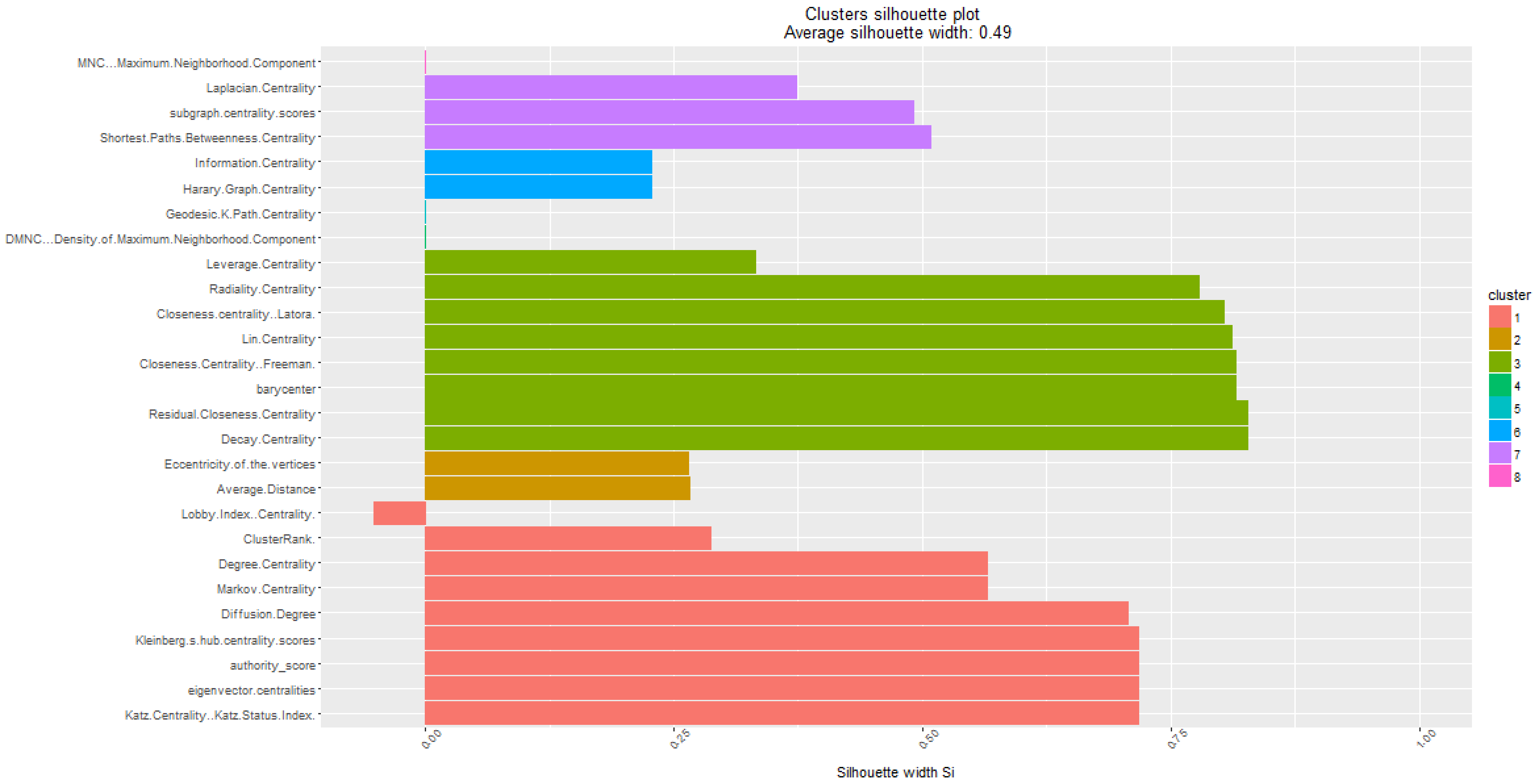

Clusters silhouette plot
Average silhouette width: 0.49
MNC...Maximum.Neighborhood.Component
Laplacian.Centrality
subgraph.centrality.scores
Shortest.Paths.Betweenness.Centrality
Information.Centrality
Harary.Graph.Centrality
Geodesic.K.Path.Centrality
DMNC...Density.of.Maximum.Neighborhood.Component
Leverage.Centrality
Radiality.Centrality
Closeness.centrality..Latora.
Lin.Centrality
Closeness.Centrality..Freeman.
barycenter
Residual.Closeness.Centrality
Decay.Centrality
Eccentricity.of.the.vertices
Average.Distance
Lobby.Index..Centrality.
ClusterRank.
Degree.Centrality
Markov.Centrality
Diffusion.Degree
Kleinberg.s.hub.centrality.scores
authority_score
eigenvector.centralities
Katz.Centrality..Katz.Status.Index.
0.00
0.25
0.50
0.75
1.00
Silhouette width Si
cluster
1
2
3
4
5
6
7
8

“Textmining_transferred”

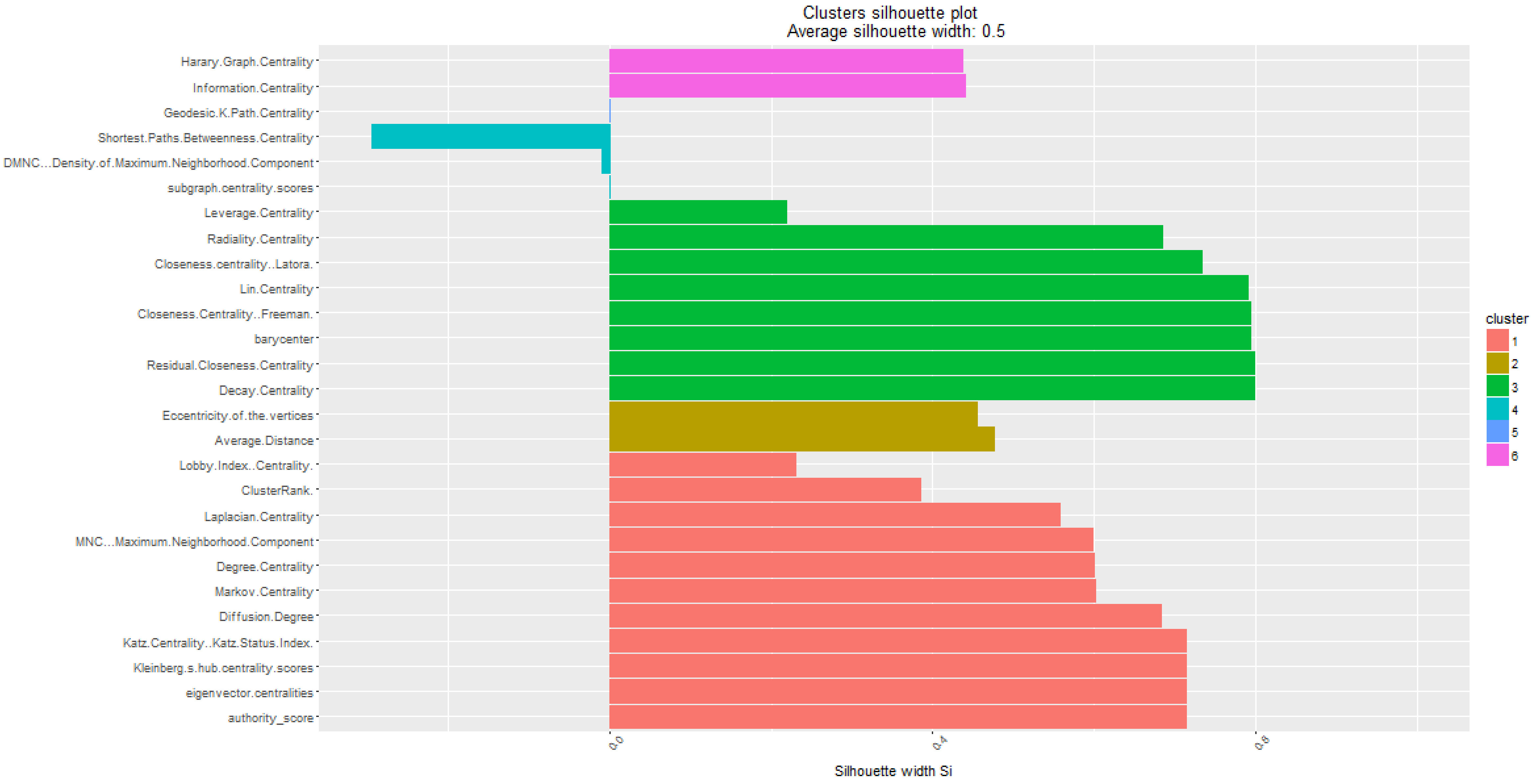

“Neighborhood_transferred”

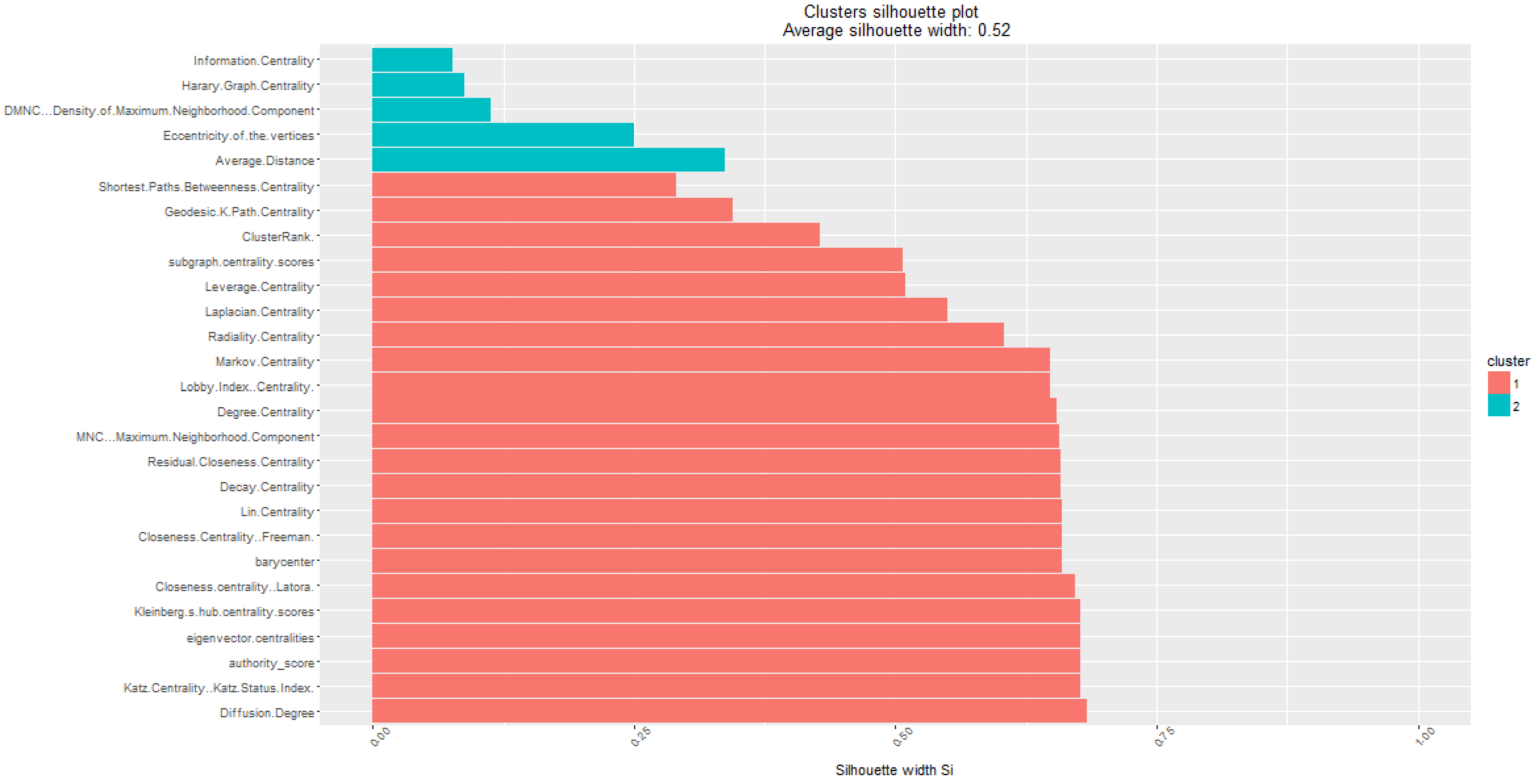

“Fusion”

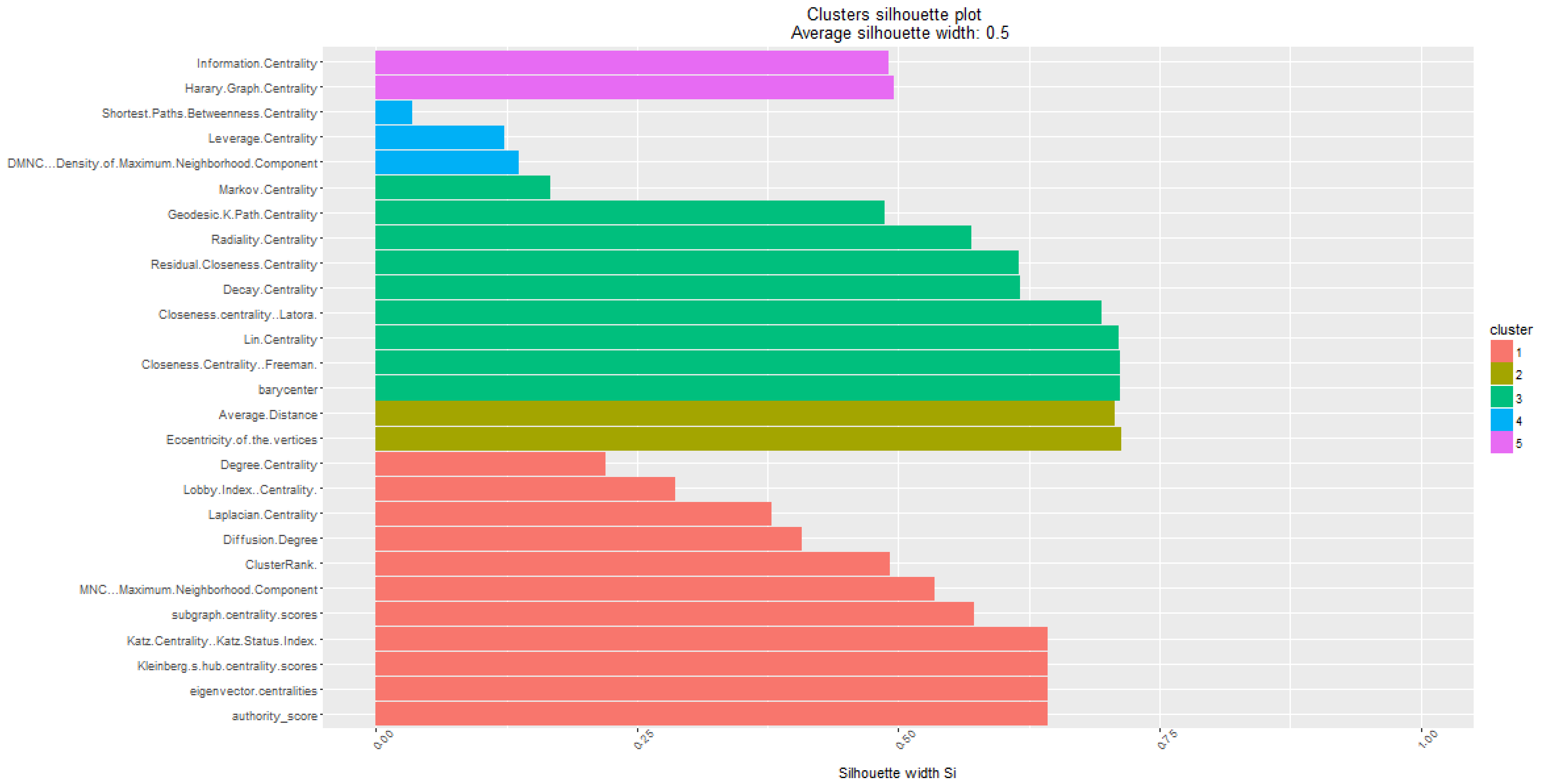

Clusters silhouette plot
Average silhouette width: 0.5
Information.Centrality
Harary.Graph.Centrality
Shortest.Paths.Betweenness.Centrality
Leverage.Centrality
DMNC...Density.of.Maximum.Neighborhood.Component
Markov.Centrality
Geodesic.K.Path.Centrality
Radiality.Centrality
Residual.Closeness.Centrality
Decay.Centrality
Closeness.centrality..Latora.
Lin.Centrality
Closeness.Centrality..Freeman.
barycenter
Average.Distance
Eccentricity.of.the.vertices
Degree.Centrality
Lobby.Index..Centrality.
Laplacian.Centrality
Diffusion.Degree
ClusterRank.
MNC...Maximum.Neighborhood.Component
subgraph.centrality.scores
Katz.Centrality..Katz.Status.Index.
Kleinberg.s.hub.centrality.scores
eigenvector.centralities
authority_score
0.00
0.25
0.50
0.75
1.00
Silhouette width Si
cluster
1
2
3
4
5

“Combined_score”

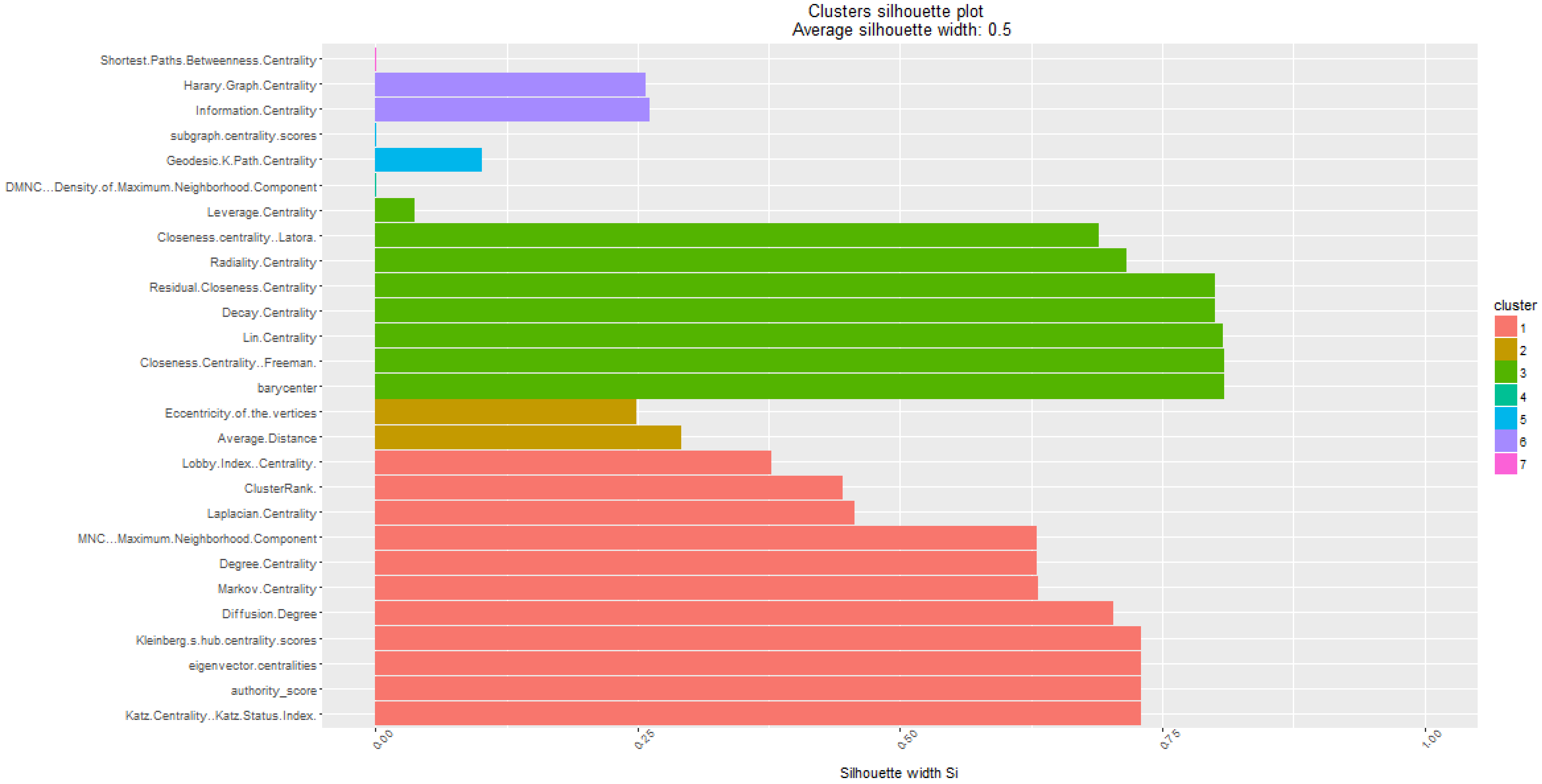

“Homology”

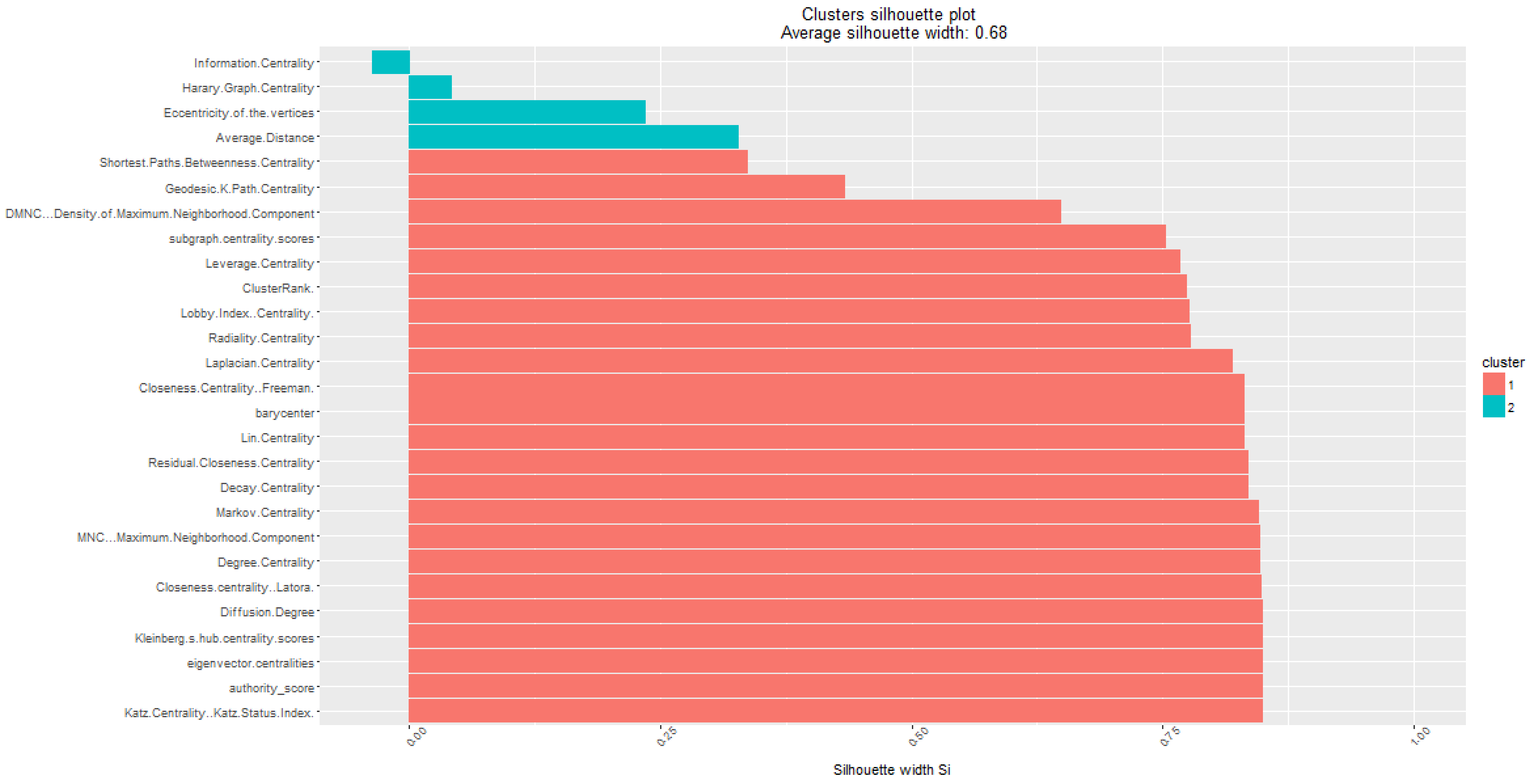

Clusters silhouette plot
Average silhouette width: 0.68
Information.Centrality
Harary.Graph.Centrality
Eccentricity.of.the.vertices
Average.Distance
Shortest.Paths.Betweenness.Centrality
Geodesic.K.Path.Centrality
DMNC...Density.of.Maximum.Neighborhood.Component
subgraph.centrality.scores
Leverage.Centrality
ClusterRank.
Lobby.Index..Centrality.
Radiality.Centrality
Laplacian.Centrality
Closeness.Centrality..Freeman.
barycenter
Lin.Centrality
Residual.Closeness.Centrality
Decay.Centrality
Markov.Centrality
MNC...Maximum.Neighborhood.Component
Degree.Centrality
Closeness.centrality..Latora.
Diffusion.Degree
Kleinberg.s.hub.centrality.scores
eigenvector.centralities
authority_score
Katz.Centrality..Katz.Status.Index.
0.00
0.25
0.50
0.75
1.00
Silhouette width Si
cluster
1
2
