# Supplementary material for: A systematic survey of centrality measures for protein-protein interaction networks
Source: BMC Syst Biol. 2018 Jul 31;12:80. doi: 10.1186/s12918-018-0598-2 (PMC6069823; doi:10.1186/s12918-018-0598-2)
Supplement: Supplementary file 8 — Optimal number of clusters. The suitable number of clusters for hierarchical clustering method was computed using the average silhouette values. (PDF 321 kb) [file 12918_2018_598_MOESM8_ESM.pdf]

“Coexpression”

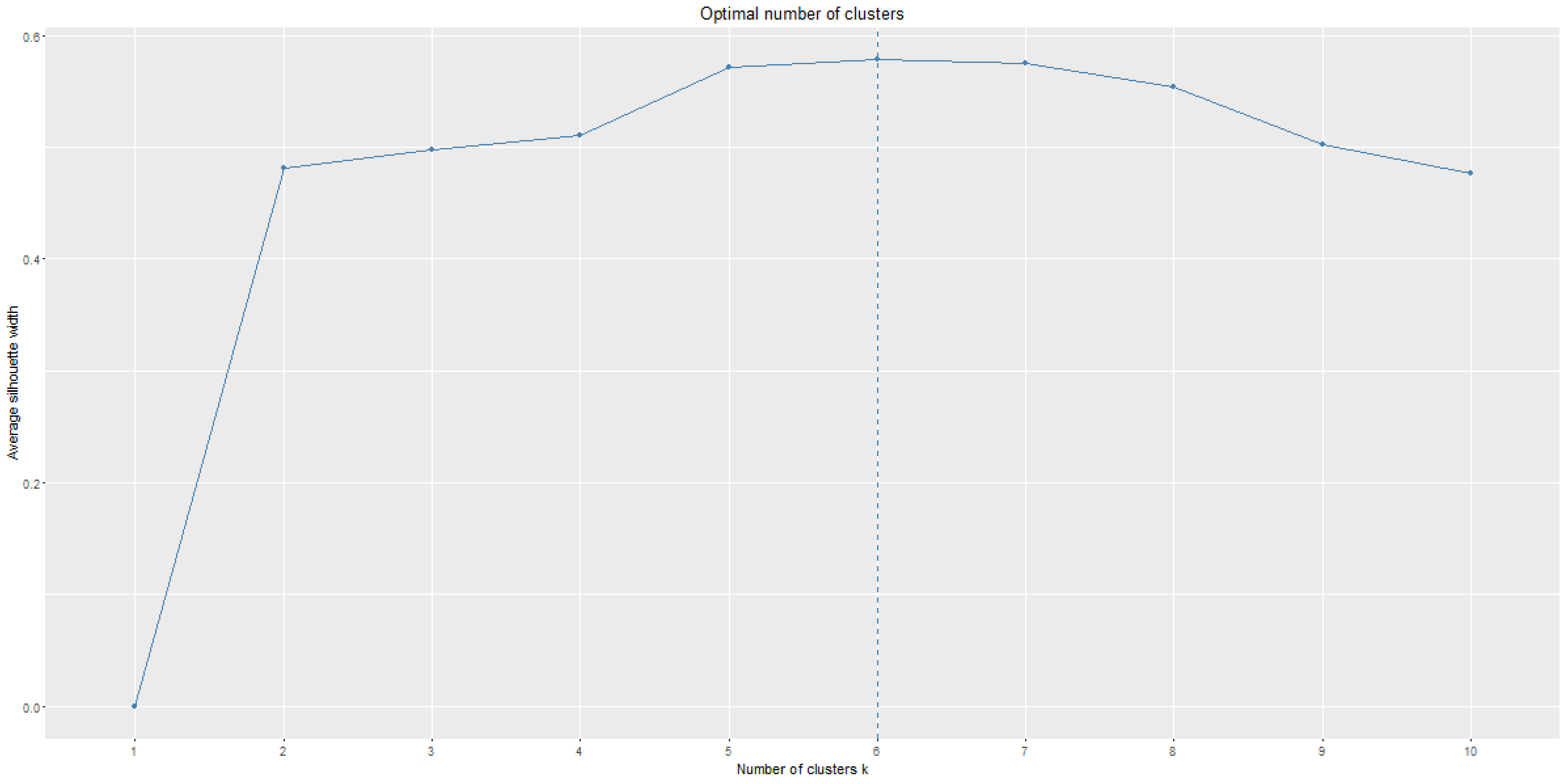

“Coexpression_transferred”

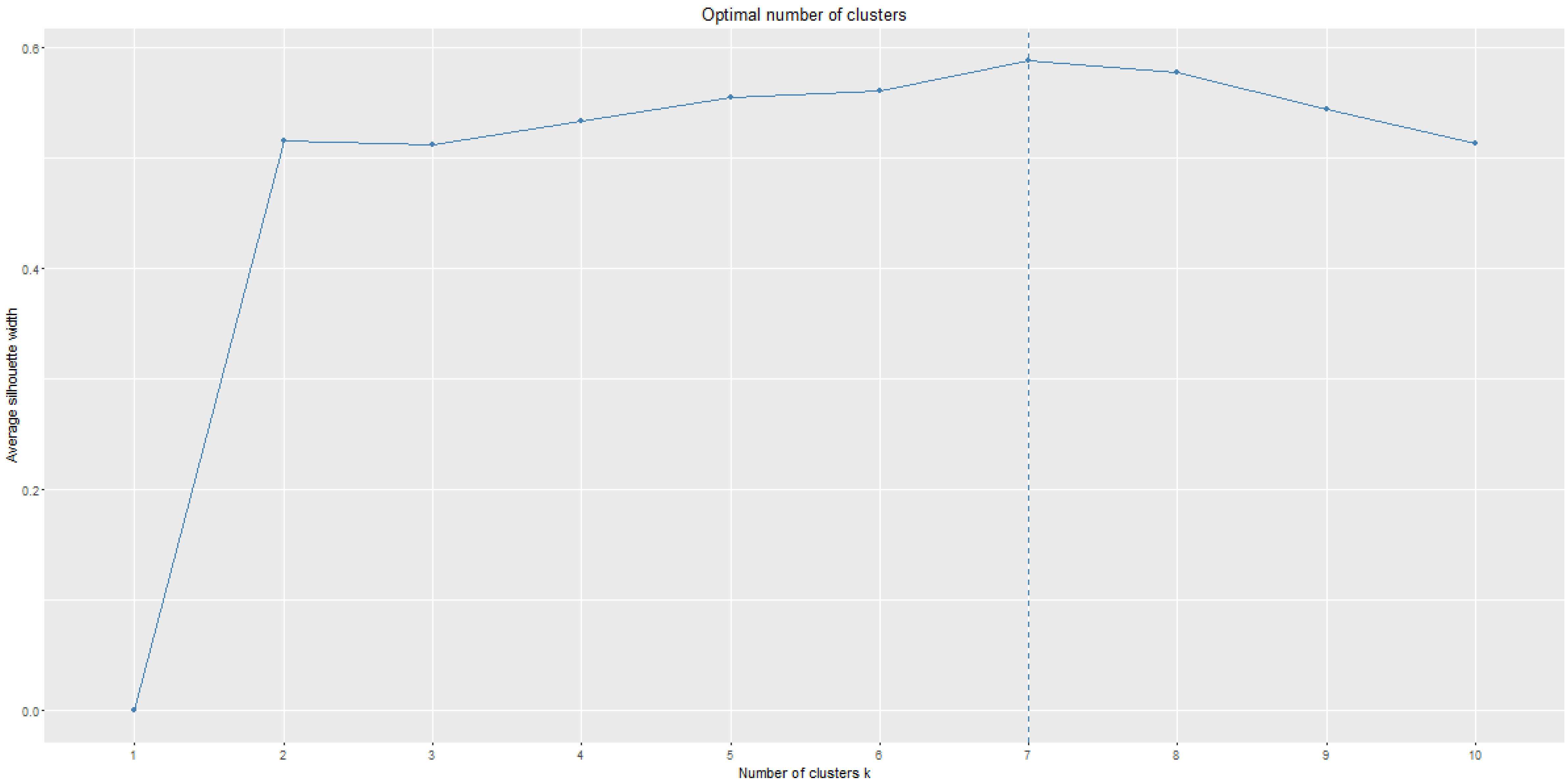

“Cooccurence”

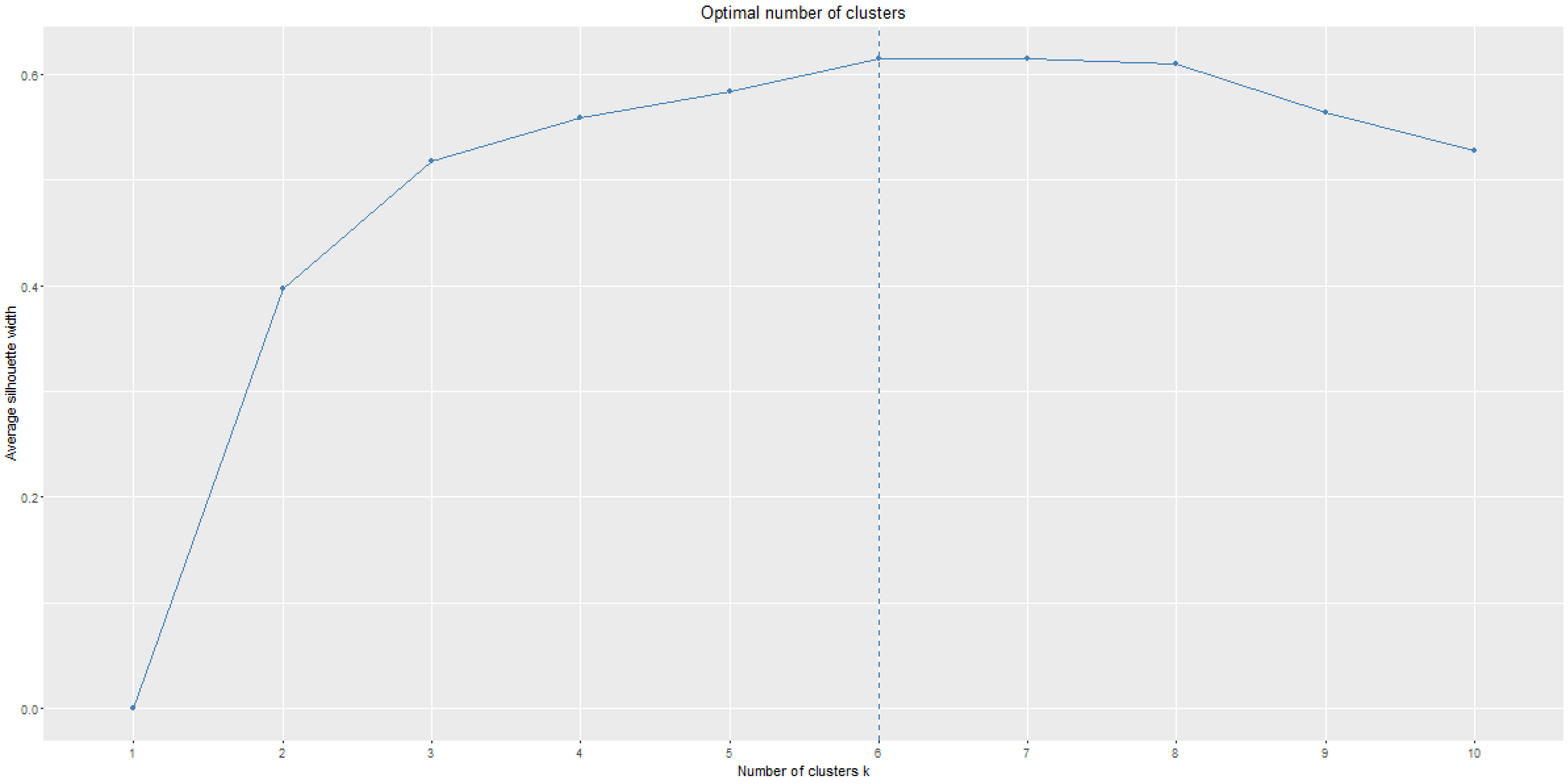

“Database”

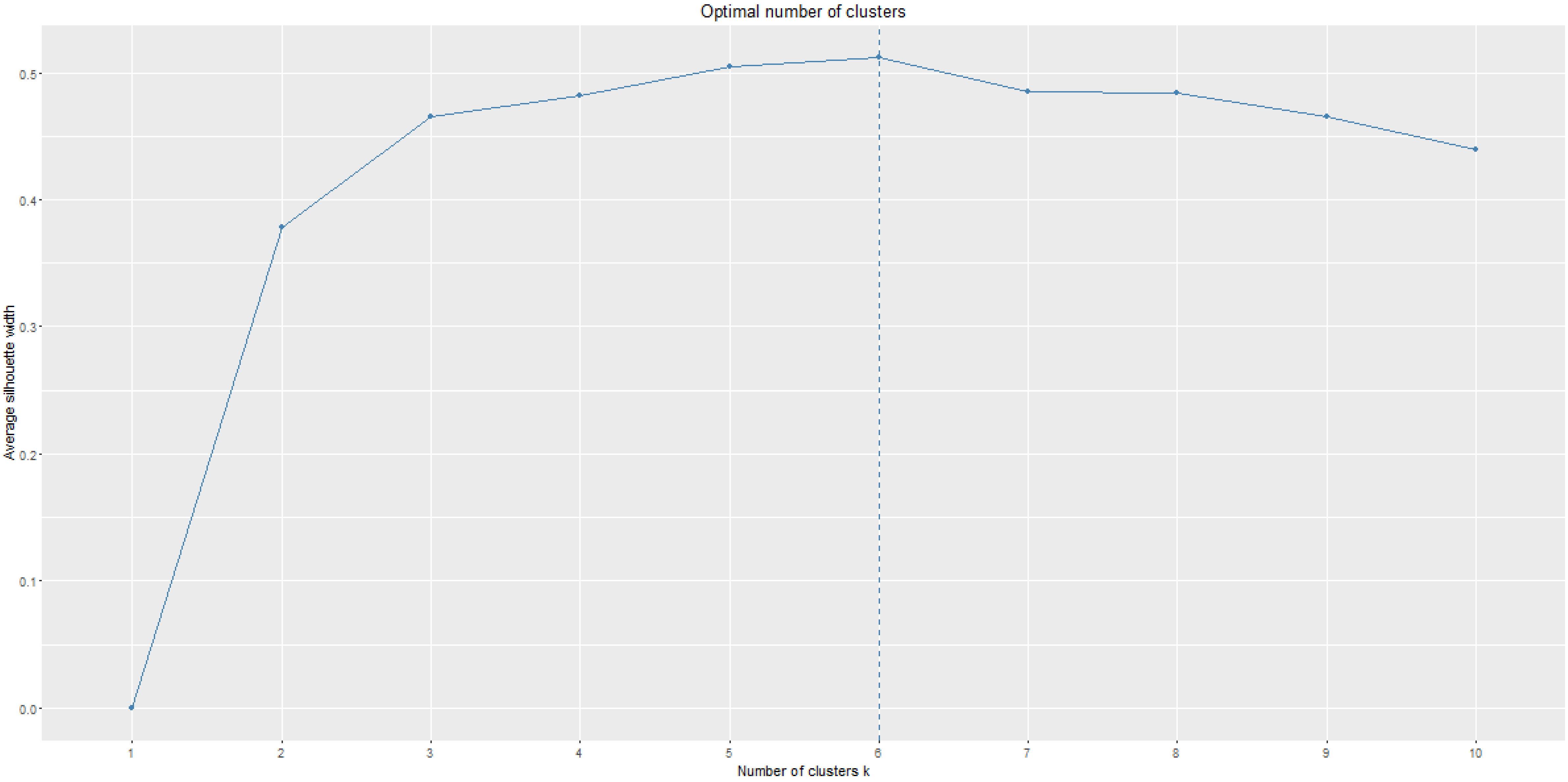

Optimal number of clusters
Average silhouette width
0.0
0.1
0.2
0.3
0.4
0.5
1
2
3
4
5
6
7
8
9
10
Number of clusters k

“Database_transferred”

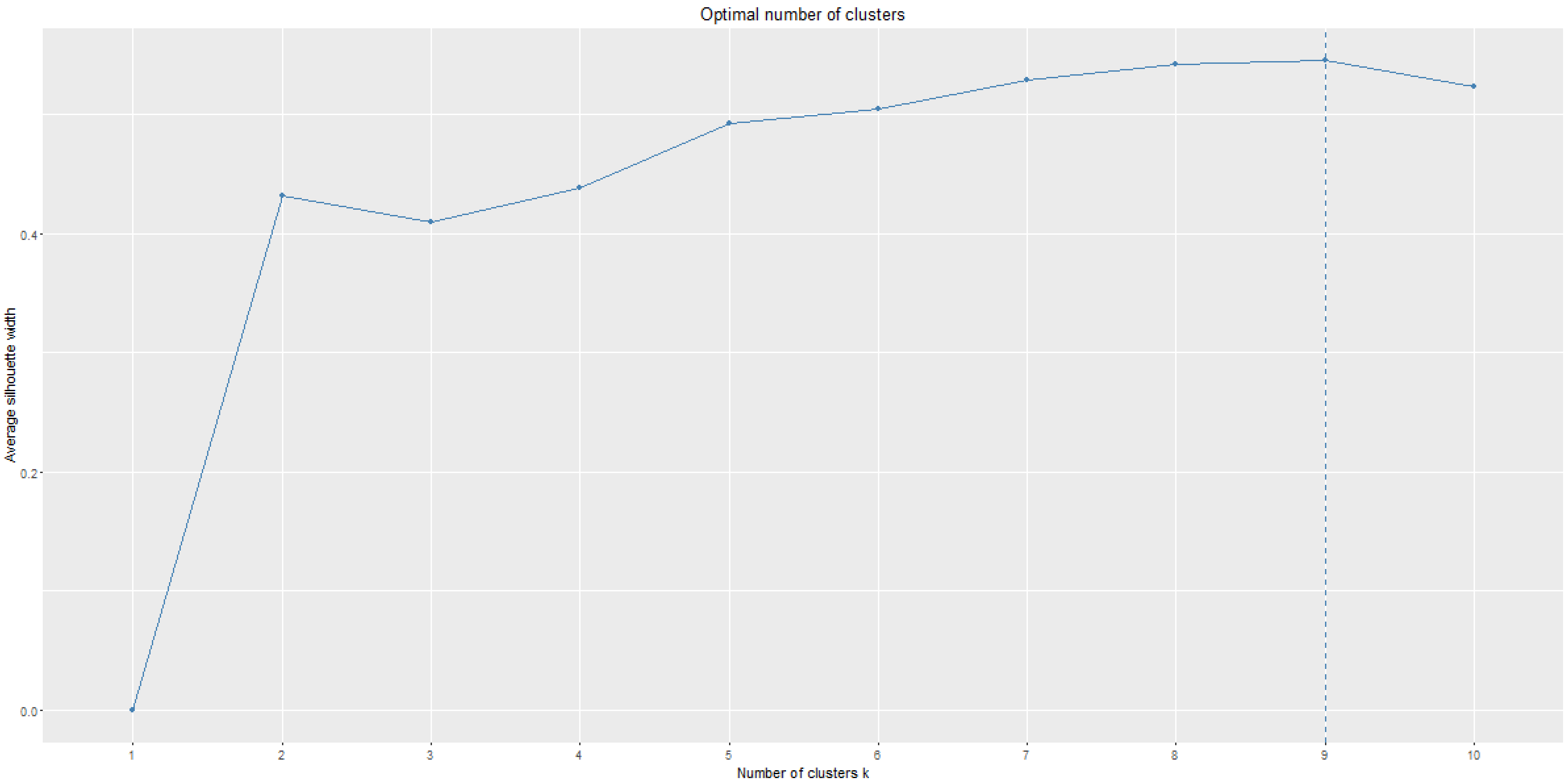

“Experiments”

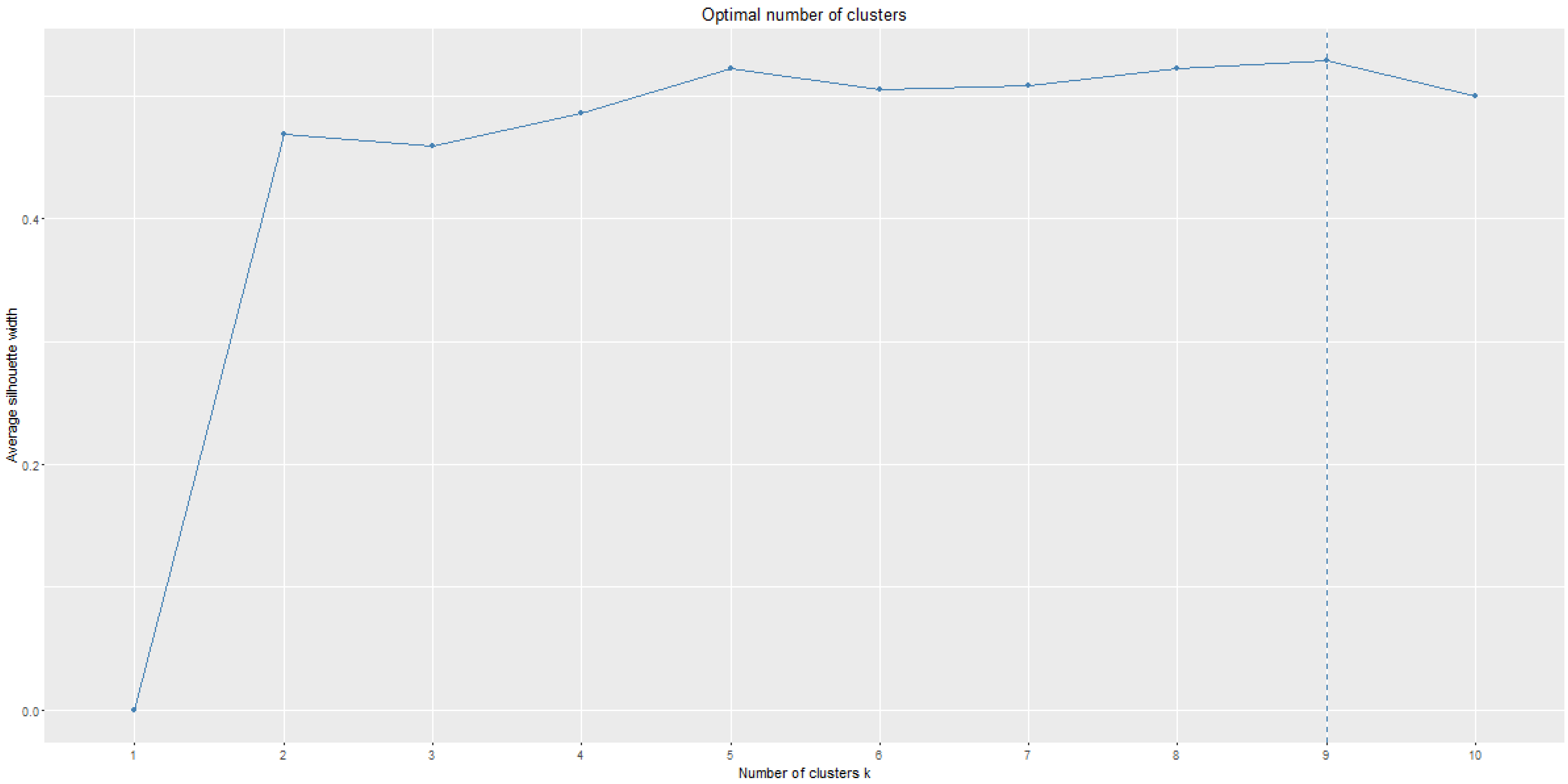

“Experiments_transferred”

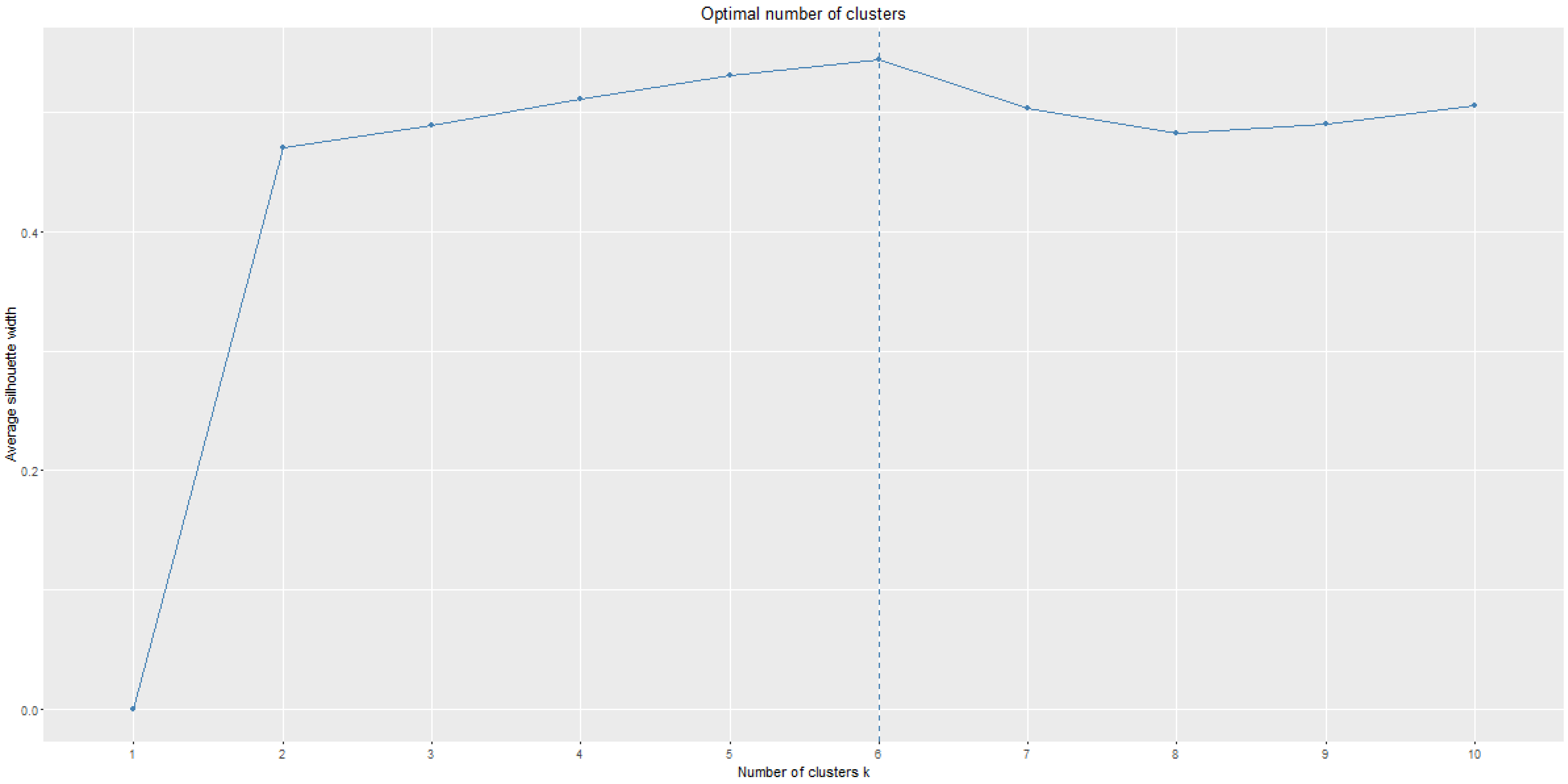

“Textmining”

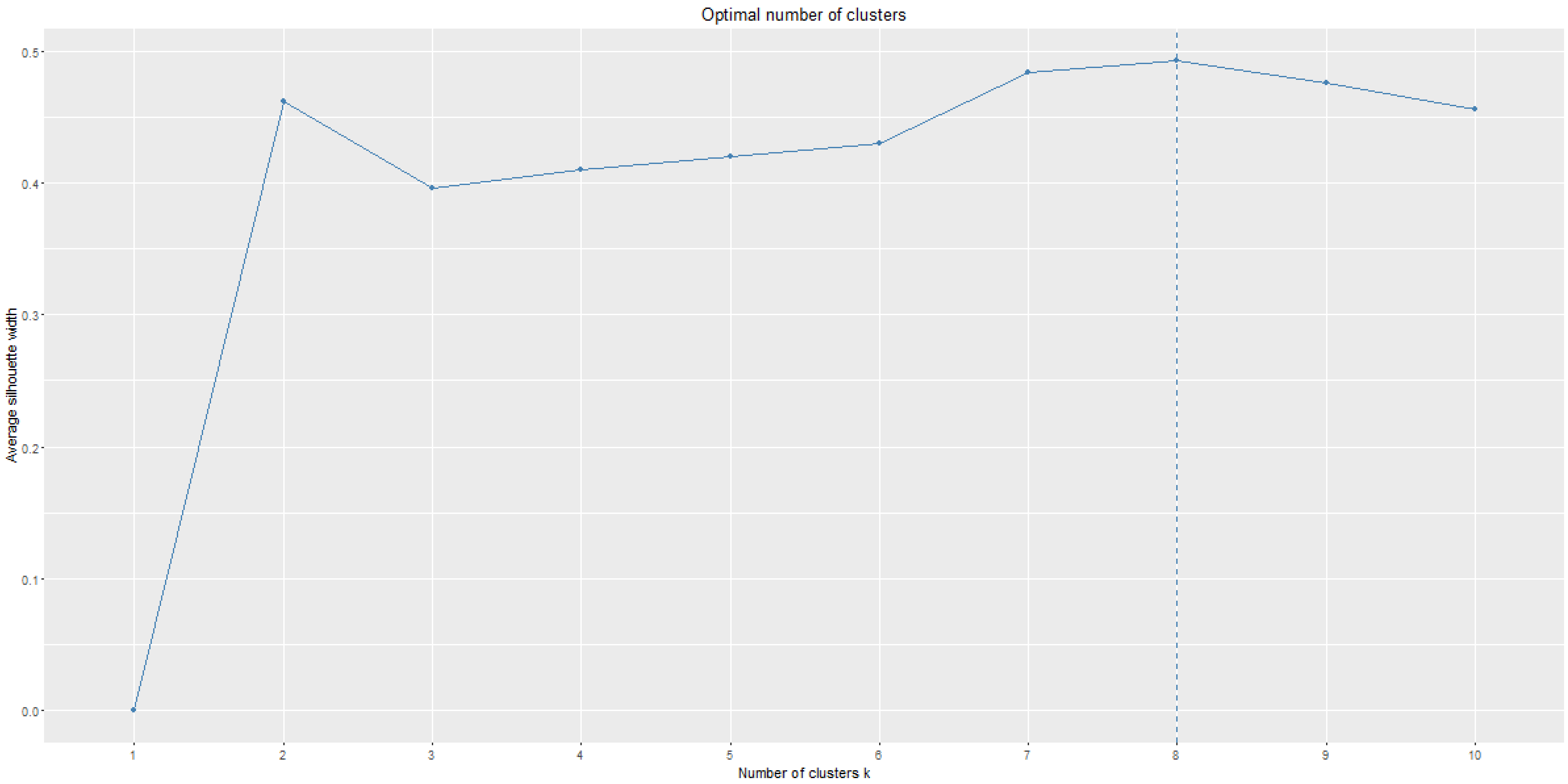

Optimal number of clusters
Average silhouette width
0.0
0.1
0.2
0.3
0.4
0.5
1
2
3
4
5
6
7
8
9
10
Number of clusters k

“Textmining_transferred”

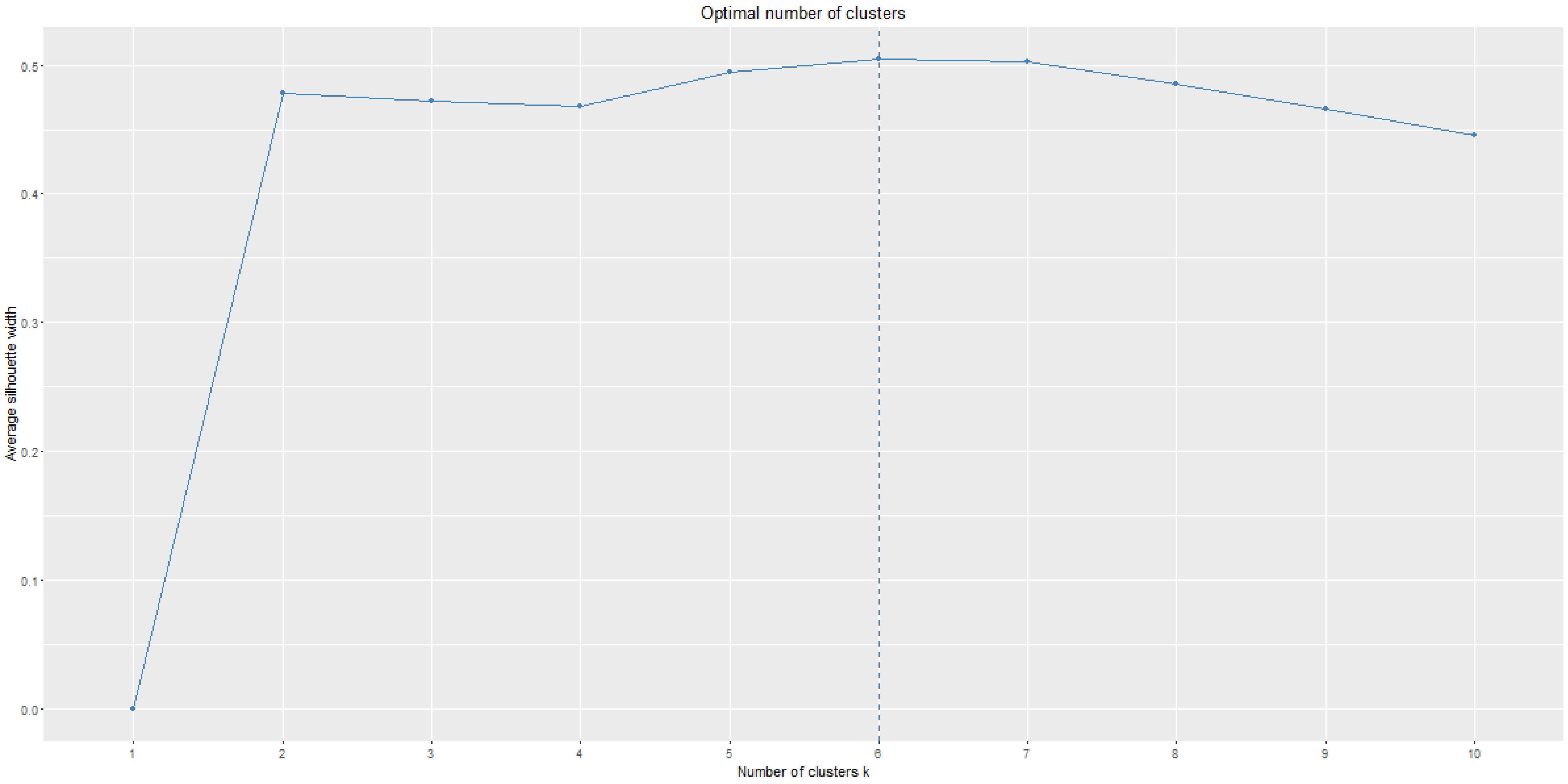

“Neighborhood_transferred”

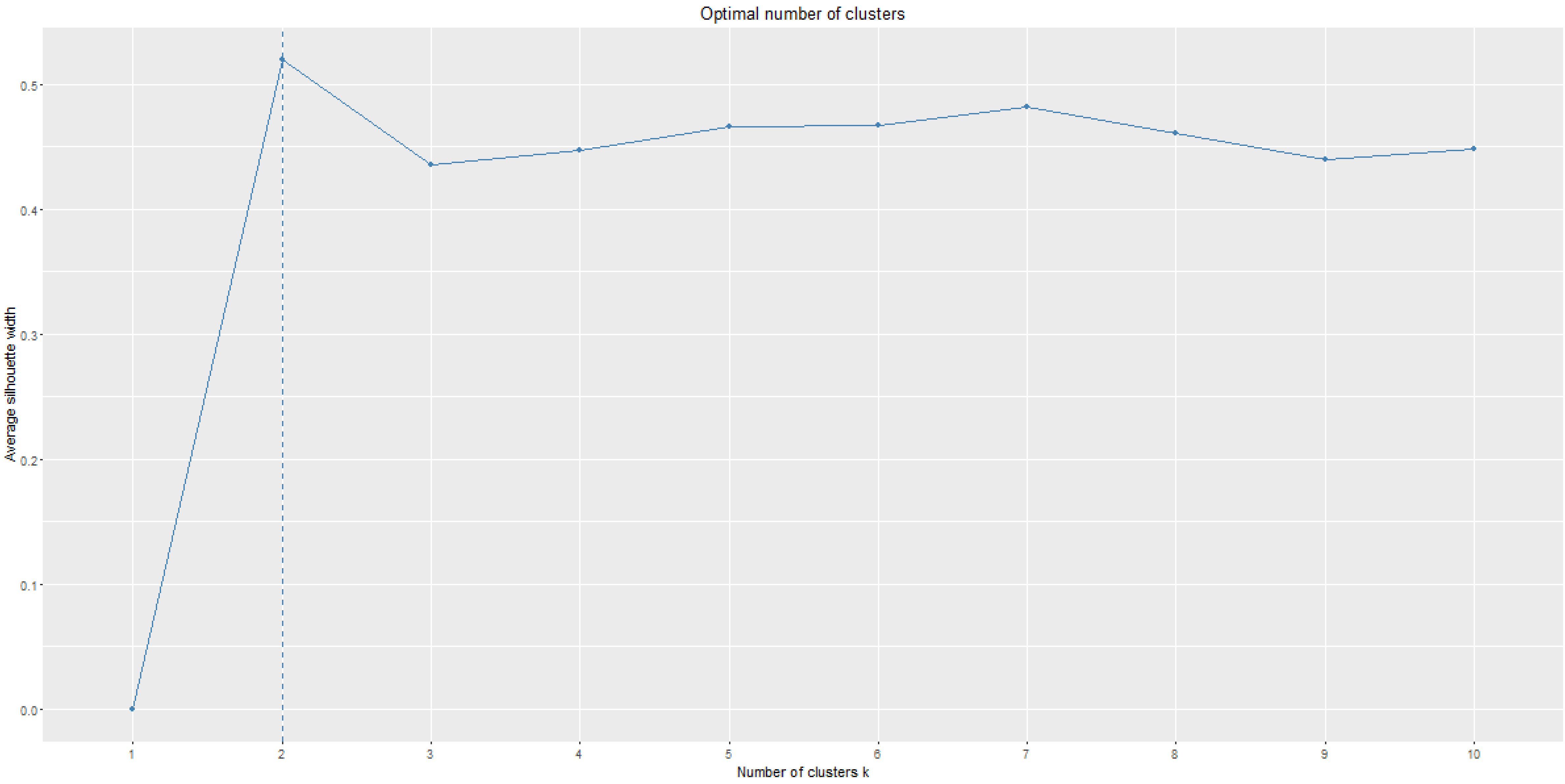

“Fusion”

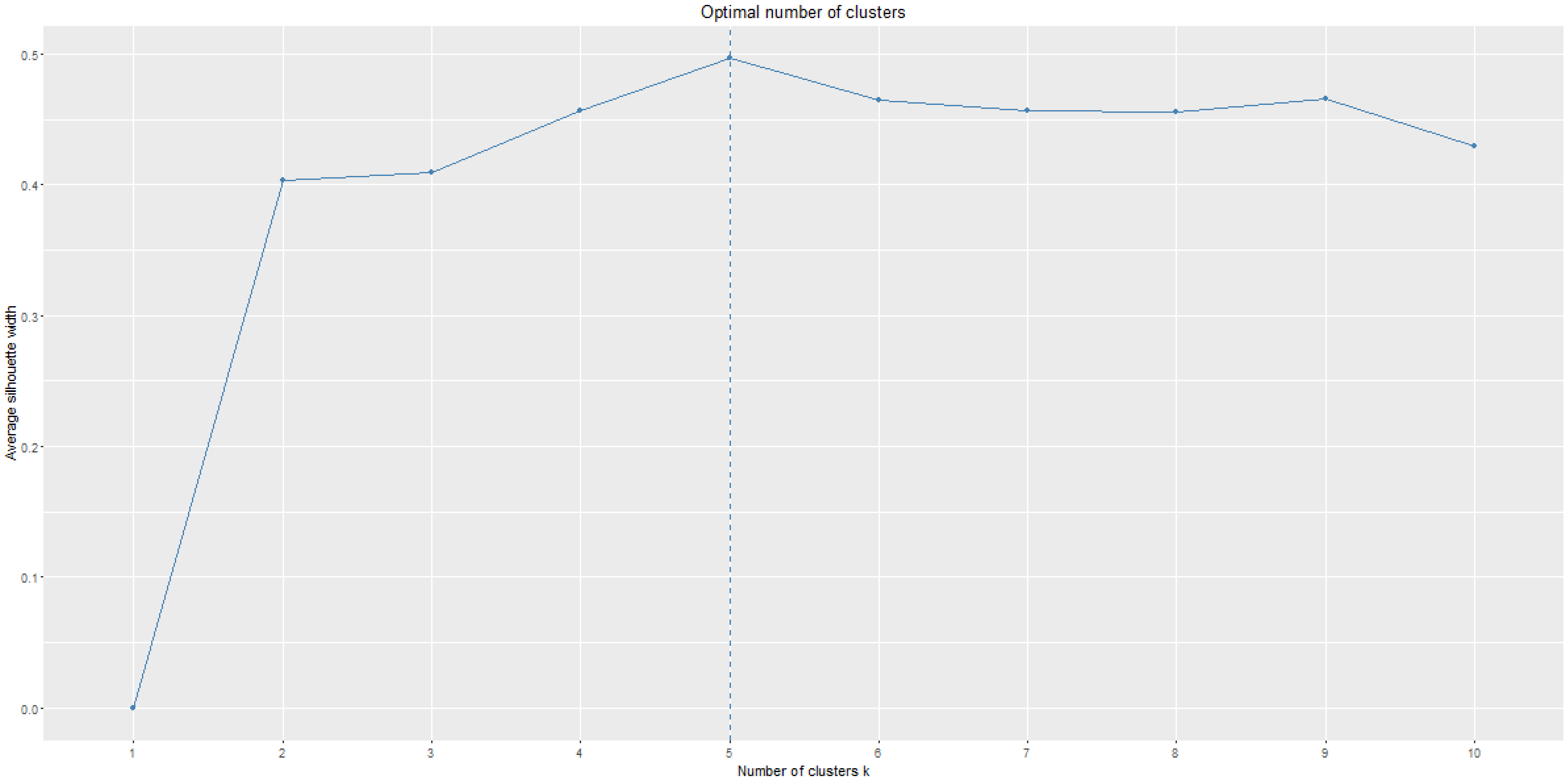

Optimal number of clusters
Average silhouette width
0.0
0.1
0.2
0.3
0.4
0.5
1
2
3
4
5
6
7
8
9
10
Number of clusters k

“Combined_score”

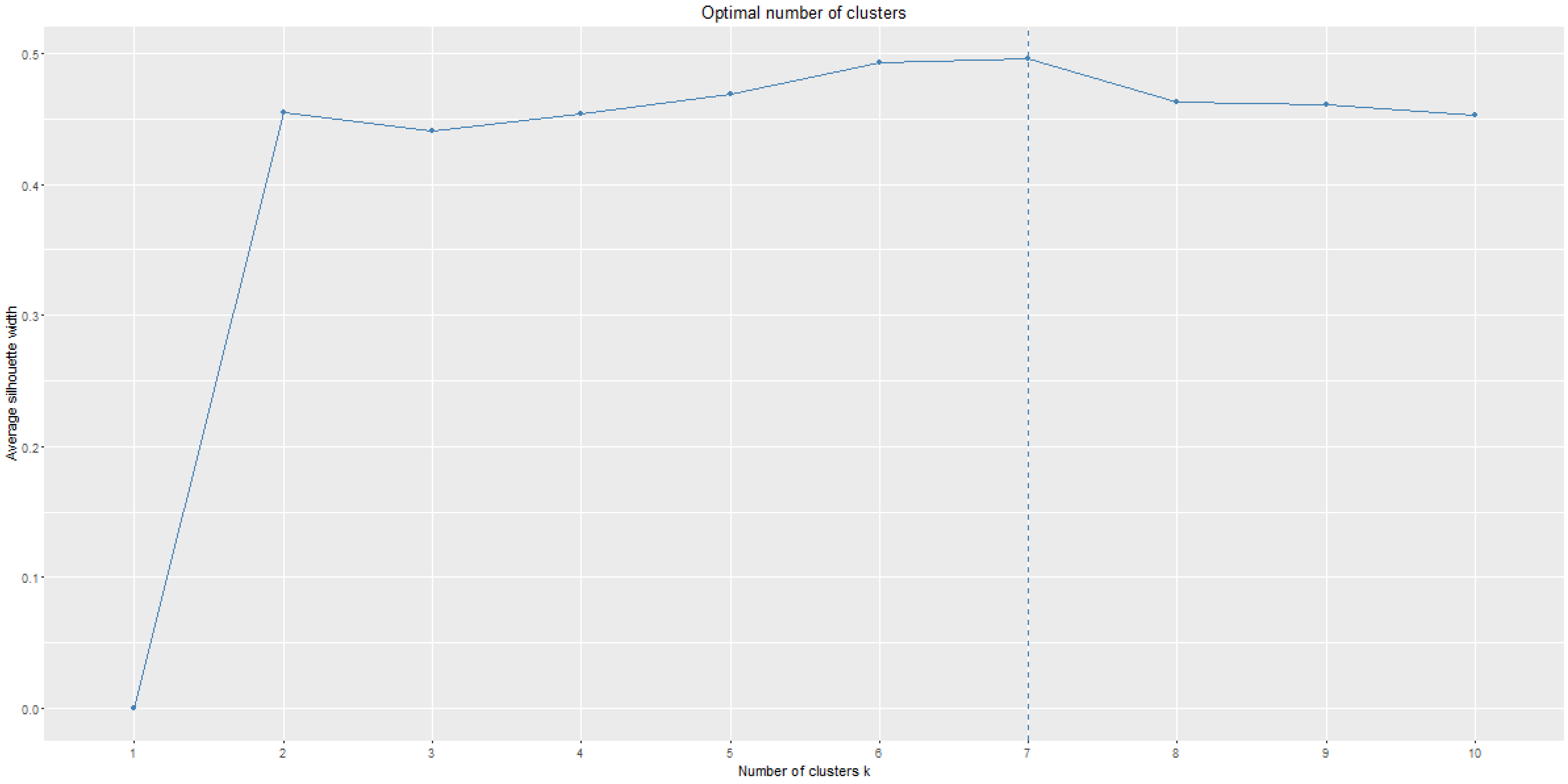

“Homology”

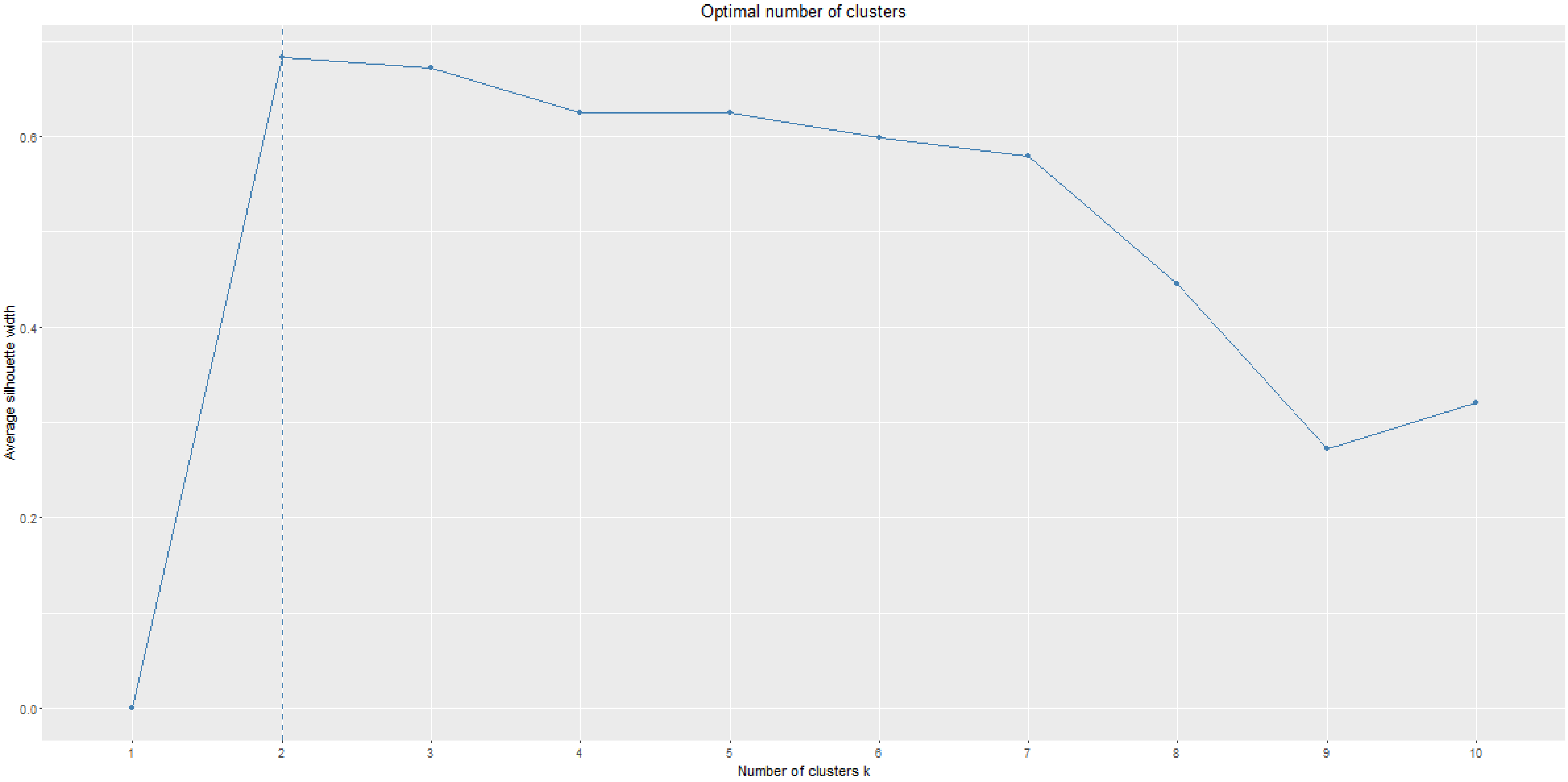
Optimal number of clusters
Average silhouette width
0.0
0.2
0.4
0.6
1
2
3
4
5
6
7
8
9
10
Number of clusters k
